# Life cycle progression and sexual development of the apicomplexan parasite *Cryptosporidium parvum*

**DOI:** 10.1038/s41564-019-0539-x

**Published:** 2019-09-02

**Authors:** Jayesh Tandel, Elizabeth D. English, Adam Sateriale, Jodi A. Gullicksrud, Daniel P. Beiting, Megan C. Sullivan, Brittain Pinkston, Boris Striepen

**Affiliations:** 1 Department of Pathobiology, School of Veterinary Medicine, University of Pennsylvania, Philadelphia, PA USA; 2 Present Address: Franklin College of Arts and Science, University of Georgia, Athens, GA USA

**Keywords:** Parasite biology, Parasite development, Cellular microbiology

## Abstract

The apicomplexan parasite *Cryptosporidium* is a leading global cause of severe diarrhoeal disease and an important contributor to early childhood mortality. Currently, there are no fully effective treatments or vaccines available. Parasite transmission occurs through ingestion of oocysts, through either direct contact or consumption of contaminated water or food. Oocysts are meiotic spores and the product of parasite sex. *Cryptosporidium* has a single-host life cycle in which both asexual and sexual processes occur in the intestine of infected hosts. Here, we genetically engineered strains of *Cryptosporidium* to make life cycle progression and parasite sex tractable. We derive reporter strains to follow parasite development in culture and in infected mice and define the genes that orchestrate sex and oocyst formation through mRNA sequencing of sorted cells. After 2 d, parasites in cell culture show pronounced sexualization, but productive fertilization does not occur and infection falters. By contrast, in infected mice, male gametes successfully fertilize female parasites, which leads to meiotic division and sporulation. To rigorously test for fertilization, we devised a two-component genetic-crossing assay using a reporter that is activated by Cre recombinase. Our findings suggest obligate developmental progression towards sex in *Cryptosporidium*, which has important implications for the treatment and prevention of the infection.

## Abstract (web summary)

Infection with *Cryptosporidium parvum* is a leading cause of severe diarrhoeal disease and childhood mortality worldwide. Using tools they recently developed to genetically engineer *Cryptosporidium*, the authors define life cycle stage-specific markers and generate reporter parasites, making life cycle progression and parasite sex tractable.

## Main

Diarrhoeal diseases account for 9% of global child mortality^1^ and infection with *Cryptosporidium* is a leading cause of severe paediatric diarrhoea^2^. Malnourished children are particularly susceptible to cryptosporidiosis, which results in recurrent or persistent infection and death^2–4^. *Cryptosporidium* is also an important cause of malnutrition^5^, and infection can result in lasting growth defects^6^. Even in high-income countries, outbreaks are frequent and more than 50% of waterborne infections in the United States is due to *Cryptosporidium*^7,8^. The current treatment of cryptosporidiosis is of limited efficacy for those patients who have the most urgent need of treatment^9^.

*Cryptosporidium* is a member of the eukaryotic phylum Apicomplexa and has a life cycle that alternates between asexual and sexual reproduction. However, in contrast to most other apicomplexans, the entire cycle occurs in a single host. Sex results in the production of oocysts, which are environmentally hardy meiotic spores. Sex and production of oocysts are therefore essential to transmission but may also play a role in the continued infection of the host^10^. Chronic infection could be sustained by asexual replication with facultative sex, driving host-to-host transmission. Alternatively, progression to sexual stages might be obligatory. *Cryptosporidium* oocysts are unique in that they mature within the host tissue and are autoinfective. Thus, they could reset the developmental cycle and maintain infection. Which of these two models applies is a fundamental, yet unanswered, question that has important implications for the disease and the development of drugs and vaccines. Here we develop molecular markers to observe and analyse the progression of the *Cryptosporidium* life cycle and use these markers to demonstrate that a block in fertilization limits parasite growth in culture, supporting a model of obligate sexual developmental progression to maintain infection.

## Results

### Using a reporter parasite to track *Cryptosporidium* life cycle progression

**Fig. 1:**
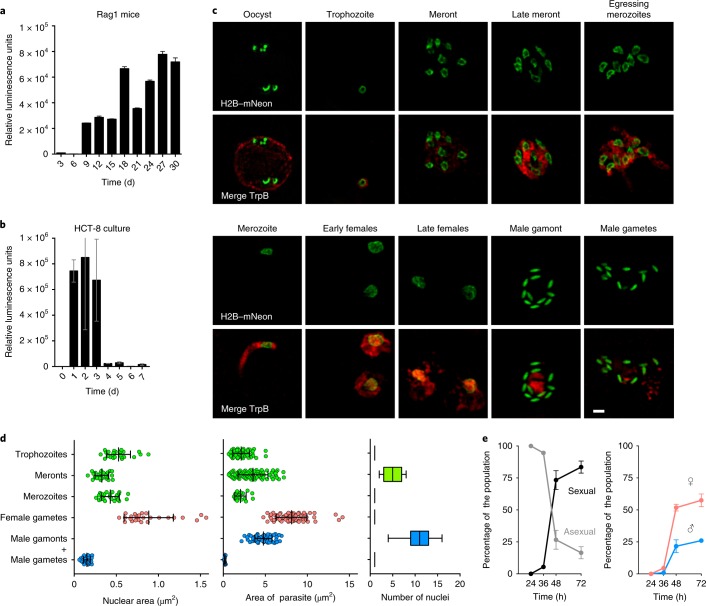
*Cryptosporidium* life cycle stages revealed by the H2B–mNeon transgene. **a**,**b**, *C. parvum* infection was monitored by luciferase activity in mice lacking mature T and B cells (**a**; faeces were measured every 3 d) and HCT-8 cultures (**b**). Data are mean ± s.d. from three independent biological replicates. **c**, HCT-8 cultures were infected with H2B–mNeon transgenic parasites and fixed at 24 h (‘Oocyst’, ‘Trophozoite’, ‘Meront’, ‘Late meront’ and ‘Egressing merozoites’), 36 h (‘Merozoite’) and 48 h (‘Early females’, ‘Late females’, ‘Male gamont’ and ‘Male gametes’) time intervals. Green, nuclei; red, cytoplasm (antibody against tryptophan synthase B (TrpB), cgd5_4560). This experiment was performed three times with similar results. Scale bar, 1 µm. **d**, Morphometric analyses of the size (*n* *=* 25) and number (*n* = 100) of nuclei and the area for each stage (*n* *=* 75) on the basis of the markers shown in **c**. The nuclear area (left) and total area (middle) of parasites stages are shown as mean ± s.d. of individual values represented as dots. The number of nuclei at particular parasite stages are represented as box plots (right). The box shows median and quartile range and whiskers represent extreme values. **e**, A time-course experiment in which stages were scored using the parameters defined in **d** revealed abrupt sexualization of cultures at 48 h into culture. Data are mean ± s.d. from three independent biological replicates.

In the absence of adaptive immunity, humans and mice develop long-lasting *Cryptosporidium* infections and the parasite replicates continuously (Fig. 1a). Immortalized epithelial cell lines such as Caco2, HT-29 and HCT-8 are readily infected, but growth ceases after 3 d and the infection cannot be maintained by serial passage^11^ (Fig. 1b). During this time period, morphological stages that are consistent with asexual and sexual development have been observed, and different sets of genes appear to be expressed in succession^12–14^. However, rigorous stage-specific markers are lacking. We therefore sought to engineer transgenic parasites that delineate life cycle progression and took advantage of well-documented changes in nuclear morphology of the parasites^10,15^. We introduced a fusion of *Cryptosporidium parvum* histone H2B (cgd5_3170) with the fluorescent reporter mNeon^16^ (Supplementary Fig. 1). HCT-8 cells infected with these parasites were fixed after 24 h and 48 h and then imaged by super-resolution structured illumination microscopy. All of the parasites showed nuclear fluorescence. We recorded morphometric data for each parasite and its nucleus (Fig. 1d) and were able to distinguish multiple stages. At 24 h, we observed trophozoites, which are small rounded intracellular stages with a single nucleus, and stages of increasing size with an unsegmented cytoplasm and two or four nuclei that we interpret as intermediate stages. We also observed mature meronts with eight nuclei, before and during egress, as well as free merozoites (Fig. 1c).

At 48 h, we observed sexual stages (we use the terms male and female gametes according to the convention of the extensive literature on sex in the malaria parasite *Plasmodium*^17^). Female or macrogametes had a single nucleus that was significantly larger (0.89 μm^2^) than the nuclei of asexual stages (0.43 μm^2^; *P* < 0.0001, unpaired Student’s *t*-test) and male or microgametes, which had dense, bullet-shaped nuclei (0.15 μm^2^; *P* < 0.0001, unpaired Student’s *t*-test). We found up to 16 of these nuclei in male or microgamonts (the precursor stage of the male gamete). We next conducted time-course experiments and assigned a stage to all of the parasites observed using the morphometric characteristics that are defined above. Initially, all parasites in culture were asexual meronts and trophozoites. After 36 h, the culture rapidly sexualizes, with gamonts and gametes representing >80% of all stages after 72 h (Fig. 1e).

### Gene expression in gametes is controlled by stage-specific promoters

**Fig. 2:**
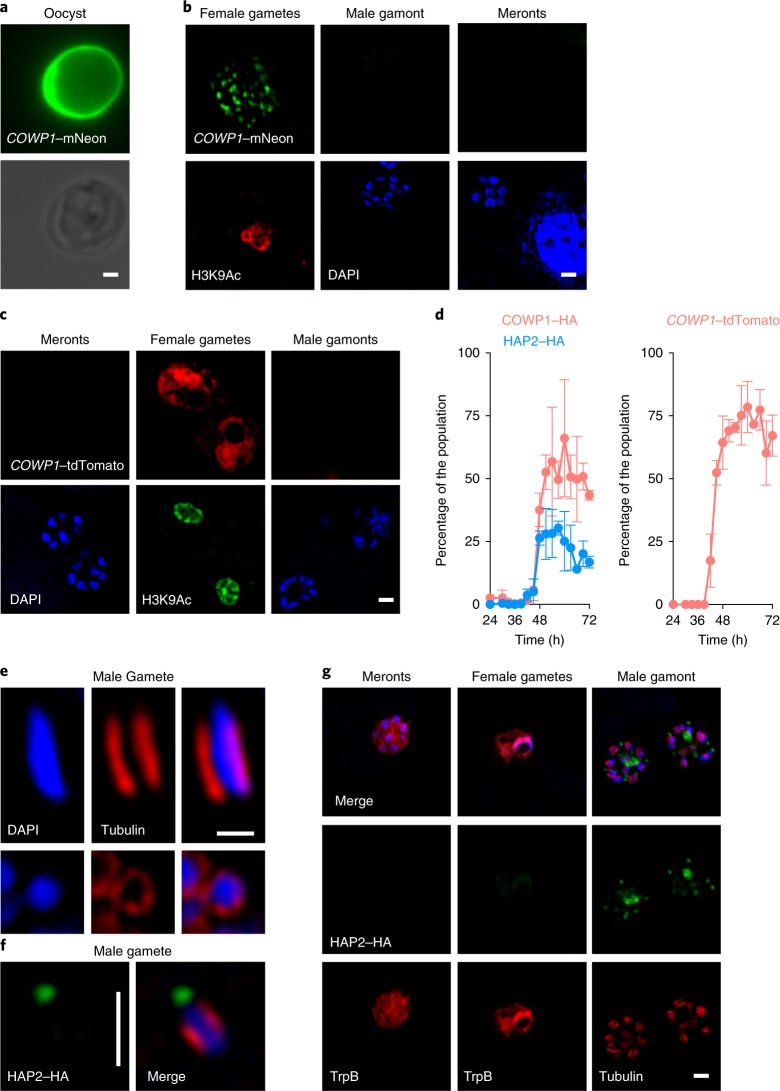
Exclusive molecular markers for the sexual stages of *C. parvum*. **a**–**d**, *C. parvum* were engineered to express COWP1–mNeon (**a**,**b**) and COWP1–HA (Supplementary Fig. 3) from the native locus or *COWP1-*promoter-driven tdTomato from the ectopic *TK* locus (**c**). Note the mNeon labelling of the wall in oocysts purified from infected mice and punctate labelling in female gametes observed in infected HCT-8 cells. Labelling becomes apparent after 42 h of culture and is never observed in asexual meronts or male gametes (**b**,**d**). The *COWP1* promoter alone is sufficient to confer female-specific expression to a reporter protein (**c**,**d**). Anti-H3K9Ac antibodies were used to label the nuclei of females because they stain poorly with 4,6-diamidino-2-phenylindole (DAPI). For the time intervals in **d**, cultures were infected with the indicated transgenic strains and triplicate coverslips were fixed and processed for immunofluorescence assays. Parasite stages were scored for HA staining, the mean ± s.d. percentage of HA-positive stages among all of the parasites is shown for three independent biological replicates. **e**, Male gametes show a characteristic array of microtubules around the nucleus after staining with anti-tubulin antibodies. **f**,**g**, When parasites were engineered to express HAP2–HA from the native locus, antibody staining revealed exclusive labelling of free gametes (**f**) and male gamonts (**g**). HAP2 labels a single pole per mature gamete. This staining becomes apparent after 42 h of culture (**d**, blue). All of the microscopy experiments shown in this figure were performed independently three times. Scale bars in **a**–**c**,**f**,**g**, 1 µm; scale bar in **e**, 0.5 µm.

To validate our stage assignments, we next defined exclusive molecular markers for gametes. The female gamete produces and stores components of the oocyst wall in wall-forming bodies that were previously described in related parasites^18^, and earlier studies identified *Cryptosporidium* oocyst wall protein-1 (COWP1)^19^. We tagged the COWP1 protein (cgd6_2090) by C-terminal insertion of either a fluorescent protein or an haemagglutinin (HA) epitope into the native locus (Supplementary Fig. 2a,b). Transgenic parasites showed strong labelling of the oocyst wall (Fig. 2a). When infected cell cultures were examined, no expression was apparent at 24 h, but numerous fluorescent parasites were observed at 48 h. These parasites had a single large nucleus and multiple small foci of COWP1 consistent with wall-forming bodies (Fig. 2b). We next observed COWP1 expression in parasites throughout a detailed time course in vitro using the HA-tagged COWP1–HA strain. Parasites expressing COWP1–HA closely matched the stages that we identified for female gametes using H2B–mNeon in terms of morphology and the proportion of the overall parasite population at the observed time points (Fig. 2d, Supplementary Fig. 3). To study what controls the stage specificity of gene expression, we placed fluorescent protein reporters under the control of the presumptive *COWP1* promoter region and ectopically expressed these constructs (Fig. 2c,d, Supplementary Fig. 2). Fluorescence (now cytoplasmic) was exclusively associated with female gametes and temporal expression matched that of the native locus, demonstrating that promoters, and thus probably transcription initiation, control stage specificity (Fig. 2d).

### Male gametes express HAP2 and appear at the same time as female gametes

Male gametes in most apicomplexans move with the aid of flagella, and the exclusive presence of these flagella in males provides numerous marker proteins. *Cryptosporidium* male gametes lack flagella but have a peculiar set of microtubules that are associated with and run along the length of their spindle-shaped nuclei^15^ that we visualized using super-resolution microscopy (Fig. 2e). We also identified a *C. parvum* homologue of hapless2 (HAP2; Supplementary Fig. 4), a class II membrane fusion protein that is required for gamete fusion in a range of organisms including *Plasmodium falciparum* and *Chlamydomonas reinhardtii*, and is expressed by the male or minus gamete, respectively^20^. We epitope tagged the C terminus of HAP2 and infected cell cultures with transgenic parasites. HAP2–HA labelling was found exclusively in male gamonts (Fig. 2g) and gametes and was restricted to one end of the polarized male gamete (Fig. 2f). Time-course experiments demonstrated the appearance of males after 42 h of culture (Fig. 2d). We note that both sexes emerge at the same time and at a male to female ratio of 1:2 for gamonts and 6:1 for gametes. We engineered multiple strains (within the HAP2 locus and ectopically) in an effort to drive the expression of fluorescent protein using the HAP2 promoter in a male-specific manner, but we did not observe any fluorescence.

### Defining stage-specific *Cryptosporidium* gene expression

**Fig. 3:**
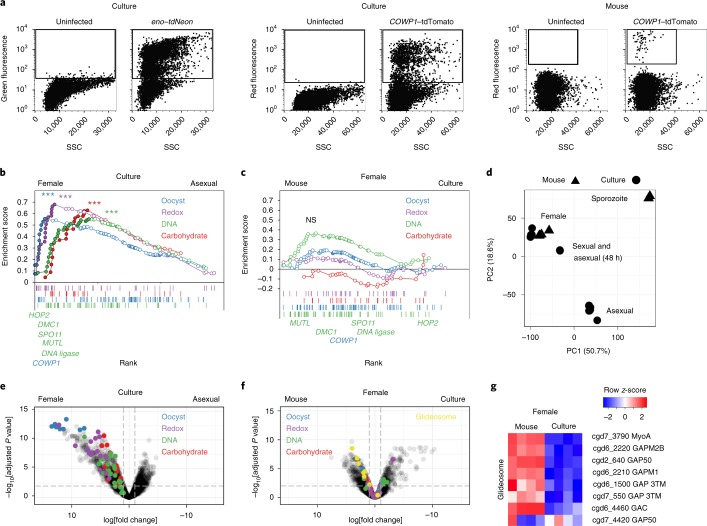
Isolation of parasite stages by cell sorting and RNA sequencing. **a**, Flow cytometry of infected cells with the indicated markers and origins. Gates used for sorting are shown as boxes. This experiment was performed twice. SSC, side scatter. **b**,**c**, Gene set enrichment analysis (GSEA) with multiple testing correction comparing cultured asexual and female parasites (**b**) or females sorted from mice or culture (**c**). Custom gene signatures were generated using Gene Ontology or community datasets available at CryptoDB (**b**; carbohydrate (GO:0005975; normalized enrichment score (NES) = 1.67, FDR-adjusted *P* = 0.004), DNA (GO:0006259; NES = 1.68, FDR-adjusted *P* = 0.005), redox (GO:0055114; NES = 1.99, FDR-adjusted *P* = 0) and oocyst wall proteome^23^ dataset (NES = 1.76, FDR-adjusted *P* = 0.001)). ***P ≤ 0.005. Enrichment analysis comparing between females was not significant (NS). *n* = 4 biological replicates per group. **d**, Principal component analysis of all RNA-sequencing datasets generated during this study (Supplementary Fig. 7). **e**,**f**, Volcano plots showing *C. parvum* genes that were differentially expressed between asexual and female parasites from culture (**e**) or between in vitro and in vivo female parasites (**f**). *n* = 4 biological replicates per group. Each symbol represents a *C. parvum* gene, those genes representing the leading edge from **b** are indicated by the colour according to the pathway that they act in. The horizontal dashed line shows an FDR-adjusted *P* value of 0.01; the vertical dashed lines indicate a log_2_-transformed fold change of −1 and 1, respectively. **g**, A heat map of glideosome components, which are indicated in yellow in **f**. *n* = 4 biological replicates per group.

To discover the genes associated with sex in *C. parvum*, we sought to isolate specific parasite stages. We developed flow cytometry protocols to sort infected cells on the basis of the expression of fluorescent proteins by the parasite (Supplementary Fig. 5). Figure 3a shows sorts from cell culture and mice, in which infected and uninfected cells are readily discernible. Next, we conducted mRNA sequencing experiments using cells sorted for *eno*-promoter-driven tdNeon (*eno*–tdNeon; Supplementary Fig. 6) and *COWP1-*promoter-driven tdTomato (*COWP1*–tdTomato) from 24 h or 48 h cultures to isolate asexual and female stages, respectively, as well as females from infected mice (Supplementary Fig. 7). We obtained between 5 million and 35 million reads for each sample, with 50,000 to 7,000,000 mapping to the *C. parvum* transcriptome, representing 2,500–3,400 of the 3,885 *C. parvum* genes (see Methods; Supplementary File 7).

Analysis revealed robust transcriptional differences between asexual and female parasites. The transition to female gametes was accompanied by a twofold or greater increase (false discovery rate (FDR)-adjusted *P* < 0.01) in the expression of 673 genes including *COWP1* (Fig. 3e, Supplementary File 2; 451 genes are downregulated). We compared these genes with those that are associated with female gametogenesis in *Plasmodium berghei*^21^ (this particular dataset was most comparable to our experiment) and found 72 shared orthologue groups that encompass 73 *C. parvum* genes and 81 *P. berghei* genes (~31% of the female *C. parvum* genes with an identifiable *P. berghei* homologue) as well as 595 *C. parvum*-specific genes. We also compared female-specific genes from *C. parvum* and *P. berghei* to gametocyte genes in *Eimeria tenella*^22^ and identified a set of 41 orthologue groups that contained 42 *C. parvum* genes, 49 *P. berghei* genes and 55 *E. tenella* genes (see Supplementary File 4 for the complete gene lists).

**Fig. 4:**
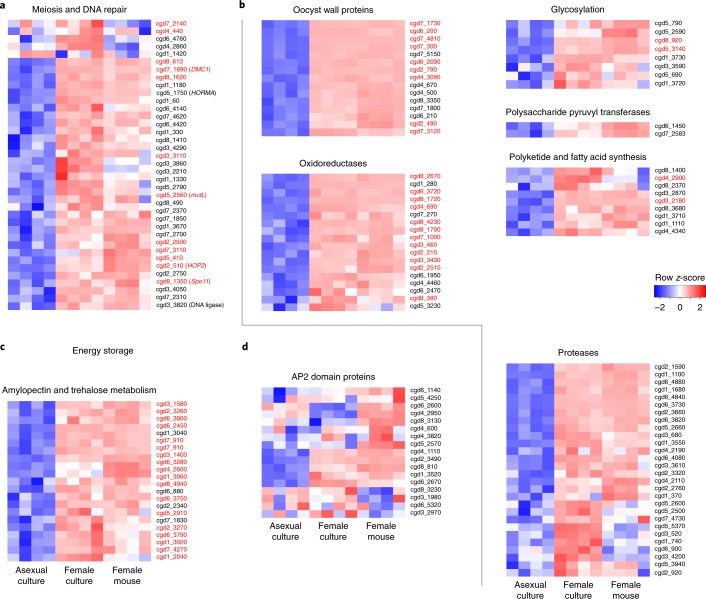
Female gametes express genes that are required for genetic recombination and oocyst formation. **a**–**c**, Heat maps illustrating expression of genes associated with specific molecular functions that are upregulated in females (generally results from in vivo and in vitro females concur, although there are some exceptions). Genetic recombination (**a**), oocyst environmental resilience (**b**) and energy storage (**c**); *n* = 4 biological replicates per group. **d**, Expression heat map for all *C. parvum* AP2 DNA-binding proteins. Note the pronounced difference identifying four genes upregulated in all females and two only in in vivo females. As we were unable to sequence males, we cannot formally exclude that some genes that show high female-specific expression may be upregulated in all sexual stages. Expression values are given as row *z*-scores and annotated genes list are provided as Supplementary Files. Genes from the leading edge in Fig. 3b are highlighted in red text.

Functional annotation of the *C. parvum* genome using Gene Ontology is very limited. We therefore assembled pathways using the Gene Ontology terms for DNA, carbohydrate, and oxidase and reductase metabolism, as well as a candidate oocyst wall proteome^23^ to conduct enrichment analysis (Fig. 3b). We found significant enrichment when comparing female with asexual parasites (FDR-adjusted *P* < 0.005). Figure 4 shows additional clustering on the basis of literature-based pathway annotation, those genes that were also found in the leading edge of the enrichment analysis are highlighted in red. *Cryptosporidium* is a haplont and meiosis is presumed to follow fertilization. Consistent with this view, we found that conserved eukaryotic factors of meiotic recombination—including DMC1, Spo11, HORMA and HOP2—were preferentially expressed in females as well as proteins with a probable role in meiosis-associated DNA repair, including the mismatch repair protein MutL and DNA ligase (Figs. 3b and 4a). We also note chromosome segregation and cell-division factors, including condensins, cohesins, stage-specific cyclins, cyclin-dependent and NIMA kinases, and cytoskeletal proteins. Overall, we identified 37 genes in this meiosis category and many of these are shared among apicomplexan females (Supplementary Fig. 8, Supplementary File 4).

*Cryptosporidium* oocysts remain infectious for months^24^ and female transcription provides candidate mechanisms of this resilience. Twenty-two enzymes that are required for the metabolism of amylopectin and trehalose are upregulated in females (Fig. 4c). Amylopectin is consumed^24^ by the sporozoites and the disaccharide trehalose may play a role in energy storage as well as serving to moderate osmotic stress^25^. *Cryptosporidium* oocysts are also highly resistant to chemical assault, including water chlorination^26^, due to a complex multilayered wall made of proteins, carbohydrates and lipids^27^. We identified 69 genes that are preferentially expressed in females and encode proteins with a probable role in oocyst wall synthesis, most of which have a predicted signal peptide. These include previously identified oocyst wall proteins and their homologues, numerous proteases (aspartic peptidases, serine proteases and subtilases) and protein modifiers, such as amine oxidases^22^, that serve to build or modify the proteinaceous components of the wall (Fig. 4b). Many protozoans have chitin and glucan cyst walls and the *Cryptosporidium* wall is labelled by various lectins, but no wall polysaccharide has been identified^27,28^. Similarly, we did not find a stereotypical chitin or glucan synthase; however, among female transcripts there are numerous glycosyltransferases. Interestingly, this set contains two polysaccharide pyruvyl transferases (cgd7_2583 and cgd6_1450) and a UDP-glucose dehydrogenase (cgd8_920) that were all recently linked to capsule synthesis in pathogenic *Acinetobacter*^29^, as well as proteins with lectin domains including chitin-binding proteins, which suggests a proteoglycan structure (Fig. 4b). Finally, we found that the two giant lipid synthases—polyketide synthase^30^ (cgd4_2900) and type I fatty acid synthase (cgd3_2180)—that were acquired by horizontal transfer from bacteria are specifically expressed in females. In *Mycobacterium tuberculosis*, these enzymes work in series to produce mycolic acids, which are key components of the mycobacterial wall. We chose two previously uncharacterized genes for experimental validation that were identified here as likely to be female specific (cgd7_4810 and cgd7_5140), and used CRISPR–Cas9 to attach a c-Myc epitope tag. Transgenic oocysts reacted strongly with the anti-c-Myc antibodies, and in culture we noted exclusive staining of female gametes (Supplementary Fig. 9).

*C. parvum* females show upregulation of genes required for meiosis, wall formation and oocyst persistence in vivo and in vitro without significant differences (Fig. 3c,f), which suggests an overall developmental competence of in vitro females (Fig. 4a–c). We also transcriptionally profiled sporozoites released from oocysts and infected bulk culture after 24 h and 48 h for comparison, and we found that sporozoites are moderately more similar to females in vivo than females in vitro (Fig. 3d, Supplementary Fig. 10). Overall, we conclude that a fraction of in vivo sorted cells moved beyond fertilization to the production of sporozoites. This is consistent with the expression of the protein components of the gliding machinery required for the motility of invasive stages that is only observed in vivo females (Fig. 3f,g). Among the genes for which expression is unique to females in vivo are also cgd5_2570 and cgd8_3130, which encode apetala2 (AP2)-domain proteins (Fig. 4d). AP2-type transcriptional regulators have been demonstrated in other apicomplexans to bind to specific genome features^31,32^, including promoters, and emerged as master regulators of life cycle progression. *Cryptosporidium* encodes a comparably small set of AP2s^33^ and many of these genes seem to be developmentally regulated, with expression patterns that differ greatly between asexual and sexual parasites and between in vitro and in vivo (Fig. 4d).

### Fertilization and meiosis occur within the host cell but only in vivo

**Fig. 5:**
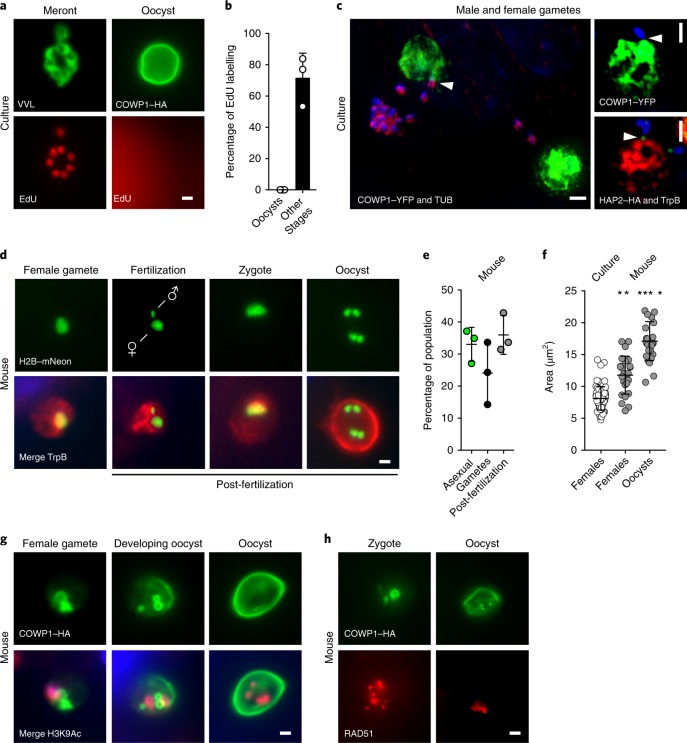
*Cryptosporidium* males locate females in culture, but fertilization and meiosis only occur in vivo. **a,b**, HCT-8 cells were infected with COWP1–HA *C. parvum* and after 36 h of infection, the nucleotide analogue EdU was added to the medium. Then, 12 h later, cells were click labelled and counterstained with anti-HA antibodies or *Vicia villosa* lectin (VVL; **a**). Cells were scored for nuclear EdU labelling (**b**); 100 stages were quantified for three biological replicates, and the experiment was performed twice. Data are mean ± s.d. **c**, Representative images of encounters between male and female gametes in culture; gametes were identified using the indicated transgenes or antibodies, and attached males are highlighted by arrowheads. YFP, yellow fluorescent protein. **d**–**h**, *Ifng*^−/−^ mice were infected with H2B–mNeon-expressing (**d**) or COWP1–HA-expressing (**g**,**h**) parasites, and intestines were sectioned and prepared for immunofluorescence assays and counterstained with anti-TrpB, anti-H3K9Ac or anti-RAD51 antibodies. Representative micrographs show progression of events following fertilization. Post-fertilization stages are abundant in vivo (**e**) and these stages were significantly larger than those found in vitro (**f**); each symbol represents a parasite, *n* = 25. For **e**, data are mean ± s.d. from three independent mice. For **f**, data are mean ± s.d.; the statistical analysis was performed using a two-sided Student’s *t*-test comparing cultured females with in vivo females (**P = 0.0023) or with in vivo oocysts (****P = 0.0001). All of the microscopy experiments shown in **d**–**h** were performed twice with similar results. Scale bars, 1 µm.

Gametes are plentiful in infected cell monolayers but oocysts are rare (<0.1% of all stages), which is consistent with our sequencing data. To determine whether these oocysts were produced de novo, we labelled newly synthesized DNA using the thymidine kinase analogue, EdU. As reported previously, *C. parvum* readily incorporates thymidine-kinase-activated tracers^34^. In vitro, none of the oocysts observed at 48 h after infection were labelled (Fig. 5a,b), indicating that these were from the inoculum and not formed in culture. We next studied whether male gametes mature and go on to fertilize females in culture. At 48 h after infection, we found that 10.6 ± 0.8% of all male gametes are released from gamonts and we frequently observed them to be attached to female gametes (Fig. 5c). At this time point, 16.1 ± 2.2% of all females identified by H2B–mNeon featured an attached male. Note that attachment was polar with the HAP2-marked end oriented towards the female. However, we did not observe female gametes with an internalized male gamete. To investigate how this compares with in vivo infection, in which we know that fertilization occurs, segments of the small intestine were recovered from infected mice, cryosectioned and processed for immunofluorescence. When using the H2B–mNeon line we rarely observed males attached to females, yet we frequently observed parasites that contained both an identifiable female and male nucleus (~5% of all stages; Fig. 5d). In vivo, zygotes and various intermediates of meiosis with one, two and four nuclei were readily observable and these post-fertilization stages accounted for 35% of all parasites (Fig. 5e). As these stages mature, they grow and their size significantly exceeds that of the female gametes observed in culture (*P* = 0.0023; Fig. 5f). We made very similar observations when studying the COWP1–HA strain in vivo. Meiotic divisions precede and partially overlap with the deposition of the oocyst wall (Fig. 5g). We also observed strong labelling of these stages with RAD51, a DNA repair protein that has an important role in homologous cross-over during meiosis (Fig. 5h).

### A genetic two-component assay demonstrates gamete fusion in vivo but not in vitro

**Fig. 6:**
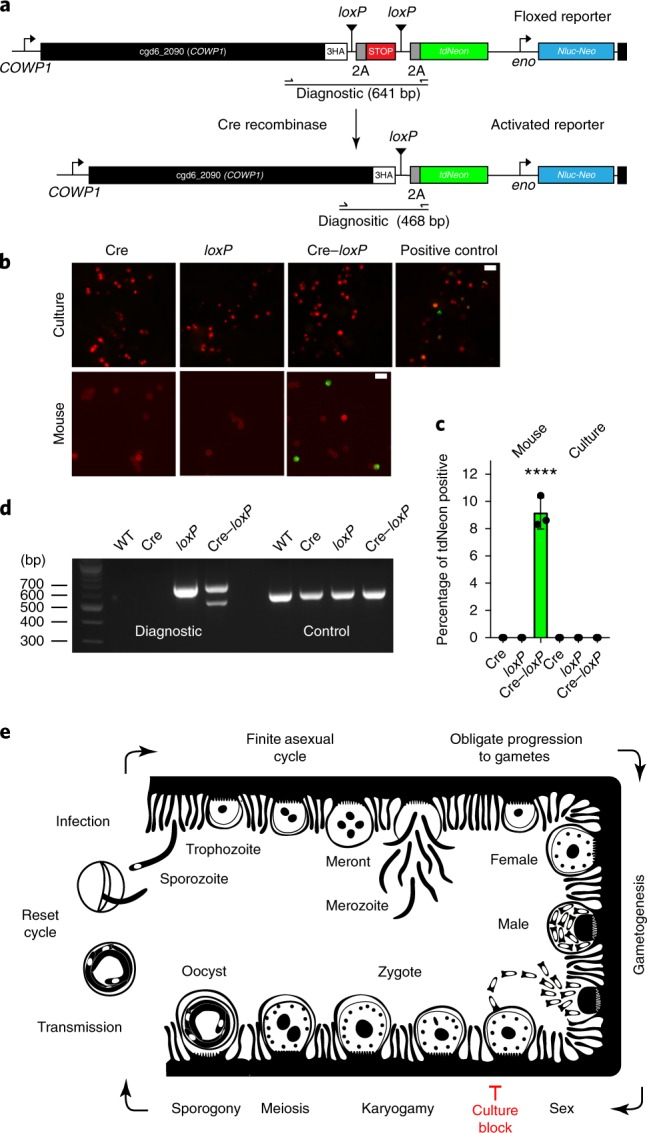
A genetic fusion assay demonstrates fertilization in vivo but not in vitro. **a**, To detect gamete fusion, we engineered two *C. parvum* strains, one that constitutively expresses Cre recombinase (Supplementary Fig. 11) and a second that carries a *tdNeon* reporter flanked by *loxP* at the *COWP1* locus. **b**, Cre-mediated excision of a terminator results in reporter expression. HCT-8 cultures and *Ifng*^−/−^ mice were infected with each strain individually or in combination. This experiment was performed twice. Cultured parasites were counterstained with anti-TrpB antibodies, oocysts with *Macula pomifera* agglutinin (both red) and scored for tdNeon expression. Scale bars, 10 µm. **c**, Three replicates were quantified for green fluorescence and 1,000 cells were counted for each replicate. Data are mean ± s.d. Green fluorescence was only observed after in vivo infection and only when both strains were present (****P *=* 0.0002, two-sided Student’s *t*-test). As a positive control, cells were infected with parasites that were crossed in vivo (indicated in **b**). **d**, PCR mapping of the floxed (diagnostic) and α-tubulin (control) loci using the primer pair shown in **a**. Genomic DNA was isolated from wild-type parasites as well as oocysts from the mouse infection experiments. Crossing resulted in a new amplicon that was consistent with precise Cre excision. This experiment was performed twice with similar results. **e**, Schematic model of the C*. parvum* life cycle that highlights the model of obligate progression to sex and the fertilization block in HCT-8 culture. We do not show type II meronts here, which are often depicted as an obligate step towards gametes. Although we observed meronts with four and eight nuclei, we did not find a quantitative link between the meronts with four nuclei and gametes (Supplementary Fig. 12).

Our experiments suggested a lack of fertilization in vitro. To test this rigorously, we devised a genetic assay for *Cryptosporidium* gamete fusion. We engineered a two-component system that produces a reporter signal only after cytoplasmic fusion of two strains. The first component is a driver strain that expresses Cre recombinase, an enzyme that excises DNA segments flanked by *loxP* recognition sequences (the 34 bp *loxP* sequence is absent from the *C. parvum* genome). Cre is driven by a constitutive promoter and detected in transgenics using a specific antibody (Supplementary Fig. 11). The second component is a strain carrying a *tdNeon* reporter in the *COWP1* locus linked by a 2A skip peptide. A terminator sequence flanked by *loxP* sites blocks expression, Cre-mediated excision will release the block (Fig. 6a). Mice were infected with each strain of parasite individually or with both in equal proportion. Only infection with both strains resulted in the shedding of green fluorescent oocysts (~10% of total oocysts from days 3–10 after infection; Fig. 6b–d). We next performed this assay in vitro and tested for tdNeon expression at 48 h and 72 h after infection. In contrast to mice, we did not detect expression of tdNeon in HCT-8 cells that were coinfected with Cre and floxed strains (Fig. 6b,c; *P* *=* 0.0002). Fluorescence was readily detected in our positive control, HCT-8 cells infected with oocysts obtained by Cre–*loxP* coinfection in mice. We conclude that gamete fusion occurs in vivo but not in vitro, and this block in fertilization prevents the formation of new oocysts and continued growth in culture.

## Discussion

The complex life cycles of parasites are among the most fascinating aspects of their biology. *Cryptosporidium* is a minute protist with a highly reduced genome, and yet it continuously transforms itself into a menagerie of specialized stages that amplify asexually, transform into male and female gametes, undergo fertilization and build a resilient spore. Here we trace and analyse this life cycle, label and isolate specific stages, and discover the genes that define these stages to provide a road map for the molecular dissection of parasite sex. We rigorously demonstrate that *Cryptosporidium* undergoes sexual differentiation in HCT-8 culture, but a block in gamete fusion prevents the development of new oocysts and the parasite cultures ultimately arrest (Fig. 6e). The cause of this block remains to be elucidated but seems to be linked to the host rather than the physiology of the parasite. This may be overcome partially by culture modalities that provide structured environments to transformed cells^35,36^ or by using stem-cell-derived models that self-organize into more complex organoids^37,38^. It is unclear whether this is due to differences in the infected host cells themselves or due to factors secreted by more complex assemblages. In all cases, improved growth is linked to appearance of oocysts. Overall, this is consistent with a model of obligate developmental progression and suggests that interventions targeting sex could potentially not only block transmission but also cure ongoing infection. Antibodies against HAP2 block fertilization in *Plasmodium*^20,39^, ‘contraceptive’ vaccination may therefore offer protection against cryptosporidiosis. This idea could be further tested by genetic ablation of the sex genes identified here, including *HAP2*. We define the stage specificity of numerous AP2 transcription factors, and the ablation or forced expression of these regulators^32,40^ enables engineering of the *Cryptosporidium* life cycle.

Fertilization, the fusion of two gametes, typically requires direct contact guided by interaction of surface-displayed fusion proteins and their ligands^41,42^. *Cryptosporidium* male gametes lack flagella and yet they are capable of locating females (Fig. 5c). Importantly, male gametes find females while they are inside an infected cell, and fertilization and subsequent meiosis are intracellular events (Fig. 6e). This could be guided by pheromones^43^ or, alternatively, by a ligand dispatched to the host cell surface by the female; translocons capable of such export are known in other Apicomplexa^44,45^. How the male overcomes the three membranes that separate it from its female counterpart is unknown, but this seems to be the very step that is blocked in vitro. In *Chlamydomonas*, membrane fusion and HAP2 are restricted to fertilization tubules that form in proximity to the basal body^46^. HAP2 in the *Cryptosporidium* male is restricted to the pole that faces the host cell carrying the female. Intriguingly, the male gamete also carries a polar basal body^15,47^ and adjacent membrane structures. These structures attract speculation and, with genetic and cell biological experiments now feasible, invite further mechanistic studies.

## Methods

### Plasmid construction

Guide oligonucleotides (Sigma-Aldrich) were introduced into the *C. parvum* Cas9/U6 plasmid^16^ by restriction cloning. See Pawlowic et al.^48^ for a detailed discussion of guide design for *C. parvum*. Transfection plasmids were constructed by Gibson assembly using NEB Gibson Assembly Master Mix (New England Biolabs).

### Generation of transgenic parasites

To generate transgenic parasites, 5 × 10^7^ *C. parvum* oocysts Iowa II strain (obtained from Bunchgrass Farms or the University of Arizona) were incubated at 37 °C for 1 h in 0.8% sodium taurocholate to induce excystation. Excysted sporozoites were then transfected using an Amaxa 4D Electroporator (Lonza) with parasites suspended in SF buffer using program EH100 and 50 µg of each Cas9/U6 plasmid and PCR repair construct. The repair encodes the neomycin phosphotransferase drug-selection marker fused to a nanoluciferase reporter flanked by 50 bp homologous regions to guide insertion into the parasite genome. *Ifng*^−/−^ mice were infected with transfected parasites by oral gavage. Stomach acid was neutralized with 100 µl of 8% NaHCO_3_ solution by gavage before infection. Note that this modification replaces surgery-based infection and significantly streamlines the protocol. Stable transformants were selected with paromomycin, given to mice ad libitum in their drinking water (16 mg ml^−1^) and parasite shedding was monitored by measuring nanoluciferase activity in the faeces of infected mice.

To purify transgenic parasites from collected faeces, we used sucrose flotation followed by a CsCl gradient^48^. In brief, collected mouse faeces were homogenized in tap water using a LabGen 125 homogenizer (Cole-Parmer) and filtered through a 250 μm mesh filter. This filtrate was diluted 1:1 with a saturated sucrose solution (specific gravity, 1.33) and centrifuged at 1,000*g* for 5 min. The supernatant was collected, resuspended in 0.85% saline solution and overlaid onto CsCl solution (specific gravity, 1.15) and centrifuged at 16,000*g* for 3 min. Purified oocysts were collected from the saline–CsCl interface and resuspended in cold PBS.

### Immunofluorescence assay

HCT-8 cells were infected with bleached and washed oocysts. Infected cells were maintained in RPMI-1640 medium (Sigma-Aldrich) containing 1% fetal bovine serum. Infected cells were fixed with 4% paraformaldehyde (Electron Microscopy Science) in PBS and then permeabilized with PBS containing 0.25% Triton X-100. Cells were blocked with 3% bovine serum albumin (BSA) solution, followed by incubation with primary antibodies. Cells were washed with PBS and then incubated with appropriate fluorophore-conjugated secondary antibodies and counterstained with DAPI. Coverslips were then mounted on glass slides with fluorogel (Electron Microscopy Science) mounting medium.

For in vivo staining, infected mice were killed and the small intestine was resected and flushed with 10% neutral buffered formalin (Sigma-Aldrich), then ‘swiss-rolled’ and fixed overnight in 4% paraformaldehyde followed by an overnight incubation in 30% sucrose solution. Samples were embedded in OCT medium (Tissue-Tek, Sakura Finetek) and cryosectioned. Tissue sections were blocked with 10% BSA and 0.1% Triton X-100 in PBS. Sections were stained with antibodies in PBS with 0.1% Triton X-100 as described above, counterstained with DAPI and mounted.

Super-resolution structured illumination microscopy was conducted using a Carl Zeiss Elyra (UGA Biomedical Microscopy Core) or a GE OMX (PennVet Imaging Core) microscope. Widefield microscopy was performed using a Leica LAS X microscope (PennVet Imaging Core) and images were processed and analysed using Carl Zeiss ZEN v.2.3 SP1, GE Softworx and NIH ImageJ software.

### Cre–*loxP*-based fertilization assay

To measure sex between two different strains, *Ifng*^−/−^ mice were infected with 50,000 oocysts of either the Cre or COWP1–HA flox tdNeon strain or coinfected with both the strains. Oocysts were purified from faecal samples that were collected at days 3–10 after infection. Oocysts were fixed with 4% PFA, stained with biotinylated *M. pomifera* agglutinin^49^ (Vector Laboratories), washed and incubated with Streptavidin-594, and settled onto poly-l-lysine (Sigma-Aldrich) coated coverslips before mounting with fluorogel. The same strains were used to infect HCT-8 cells. HCT-8 cells infected with oocysts obtained from Cre × COWP1–HA flox tdNeon coinfection were used as positive controls. Cells were fixed 48 h and 72 h after infection and parasites were stained using rabbit anti-TrpB antibodies.

### EdU labelling to detect DNA synthesis

HCT-8 cells were infected with 100,000 oocysts of the COWP1–HA strain. EdU was added to cultures 36 h after infection to a final concentration of 10 µM and cells were fixed 12 h later. A Click-iT EdU Alexa-Fluor 594 kit (Thermo Fischer Scientific) was used to label incorporated EdU. Parasites were stained with anti-HA antibody or fluorescein-conjugated *Vicia villosa* lectin (Vector Laboratories).

### Flow sorting of intracellular stages and RNA extraction

HCT-8 cells grown in 6-well plates were infected with 300,000 oocysts of *eno*–tdNeon (constitutive reporter strain) or *COWP1*–tdTomato (female reporter strain). Infected cultures were trypsinized with TrypLE Express Enzyme (Thermo Fischer Scientific), extensively washed with PBS, passed through a 40 µm filter (BD Biosciences) and pelleted. Cells were resuspended in 400 µl of buffer and sorted using a BD FACSJazz sorter (BD Biosciences). Uninfected HCT-8 control cells were used to gate on the singlet host cell population. Then, 10,000 positive cells were directly sorted into the RLT lysis buffer of the micro RNA extraction kit (Qiagen).

Four *Ifng*^−/−^ mice were infected with 200,000 oocysts of *COWP1*–tdTomato reporter strain. Mice were killed 2 d after infection, the small intestine was resected, cut into small pieces and incubated in RPMI-1640 medium containing 10% FBS, 25 mM HEPES, 5 mM EDTA, 50 µM β-mercaptoethanol and 0.145 mg ml^−1^ dithiothreitol for 20 min. The cell suspension was filtered through 70 µm and 40 µm kitchen-mesh filters (BD Biosciences). Cells were pelleted, resuspended in buffer, stained with anti-CD45.2 antibodies and sorted. Intestinal cells isolated from uninfected mice were used as controls. Then, 1,000 tdTomato-positive cells from each replicate were sorted directly into 350 µl of RLT lysis buffer.

The Qiagen micro RNA extraction kit was used to extract RNA from sorted cells. RNA was finally eluted in RNase-free water and samples were then stored at −80 °C.

### RNA sequencing of sorted cells

cDNA was generated using the SMART-Seq v4 Ultra Low Input RNA Kit (Takara Bio USA), and barcoded, sequence-ready libraries were prepared using the Nextera XT DNA Library Preparation Kit (Illumina). Total RNA and libraries were quality checked and quantified on an Agilent Tapestation 4200 (Agilent Technologies) and Qubit 3 (Thermo Fischer Scientific), respectively. All of the samples were pooled and single-end reads were generated using an Illumina NextSeq 500 sequencer.

### RNA sequencing of sporozoites and 24 h and 48 h infected bulk cultures

Oocysts from the Sterling laboratory (University of Arizona) were first induced to excyst through resuspension in 0.8% sodium deoxytaurocholate and incubation at 37 °C for 2 h. RNA from the released sporozoites was then isolated using an RNeasy Mini Kit (Qiagen) following the manufacturer’s instructions. Each biological replicate was isolated from 40 million sporozoites. For the 24 h and 48 h in vitro time points, oocysts from the Sterling laboratory (University of Arizona) were first treated with dilute household bleach (1:4 in dH_2_O) for 10 min on ice to sterilize them. The oocysts were then washed twice with cold PBS and resuspended in 0.8% sodium deoxytaurocholate and incubated for 10 min on ice. Oocysts were washed once more with cold PBS and then used to infect HCT-8 cell monolayers grown to 80% confluency in a 24-well plate. For the 24 h time points, 1 million oocysts were infected into each well with three biological replicates. For the 48 h time points, 100,000 oocysts were infected into each well with three biological replicates. During infection, standard RPMI growth medium was used supplemented with 1% fetal bovine serum. RNA from infected cells was isolated using an RNAeasy Mini Kit (Qiagen) according to the manufacturer’s protocols. Sequencing libraries for sporozoites and in vitro time points were prepared using a Nextera XT DNA Library Preparation Kit (Illumina) and 150-bp paired-end reads were collected using an Illumina MiSeq (Illumina).

### RNA-sequencing analysis

Raw reads were to the *C. parvum* Iowa II reference (Ensembl, ASM16534v1) using Kallisto v.0.45.0 (ref. ^50^). All subsequent analyses were carried out using the statistical computing environment R v.3.6 in RStudio v.1.1.463 and Bioconductor. In brief, transcript-level quantification data were summarized to genes using the tximport package and data were normalized using the TMM method (implemented in EdgeR). Only genes with more than 10 counts per million in at least 3 or 4 samples (depending on the analysis) were carried forward for analysis. Precision weights were applied to each gene on the basis of the mean–variance relationship using the VOOM function in Limma. Linear modelling and Bayesian statistics carried out in Limma were used to identify differentially expressed genes with a FDR-adjusted *P* of ≤0.01 and an absolute log_2_-transformed fold change of ≥1 after correcting for multiple testing using the Benjamini–Hochberg procedure. When necessary, batch correction was carried out using the empirical Bayes-moderated adjustment for unwanted covariates function (empiricalBayesLM) in the WGCNA package. All code used in these analyses is available in Supplementary File 7 and on GitHub (https://github.com/dpbisme/CryptoSex_manuscript). For *P. berghei*, files were downloaded from the NCBI Sequence Read Archive BioProject ID: PRJNA374918 (ref. ^21^) and forward reads were mapped as described above for the sorted *C. parvum* samples to the *P. berghei* reference transcriptome (Ensembl, PBANKA01). For *E. tenella*, differentially expressed gametocyte genes were obtained from Walker et al.^22^. Cross-species comparisons and orthologue identifications were performed using EuPathDB (https://eupathdb.org/). See Supplementary File 7 for full details, including a link to all of the code used for the RNA analyses performed here.

### Functional enrichment analysis

GSEA was carried out using GSEA software^51^. Four custom gene signatures for *C. parvum* were generated using Gene Ontology or community datasets available at CryptoDB (https://CryptoDB.org). A 28-gene signature for ‘carbohydrate metabolism’ was generated using the Gene Ontology term GO:0005975. A 63-gene signature for ‘DNA metabolic process’ was generated using GO:0006259. A 48-gene signature for ‘oxidation–reduction’ was generated using GO:0055114. An 85-gene oocyst signature was generated by using CryptoDB to mine a published oocyst wall proteome dataset from Truong and Ferrari^23^ to retrieve only genes that had ≥20 unique peptide sequences per sample. All four signatures were used for GSEA with 1,000 permutations of gene sets to generate *P* values, and multiple testing correction was applied to generate FDR-adjusted *P* values. GSEA results were used to create enrichment plots in DataGraph v.4.4 (Visual Data Tools).

### Statistical methods

GraphPad PRISM was used for all statistical analyses. When measuring the difference between two populations, we used a standard Student’s *t*-test. No statistical tests were used to predetermine sample size and no animals were excluded from results.

### Animal ethics statement

All of the protocols for animal experimentation were approved by the Institutional Animal Care and Use Committee of the University of Georgia (protocol A2016 01-028-Y1-A4) and/or the Institutional Animal Care and Use Committee of the University of Pennsylvania (protocol number 806292). four-week old *Ifng*^−/−^ and *Rag1* knockout female mice strains of *Mus musculus* were used for all of the experiments. No statistical tests were used to predetermine the sample size of mice used for experiments. Mice were not randomized and investigators were not blinded before any of the experiments.

### Reporting Summary

Further information on research design is available in the Nature Research Reporting Summary linked to this article.

## Supplementary information

Supplementary Information Supplementary Figs. 1–12 and Supplementary Table 1.

Reporting Summary

Supplementary File 1 Summary of sequencing information. Multi-tab excel file with information about total reads and reads mapped for each sample. Each tab contains the information for separate experiments.

Supplementary File 2 Differentially expressed genes. Multi-tab excel file containing the expression data for differentially expressed genes between sorted asexual and female infected cells in vitro and between female infected cells in vitro versus in vivo. Expression given as log_2_-transformed and normalized counts per million.

Supplementary File 3 Clusters of co-expressed genes. Multi-tab excel file containing expression data for each of the clusters of co-expressed genes outlined in Supplementary Figs. 8a and 9b. Expression given as log_2_-transformed and normalized counts per million.

Supplementary File 4 Conservation of female or gametocyte expressed genes across three apicomplexan species. Multi-tab excel file containing genes that are shared or uniquely expressed between *C. parvum* females, *P. berghei* females and *E. tenella* gametocytes, as indicated in Supplementary Fig. 8b.

Supplementary File 5 Shared expression of top 500 genes among sorted *C. parvum* stages. Multi-tab excel file containing overlapping or unique genes in the top 500 expressed genes in asexual parasites in vitro, female parasites in vitro and female parasites in vivo, as indicated in Supplementary Fig. 8c.

Supplementary File 6 Shared expression of sporozoite genes. Multi-tab excel file containing genes expression by sporozoites compared with gene expression 24 and 48 h after infection and their overlap with either in vitro or in vivo females as indicated in Supplementary Fig. 9c,d.

Supplementary File 7 Supplementary Code.

## Data Availability

RNA-sequencing data generated in this study are available from GEO database repository under accession number GSE129267. Additional RNA-sequencing data that support the findings of this study are available from the NCBI Sequence Read Archive BioProject ID: PRJNA374918.

## Code availability

All code used in these analyses is available in Supplementary File 7 and on GitHub (https://github.com/dpbisme/CryptoSex_manuscript).
