# Supplementary material for: Life cycle progression and sexual development of the apicomplexan parasite Cryptosporidium parvum
Source: Nat Microbiol. 2019 Sep 2;4(12):2226–36. doi: 10.1038/s41564-019-0539-x (PMC6877471; doi:10.1038/s41564-019-0539-x)
Supplement: Supplementary file 1 — Supplementary Figs. 1–12 and Supplementary Table 1. [file 41564_2019_539_MOESM1_ESM.pdf]

nature microbiology

SUPPLEMENTARY INFORMATION
https://doi.org/10.1038/s41564-019-0539-x

In the format provided by the authors and unedited.

Life cycle progression and sexual development of the apicomplexan parasite *Cryptosporidium parvum*

Jayesh Tandel[1], Elizabeth D. English[1], Adam Sateriale[1], Jodi A. Gullicksrud[1], Daniel P. Beiting[1], Megan C. Sullivan[1], Brittain Pinkston[1,2] and Boris Striepen[1]*

[1]Department of Pathobiology, School of Veterinary Medicine, University of Pennsylvania, Philadelphia, PA, USA. [2]Present address: Franklin College of Arts and Science, University of Georgia, Athens, GA, USA. *e-mail: striepen@upenn.edu

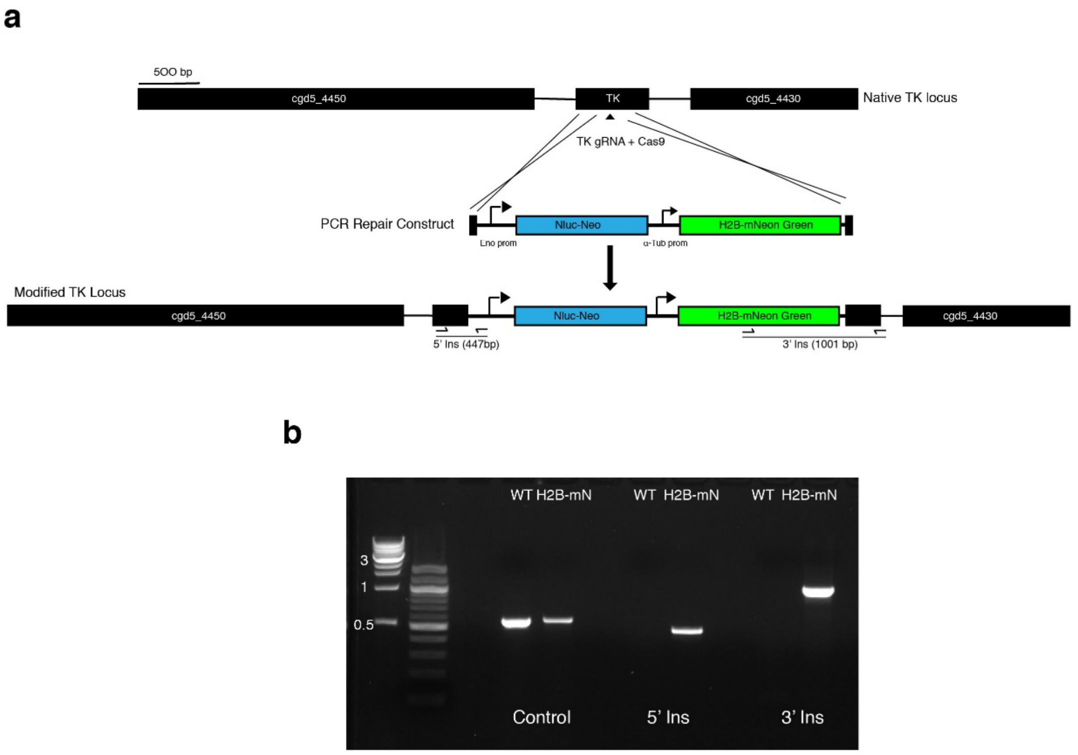

Supplementary Figure 1: Construction of a *C. parvum* strain expressing Histone H2B-mNeon. (**a**) Map of native *C. parvum* H2B locus, the targeting construct and the modified locus indicating the CRISPR/Cas9 induced break and areas of homologous recombination. (**b**) PCR analysis demonstrating successful insertion into the TK locus mapping the regions of 5’ and 3’ cross over, see (**a**) for the respective sizes of the predicted amplicons. This experiment was performed two times with similar results.

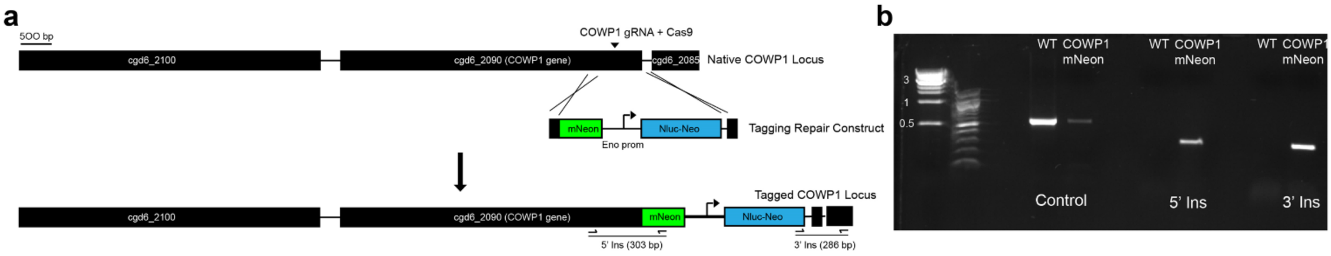

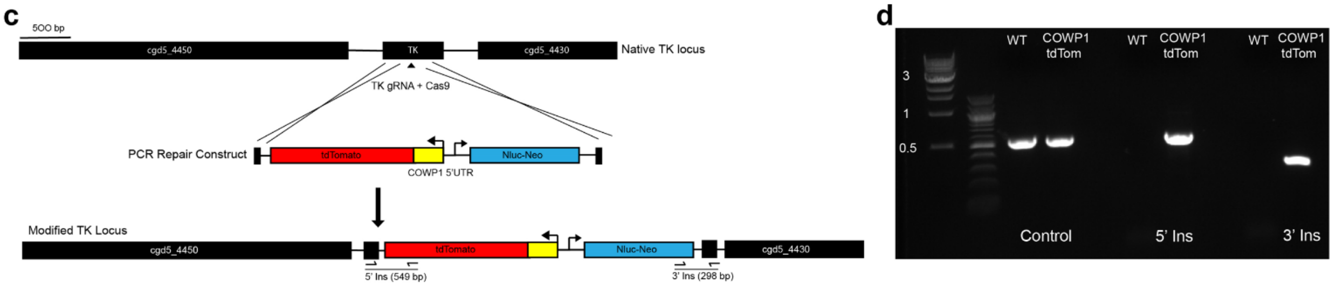

Supplementary Figure 2: Construction of *C. parvum* COWP1-mNeon and COWP1-tdTomato strains. (**a** and **c**) Maps depict the native loci, repair constructs and modified loci for generating respective strains. (**b** and **d**) PCR analyses confirming successful insertions of repair constructs for COWP1-mNeon and COWP1 tdTomato respectively (see plasmid maps in (**a**) and (**c**)) for amplicons for the predicted 5' and 3' insertion sites. This experiment was performed two times with similar results.

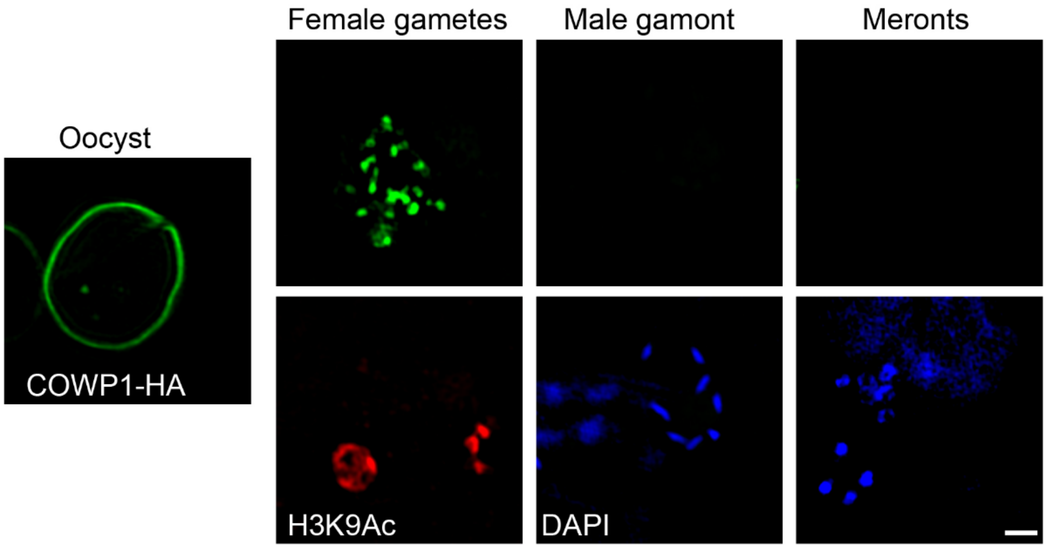

Supplementary Figure 3: Immunofluorescence analysis of female-specific expression of COWP1-HA. Note localization of COWP1-HA to the wall of oocysts and to punctate structures in female gametes. No staining is observed in asexual meronts or male gamonts. Female gametes were counterstained with an H3K9Ac antibody highlighting the nucleus. Males and meronts were identified by DAPI staining. Note that the large transcriptionally active nucleus of female gametes which stains poorly with DAPI (Scale Bar= 1 µm). This experiment was performed three times with similar results.

a

ATGGAGAAAATTATTTATTTATTATTAATATCAATAAACTGGTTGGTAGGAAATGCTATTTCACAGTTAATTGTGTCAGAACAAGAAATGTGTATGAATGGACAATGCAAAAAAATGTTG
AACGGTCATTTAGTTATACAGAATAATCAGgtaggcttattttttttttagtaaaataataattgaaaattaaatattttgaaattaattaattttatagGATGTATCGTTGAATTTTGGATCATCAGAAGTAAATGGA
AAGAATGCAAAAGAAGCAGACCCAGGTTCTAATGGATTTAAGATTACAATAAAGAAGTCTCCAATATTAATTGCATATGATTTGAGTTATATTTCAACTTTATATCATGATTTTCAAGAAG
ATATAAAATTGTTTTCGTTTACTCAAGTTGAAGGTGACCCATCTAGATTTTGTAATCATGAATTGAGAAGTTGTCAGAAAGTGAGAGACGAGTATTTATATGGAACACCAAATTgtaagtttt
aataaattattttgaatttagcaatatcttaataaaacatatttaaaatgatttataGTGCCACCAGGTCAAAAAAATTGGACTCAAGAAACTCCAATACCACCTGGATCACAAGGATCGTGTTGTTGGTGTCC
AGAATTATATGTAACGgtaagttcaattgaattaaagtgctggaggaggggctttttattagggattaaggtttttatattagagaaattaatgacatatttactcccaattaaataattttttttttttagATAAATACTGATGCACCA
TTTTCAAGAGCTACATTACATTGTTCATGGATACgtaagtttaatatattacaaatggaaataatttattgaactattttaaactatttatagagCAAAGAATGTGGTGGAATGTTTACTTATCGAAGTCATGCCC
AGTTTGGATACCACCATGGTGGACAGCTTTTAGAATAGGTGGATGGAACTGGCAATACTCATTAGAGGTTGAATTATCTTGGTTTAGTCCAACAGAATCATCAATTAATAAGTTATCAAG
TACAGAATTGGAAAATATGGAAAATGAATGTAAGAAAGAAAATAAGGATTCCACAATAGATTGTTCAAGATTAAGGCATAAAGAATCAGGAATTCAGACTTCTGTACATACATTAAATT
CATCGTCTCCATCATTCTATGATCCAAATTTTGGAGCTTCAGTACAGGTAATAAGCTCAGGACCACCGTTTGGGAGTGCTAATGCAAAGGATTTGAATGGTTATTACATGTTACAACCAAC
ATTTTCACCAAAAGGGATGCCTGCTAGTATTTCAATTCCTCCTTTAAGAAGTGGGTGTGGAAAAGCTTCAAAAAACCAAACAGAAGAGGAAATAAATGATTGTTTAAAGCCAACATTAAT
TATTCCTCCAGAAAATGCAGACTTTACAGGAGTTTCATGTGATAAGATAGGAACAAGTGTTCATACTTGGAGTTCTGTGAACGGTAGATTTTGCTATCATCCACCTGGTACTTGTCAAAG
AGCTCAGATAGCTCACTTTTATAAGAAAGTTATAGAAGATCATTCACTTGGAAAGATTTCACAATATTCAGTAAGAGCACAAAATTCTGGTTCTCCACAGTTGATTTTGGATTCATTAGGA
GAAATTGGTCATGAGGAGATGGATCAAAATGATATGGGAAATATAACTAATATACAATCACGCAGATTCTTTTTGGGATATAATTTTGATTCAATCTTTGACACAGAAATAATGTTCTCA
GTCGAAGCTTCTTCTGTGTCTTGGGTAGCAACATCTTCTCCTGGAATTATTACATTTATAGAACCACCACCTTTGGAGGCTTGCACAGCAATGAGTAGTTTTGGCTGTCCTCTAAAGGTTT
ATATTAAGAATAGTGgtaagtttgaatatatatatatatacatttcgaattgaattgaaaataacttaacaaaaatattctatagggGATATTGATTCAGGTTTTGTAGTTCAAATACCTTATTGTACAAAGTCAGGA
GTACAAACAAGTGAGgtaggtttgtatttaattcattcaaatttatataattaattaatttaggttgatCCAATAATGGCACAAACAAAGAAGATTAAAGCCAATTCAATAGGAATTTTTACTTTTGTATTGGG
AGTTTCCGTATTAACTGGGAGTGAATATAAGTGCGTGgtaagtatatatatatttaatgcatccaatatatgcatgcatacaataaatgcatgcatgcaatatattggtaaacactattaaataacaatattaaatattatttctat
tcaaataatagGTTGTGTTGTATAATTCATTTTCAGTTCAACTGGATCAGAATATGTTCACTTTTTCAACAACTTCTACAACGgtaagtttatttgaaagaagatatcgtttttgaatcttaatattaattattaatat
aaatattttgatatcaaattgaaaatttaagatttcaattGCAAGTTCAGATTCAATAATTTCTGATGTAGTAGTGAATCAAACTTCGTTAACAAACAAAAAGTTTGATTCTATGTTTACAGGAAATAAAT
GTATTTCTTGTGGATTAAATTTTTGGTGTTTAATAGCAAATagtaagttaaaaatgaatcattcttttaaaatttaaaataattaaaataaATTTCAGTTAAAGATTGTATTATGTTAGGTATTAAATGTATA
GGGATAATACTTGGATTGATAATAGGATTTGTTATATTATTAAAGTTGATAAAGATAATACTTGAACTACGTAAAAATAAGAAAATTGAAACAAGGATAATTAATAATAGCCAAGAAAG
TTTAAGTCAAGGTATTAATAGGAAGAATTGA

b

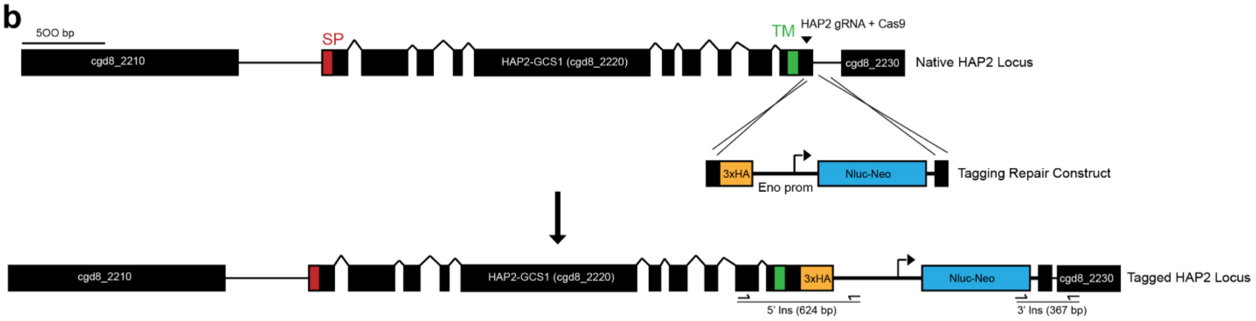

c

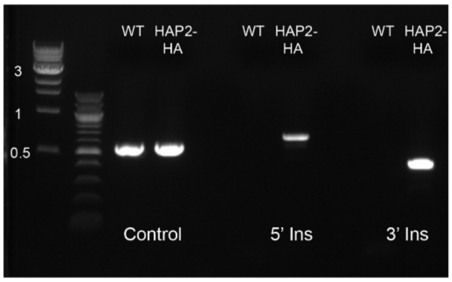

Supplementary Figure 4: Construction of an HA tagged *C. parvum* HAP2. (**a**) Only a fragment of the HAP2 gene (cgd8_2220) is annotated in the publicly available *C. parvum* genome sequence. We used RNA sequencing data and RT-PCR analysis to correct the gene model. While most *C. parvum* genes lack introns (highlighted in lower case), they are abundant in HAP2 as shown by the locus map in (**b**)**.** The protein was tagged with an HA epitope in the native locus. (**c**) PCR analysis confirmed successful epitope tagging of the HAP2 gene. This experiment was performed two times with similar results.

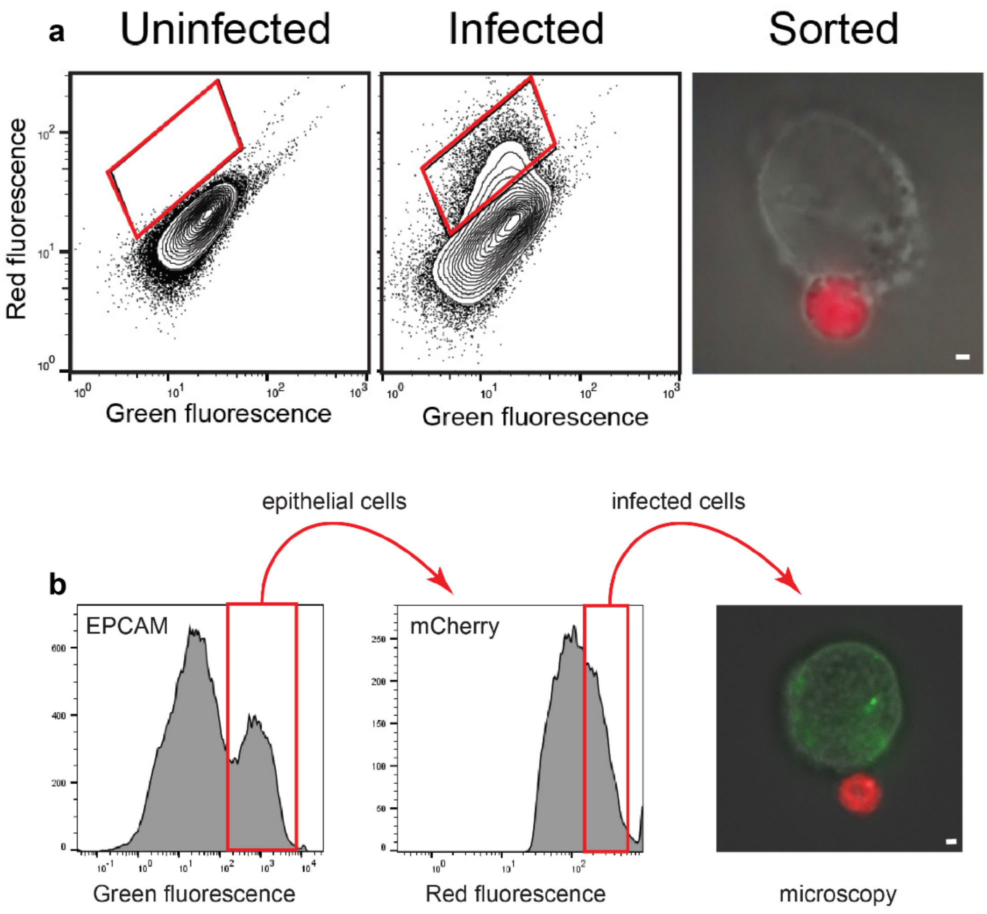

Supplementary Figure 5: Microscopic validation of the flow cytometry protocol to isolate parasite infected cells. Initial experiments used parasites expressing mCherry under the tubulin promoter. Cells were sorted form infected cultures (**a**) and infected IFN$\gamma^{-/-}$ mice (**b**). Gates used for sorting are shown in red. Sorted cells were imaged without further staining (cells from mice were stained with antibody to EPCAM prior to sorting). The images show merge DIC and fluorescence channels (Scale Bar= 1 µm). We constructed numerous strains using different fluorescent protein genes (mCherry, GFP, YFP, mRuby, mScarlet, tdNeon and tdTomato) to identify those that produce the brightest fluorescence and the most robust sorting differential. We find tdNeon and tdTomato to yield the strongest green and red fluorescence, respectively. This experiment was performed three times with similar results.

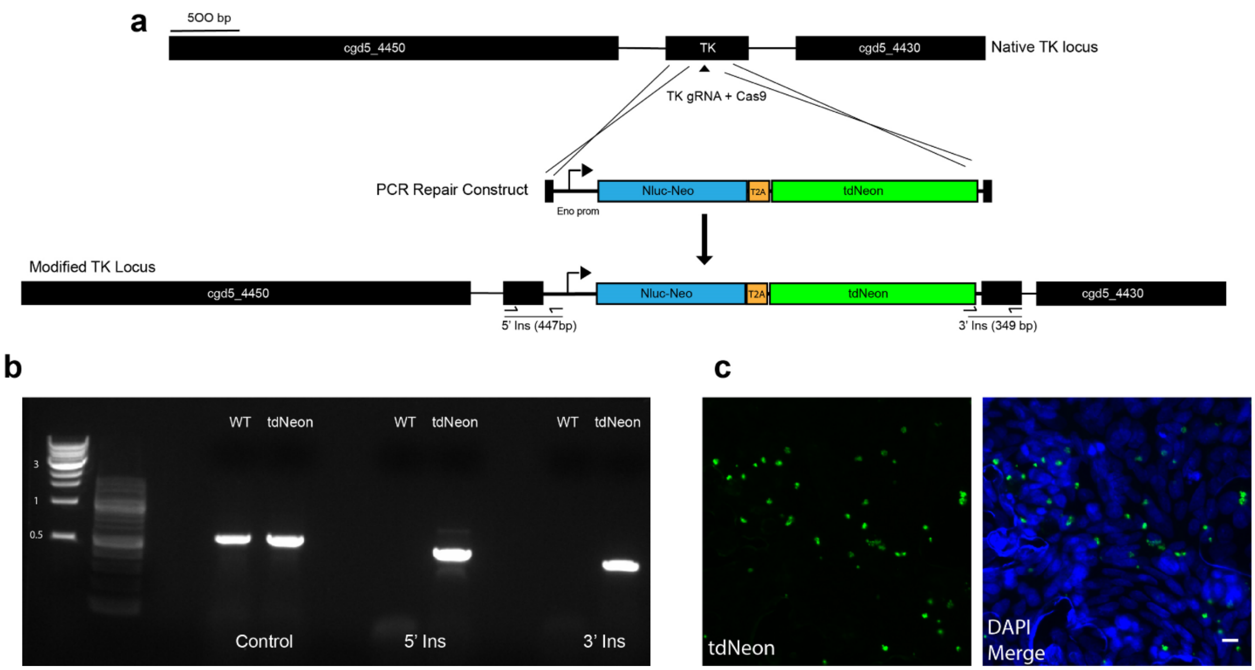

Supplementary Figure 6: Construction of eno tdNeon reporter strain. (a) The eno tdNeon constitutive reporter strain was constructed by linking the Nluc-Neo expression cassette with two copies of the mNeon Green fluorescent reporter gene in tandem. A viral T2A skip peptide linker was introduced between the tdNeon and Nluc-Neo cassettes. The expression construct was incorporated in the TK locus of the parasite and integration of the construct was validated by PCR **(b)**. **(c)** Expression of tdNeon reporter in the transgenic strain in a 24-hour infected culture (Scale Bar= 10 µm). This experiment was performed twice with similar results.

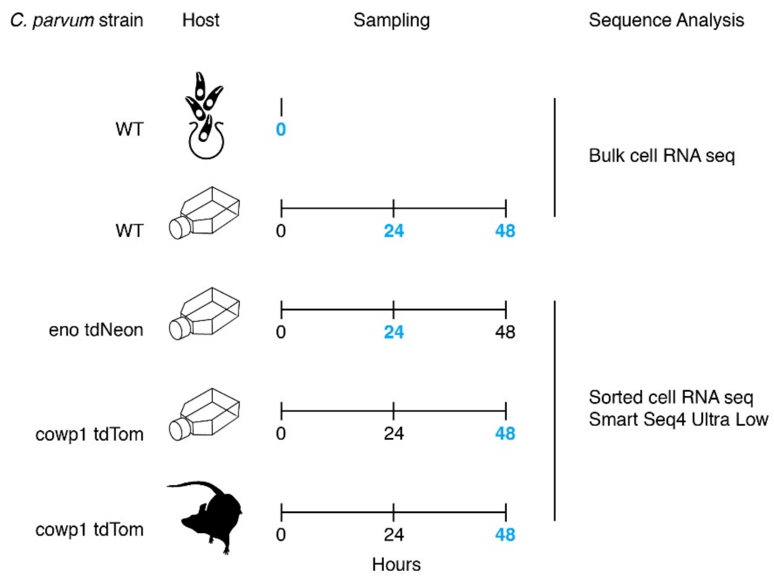

Supplementary Figure 7: Sampling of different parasite stages for RNA sequencing analysis. Schematic overview of RNA sequencing experiments conducted in this study indicating the *C. parvum* strain, the respective transgene, the source of parasites or infected cells (sporozoites freshly excysted from oocysts, infected HCT-8 cell cultures, or the resected small intestine of infected IFN$\gamma^{-/-}$ mice), the time point of parasite harvest following infection (in hours highlighted in blue), whether or not cells were subjected to fluorescence activated cell sorting.

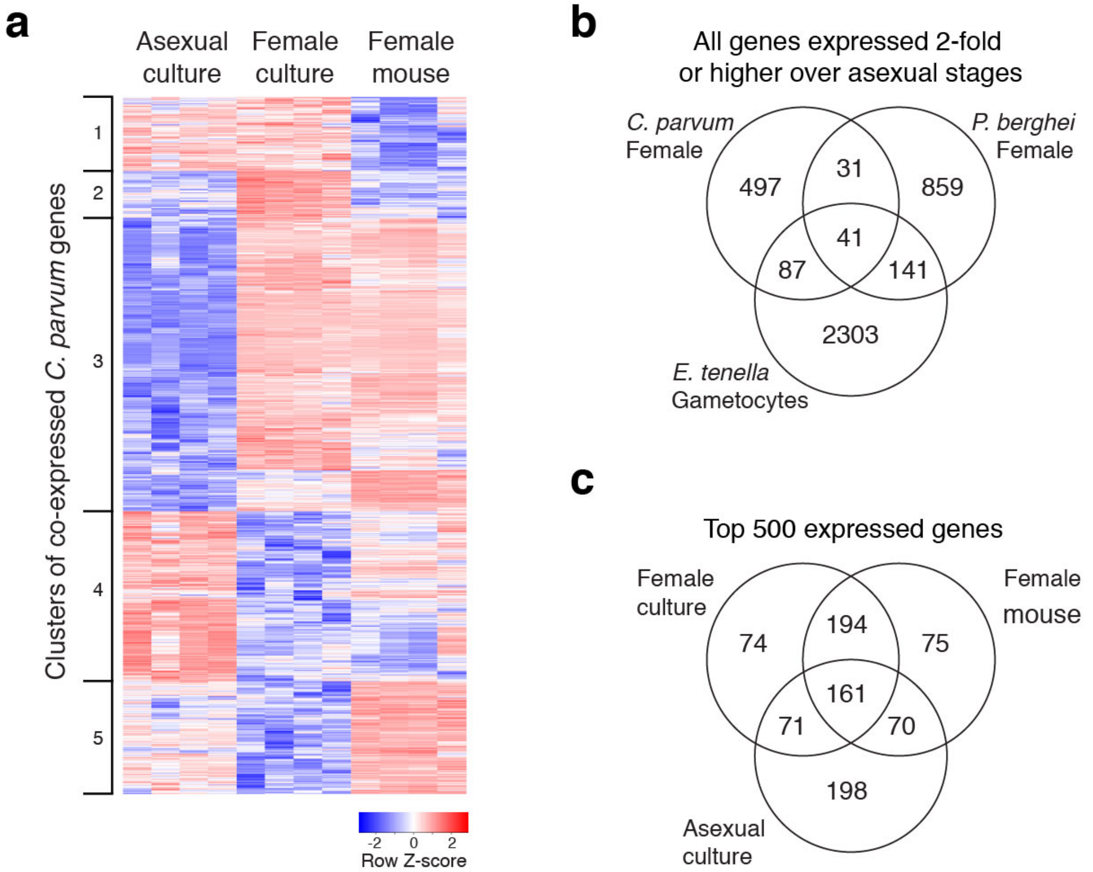

Supplementary Figure 8: Differential gene expression of asexual and female C. parvum sorted from HCT-8 cultures and infected mice. (**a**) A heatmap of cluster analysis of differentially expressed genes between the sorted cells (see Fig. 3a for further detail on sorted populations). Expression is given as a row z-score. Clusters are available for download as Supplementary File 3 (n= 4 biological replicates per group). (**b**) Venn diagram of all genes expressed 2-fold or higher in female (or in the case of *Eimeria tenella* gametocyte) stages over asexual stages. Numbers are given as ortholog groups containing one or more genes from the indicated species. See Supplementary File 4 for a complete list of ortholog groups and stage specific genes within each group for each species. (**c**) Venn diagram of the 500 most abundantly expressed genes for each parasite stage (n= 4 biological replicates per group). See Supplementary File 5 for complete lists of overlapping and unique genes.

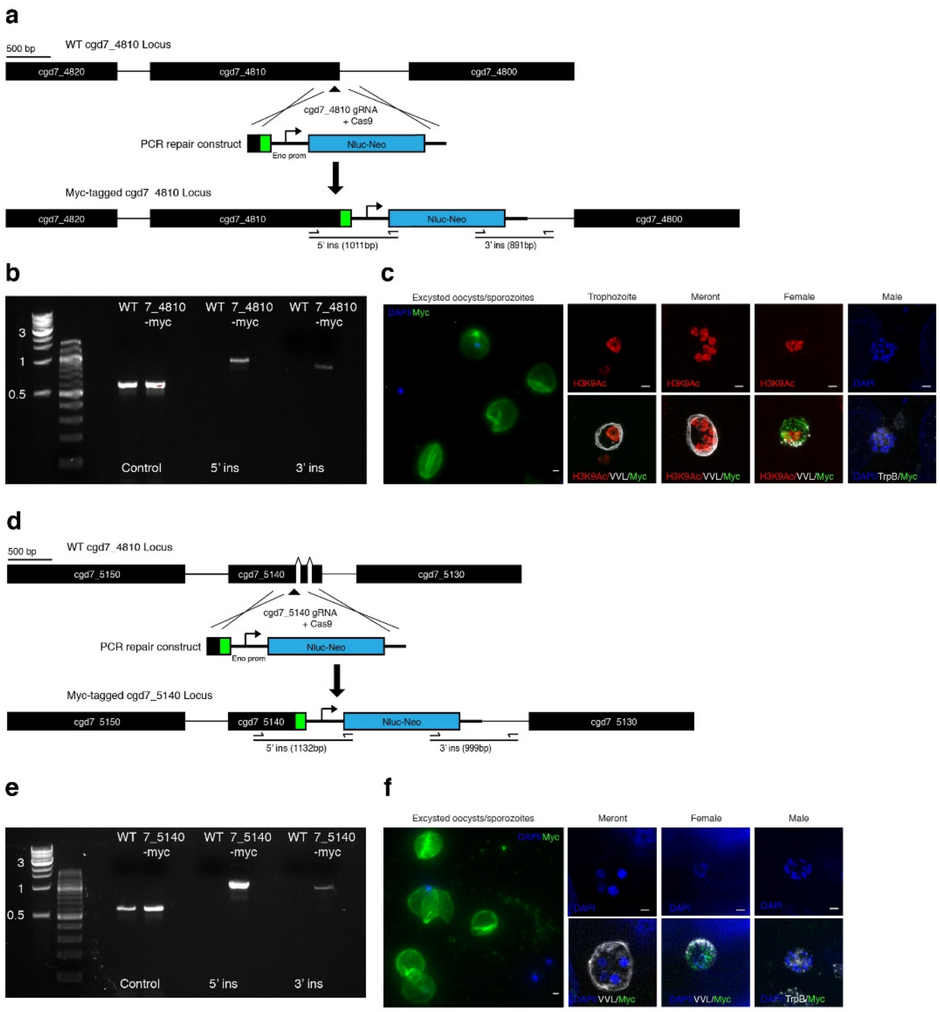

Supplementary Figure 9: Genetic validation of female specific gene identified by RNA sequencing analysis. We tested two genes highly expressed in females; cgd7_4810 and cgd7_5140. (**a, d**) Maps of the native loci, repair constructs, and modified loci for epitope tagging of cgd7_4810 (**a**) and cgd7_5140 (**d**). (**b, e**) PCR validation of integration and epitope tagging of cgd7_4810 (**b**) and cgd7_5140 (**e**), see (**a**) and (**d**) for expected sizes of the 5' and 3' integration amplicons. (**c & f,** Scale Bar = 1 µm) Immunofluorescence of excysted oocysts and infected HCT-8 cells with cgd7_4810-myc or cgd7_5140-myc parasites for 24 (trophozoite and meront) or 48 (female gamete and male gamont) hours of culture. Nuclei were visualized with an antibody to H3K9Ac or DAPI, and parasites were counterstained with VVL or TrpB. Both cgd7_4810 and cgd7_5140 expression is only observed in oocysts and females (Scale bars= 1 µm). Note that oocyst staining is only apparent after they are broken suggesting that these proteins are components of an inner layer of the wall. PCR mapping experiments were performed twice, and microscopy experiments were performed thrice.

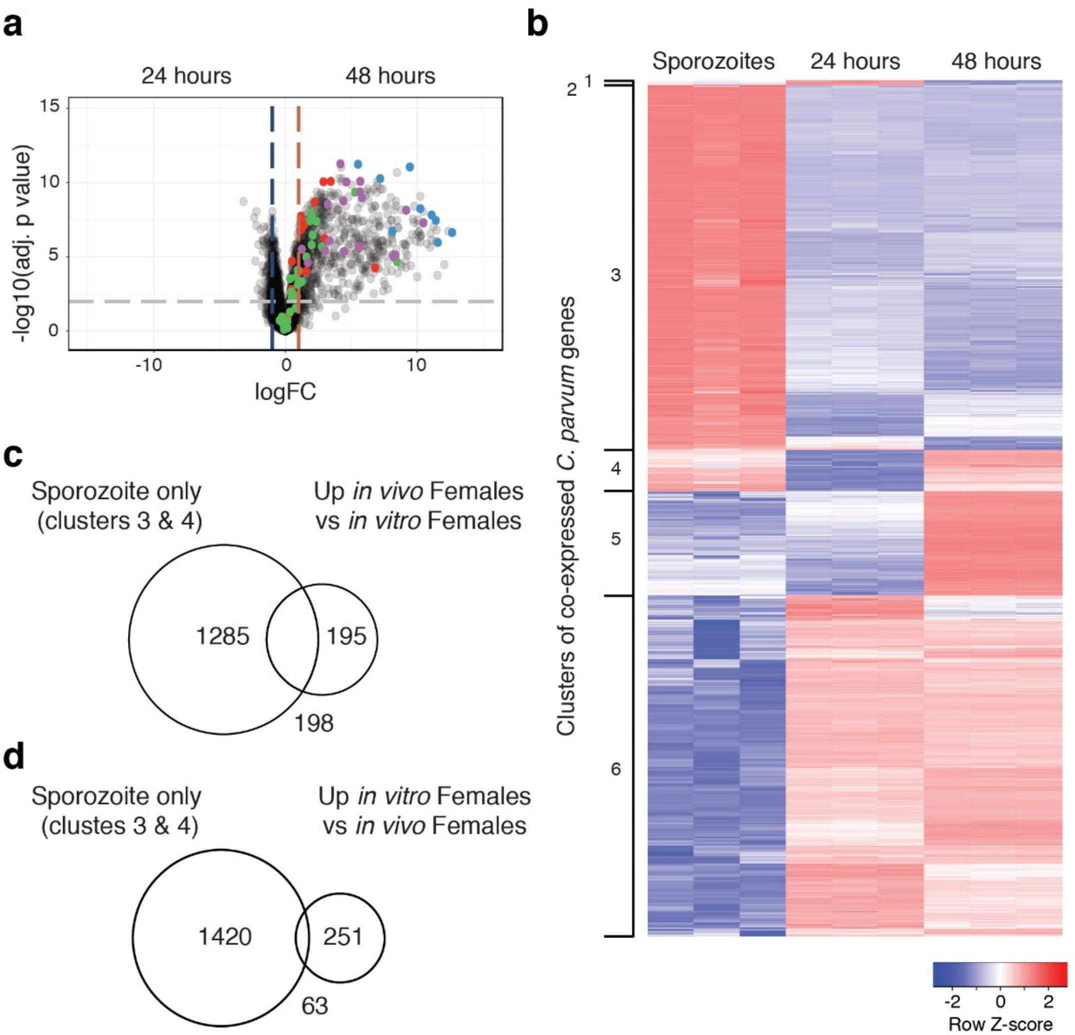

Supplementary Figure 10: Differential gene expression of excysted sporozoites and infected HCT-8 cultures. (**a**) Volcano plot of differentially expressed *C. parvum* genes between 24 and 48 hours in vitro (n=3 biological replicates per group). Each point represents a single *C. parvum* gene. The horizontal dashed line indicates an FDR of 0.01. Vertical dashed lines indicate log2 fold change of -1 and +1. Genes from the leading edge of gene enrichment analysis (Fig. 3b) are shown in red, carbohydrate (GO:0005975), green, DNA (GO:0006259), purple, redox (GO:0055114), and blue, oocyst wall proteome[23] dataset. (**b**) Heatmap and clustering analysis of *C. parvum* genes differentially expressed between sporozoites and infected HCT-8 cultures at 24- and 48-hours post infection (n= 3 biological replicates per group). Expression is given as row z-scores. (**c** & **d**) Venn diagrams comparing genes expressed by sporozoites (found in clusters 3 & 4 in (**c**) to genes expressed 2-fold or higher by in vivo females over in vitro females (**c**) or expressed 2-fold or higher by in vitro females over in vivo females (**d**). Complete list of shared genes in Supplementary File 6. Note that 40% of genes of this category in vivo females are also upregulated in sporozoites. No such enrichment is obvious in in vitro females.

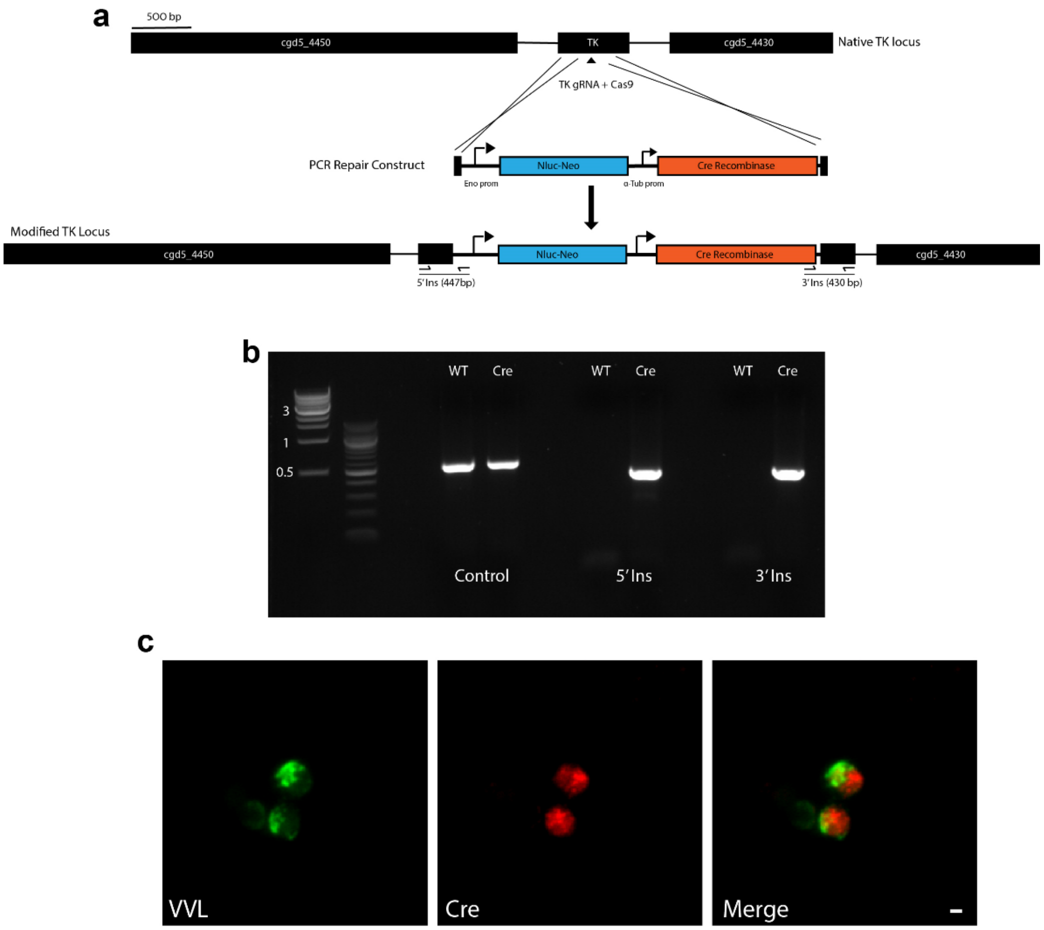

Supplementary Figure 11: Construction of a *C. parvum* strain expressing Cre recombinase. (**a**) Maps of PCR repair construct for Cre expression, TK locus & modified TK locus post insertion. (**b**) PCR analysis for integration of the Cre cassette in the TK locus. (**c**) IFA of the transgenic strain emerging from drug selection with antibody to Cre confirms expression of Cre recombinase. Parasites were counterstained with VVL (Scale Bar= 1 µm). PCR mapping experiment and microscopy experiments each were performed twice.

.

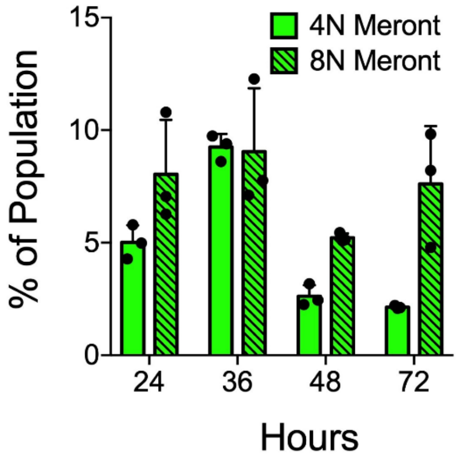

Supplementary Figure 12: Abundance of different meront types in culture over time. HCT-8 cell cultures were infected with H2b mNeon parasites and fixed at 24, 36, 48 and 72 hours. Cultures were scored for meronts with either eight (type I) and four (type II) nuclei which are represented here as % of all observed parasite stages. Three independent biological replicates were used for this experiment and the data is represented as mean ± SD. Note that we do not find a temporal association between the emergence of meronts with four nuclei and the (subsequent) emergence of sexual stages.

Supplementary Table 1: A list of primers used in this study. This table enlists primers that were used for making linear PCR repair product and for cloning plasmids.

Primer name	5'-3' Sequence
tkintgbbsf	gttggaagtaaatacttattagca
tkintgbbsr	aaactgctaataagtatttacttc
tkguide#4f	gttggaagaatacaatttctaagg
tkguide#4r	aaacccttagaaattgtattcttc
cgd6_2090g4858f	gttggttgtttcatatgacacaat
cgd6_2090g4858r	aaacattgtgtcatatgaaacaac
cgd8_2220g2bbsf	gttggagccaagaaagtttaagtca
cgd8_2220g2bbsr	aaactgacttaaactttcttggctc
enotkintf	tccagtactatgctatggtttgagaacagactttaagggaaatttatttgatggggaaactaaatatactgaaattcggt
tkinteno3r	tagcttttttgccacagcgacaaatagttttgatttcagtaagtttatcaccatagctgcgccaaattttgc
c13utrtkf	tccagtactatgctatggtttgagaacagactttaagggaaatttatttgaaagtagtttggccttttctagataatttt
eno3utrtkr	tagcttttttgccacagcgacaaatagttttgatttcagtaagtttatcaaattaagataaaaagaaaaacttaatcgatactatcctacac
cowp1 licf	gacaatacagaaaaaactagtacaggttgcgtcaagaaagttataacgacccctatcgtatcctacgagacgacgtgcatcggaccgacctgcaatgccgccaaaattggaagtggaggac gggaattc
cowp1 licr	agttaaaattctatcagattaaactatcaaagtagtttggccttttctagaattaagataaaaagaaaaacttaatcgatactatcctacacgcc
hap2licf3	gaactacgtaaaaataagaaaattgaaacaaggataattaataatagccaagagagcttgagccagggcatcaac
h2lic3'flankr2	tttttcagctaggcccatattaattaacttttaatatactttcattgcttcaattaagataaaaagaaaaacttaatcgatactatcctacacg
tk oe repairr	tagcttttttgccacagcgacaaatagttttgatttcagtaagtttatcatcatatttctatttaagttgcaatacgtatctg
eno3'utr-tag-r	aattaagataaaaagaaaaacttaatcgatactatcctacacgcc
atubprenof	tagtatcgattaagtttttctttttatcttaattacttgcagagataaagaaaaattcaatcaagaactta
atub5'utrr	gtttaacgaataactgtttaacgaataactttaac
h2baof	taaacagttattcgttaaacatggccccaaaaatgtcttcaaagaa
h2b1r	ggtggcgaccggtggatccttttgtcctccagtgaacttggtaa
ttmnh2b	aggatccaccggtcgccaccatggtttctaagggtgaagaagataacatgg
ttnmactr	actccaagaacaatattgatttacttgtataattcatccatacccataacatcagtgaaa
3' actin utr-f	atcaatattgttcttggagttgttcttaacagcttatttc
cplicbbf	aacagcttatttcctgaacacagatacgtattgcaacttaaatagaaatatgaactagtaccaatcagtttaaacgcgatg
creatubf	taaacagttattcgttaaacatgcccaagaagaagaggaagg
creatubr	actccaagaacaatattgatctagacagatctaaggccgcta
neo2ar	ccgcaagtcaacaatctacccctgccctcaccgaagaattcgtcaagaagacgatagaag
ttmneon2af	ggggtagattgttgacttgcggtgacgttgaggagaaccccggcccgatggtttctaagggtgaagaagata
ttmneon2ar	gctagccttgtataattcatccatacccataacatcagtg
crmnoen2af	atgaattatacaaggctagcgtgtccaagggcgaggaggacaac
crmneon2ar	aattttgccttaattaatcacttgtacagctcgtccatgcccatc
tkf	tgattaattaaggcaaaatttggcgcagctatggc
p2ar	aacgtcaccggcctgcttcaacaagctga
loxptub3utrf	gaagcaggccggtgacgttgaggagaaccccggcccgataacttcgtatagcatacattatacgaagttatagtgataattatccttgttcattgaattctctagatttaggaggtttggtttacc gc
loxptub3utrr	gcggtaaaccaaacctcctaaatctagagaattcaatgaacaaggataattatcactataacttcgtataatgtatgctatacgaagttatcgggccggggttctcctcaacgtcaccggcctgc ttc
loxptub3utr2f	taggaggtttggtttaccgcccggcgggctcgtgtagattagtatcttaatctatggaattttccctaataacttcgtatagcatacattatacgaagttatctgctacaaatttcagtttgcttaag caagccggagacgttgaagagaaccctggcccg
loxptub3utr2r	cgggccagggttctcttcaacgtctccggcttgcttaagcaaactgaaatttgtagcagataacttcgtataatgtatgctatacgaagttattagggaaaattccatagattaagatactaatct acacgagcccgccgggcggtaaaccaaacctccta
ttmng-2af	ttgaagagaaccctggcccggtttctaagggtgaagaagataacatggct
c1futr intr	taggtgtatcctcgaattattgtttttcc
cowp1intinsf	aacaaaaccagatagtagatgccca
creinsf	gctggaccaatgtaaatattgtcatg
crmneonintr	ttcaggttcagctcctcgtagc
h2binsf	atgcagtttctgagggtactaagg
hap2insr	tgaaaataatgaaatcgtattggtatccc
hape9insf	caataatttctgatgtagtagtgaatcaaact
tdneoninsf	tacctgaagaaccagccgatg
tdtominternal insf	acaccaagctggatatcacatcc
tkinsf	agcaatgaatgctggaaaatcaacg
tkinsr	ccgccttagaaattgtattcttcac
ttneonintinsr	accttgaccaaccatatcgaaatc
enointr	atgacgcaatatagaactaagtgtgtg
neorcr	ccgattctcaacgtatcgccttcta
6_2090dsr2	acttggcaaacgaaactcggagttg

Supplementary Files

Supplementary Figures & Tables: Supplementary Figures 1-12 and supplementary Table 1.

Supplementary File 1: Summary of sequencing information. Multi-tab excel file with information on total reads and reads mapped for each sample. Each tab contains the information for separate experiments.

Supplementary File 2: Differentially expressed genes. Multi-tab excel file containing the expression data for differentially expressed genes between sorted asexual and female infected cells in vitro and between female infected cells in vitro versus in vivo. Expression given as log2 transformed and normalized counts per million.

Supplementary File 3: Clusters of co-expressed genes. Multi-tab excel file containing expression data for each of the clusters of co-expressed genes outlined in Supplementary Figures 8a and 9b. Expression given as log2 transformed and normalized counts per million.

Supplementary File 4: Conservation of female or gametocyte expressed genes across three apicomplexan species. Multi-tab excel file containing genes that are shared or uniquely expressed between *C. parvum* females, *P. berghei* females, and *E. tenella* gametocytes as indicated in Supplementary Figure 8b.

Supplementary File 5: Shared expression of top 500 genes among sorted *C. parvum* stages. Multi-tab excel file containing overlapping or unique genes in the top 500 expressed genes in asexual parasites *in vitro*, female parasites *in vitro*, and female parasites *in vivo* as indicated in Supplementary Figure 8c.

Supplementary File 6: Shared expression of sporozoite genes. Multi-tab excel file containing genes expression by sporozoites compared to 24- and 48-hours post infection and their overlap with either in vitro or in vivo females as indicated in Supplementary Figure 10c and 10d.

Supplementary File 7: Supplementary code file.
