# Supplementary material for: Life cycle progression and sexual development of the apicomplexan parasite Cryptosporidium parvum
Source: Nat Microbiol. 2019 Sep 2;4(12):2226–36. doi: 10.1038/s41564-019-0539-x (PMC6877471; doi:10.1038/s41564-019-0539-x)
Supplement: Supplementary file 2 — Reporting Summary [file 41564_2019_539_MOESM2_ESM.pdf]

nature research

Corresponding author(s): Jayesh Tandel, Elizabeth English, Adam Sateriale, Jodi Gullicksrud, Daniel Beiting, Megan Sullivan, Brittain Pinkston, and Boris Striepen

Last updated by author(s): Jul 10, 2019

Reporting Summary

Nature Research wishes to improve the reproducibility of the work that we publish. This form provides structure for consistency and transparency in reporting. For further information on Nature Research policies, see Authors & Referees and the Editorial Policy Checklist.

Statistics

For all statistical analyses, confirm that the following items are present in the figure legend, table legend, main text, or Methods section.

n/a	Confirmed	
☐	☒	The exact sample size (*n*) for each experimental group/condition, given as a discrete number and unit of measurement
☐	☒	A statement on whether measurements were taken from distinct samples or whether the same sample was measured repeatedly
☐	☒	The statistical test(s) used AND whether they are one- or two-sided<br>*Only common tests should be described solely by name; describe more complex techniques in the Methods section.*
☒	☐	A description of all covariates tested
☒	☐	A description of any assumptions or corrections, such as tests of normality and adjustment for multiple comparisons
☐	☒	A full description of the statistical parameters including central tendency (e.g. means) or other basic estimates (e.g. regression coefficient) AND variation (e.g. standard deviation) or associated estimates of uncertainty (e.g. confidence intervals)
☐	☒	For null hypothesis testing, the test statistic (e.g. *F*, *t*, *r*) with confidence intervals, effect sizes, degrees of freedom and *P* value noted<br>*Give P values as exact values whenever suitable.*
☒	☐	For Bayesian analysis, information on the choice of priors and Markov chain Monte Carlo settings
☐	☒	For hierarchical and complex designs, identification of the appropriate level for tests and full reporting of outcomes
☒	☐	Estimates of effect sizes (e.g. Cohen's *d*, Pearson's *r*), indicating how they were calculated

Our web collection on statistics for biologists contains articles on many of the points above.

Software and code

Policy information about availability of computer code

Data collection	NIH image J 1.52a , ZEN 2.3 SP1 & GE Softworxs were used for image processing. RNA seq alignment was done using kallisto v0.44.0. R studio 1.1.463 was used for RNA seq analysis.
Data analysis	Morphometric data & parasite quantifications on parasite stages were collected by using particle area analysis & multi-point count tools on NIH Image J. Two-sided t-test was used for the analysis on GraphPad Prism 8. Transcripts were collapsed to gene symbols in R using Bioconductor tximport48. The tximport package and data were normalized using the TMM method (implemented in EdgeR). Counts were normalized using R package voom49, and then tested for differential abundance using limma v3.36.550 package. Additional analyses and graphical representations were performed using R stats, gplots, and ggplot2 packages. FlowJo v10 was used for the analysis of flow data. GraphPad Prsim 8 was used for statistical analysis. Gene Set Enrichment Analysis was carried out using GSEA software.

For manuscripts utilizing custom algorithms or software that are central to the research but not yet described in published literature, software must be made available to editors/reviewers. We strongly encourage code deposition in a community repository (e.g. GitHub). See the Nature Research guidelines for submitting code & software for further information.

Data

Policy information about availability of data

All manuscripts must include a data availability statement. This statement should provide the following information, where applicable:

- Accession codes, unique identifiers, or web links for publicly available datasets
- A list of figures that have associated raw data
- A description of any restrictions on data availability

RNA-seq data generated in this study are available through the GEO database repository under accession number GSE129267. Additional RNA-seq data that support the findings of this study are available through the NCBI Sequence Read Archive BioProject ID: PRJNA374918.

Field-specific reporting

Please select the one below that is the best fit for your research. If you are not sure, read the appropriate sections before making your selection.

☒ Life sciences ☐ Behavioural & social sciences ☐ Ecological, evolutionary & environmental sciences

For a reference copy of the document with all sections, see nature.com/documents/nr-reporting-summary-flat.pdf

Life sciences study design

All studies must disclose on these points even when the disclosure is negative.

Sample size	We did not have any data for different estimates of variability needed for power or sample size analysis. Instead we used standard sample size of three and observed statistical significance for experiments reported in the manuscript.
Data exclusions	None of the data points were excluded for the analysis.
Replication	Results of H2B-neon based stage-based kinetic experiments were reproduced by fine-time-point based kinetics experiments with strains tagged with sex-specific markers. Some of the genes identified RNA-seq analysis were validated by gene-tagging & IFA. In vivo experiments for post-fertilization stages were reproduced independently with H2B & COWP1-HA strains. Cre-lox experiments were reproduced by using orthogonal approaches like IFA & PCR. All the experiments were repeated at least 2 or 3 times with 3-4 biological replicates. The experimental results were reproduced independently. All the experiments were replicated with similar results.
Randomization	Mice used in these experiments were 4 weeks old females.
Blinding	All the experiments were performed without blinding. All of my experiments were exploratory based instead of hypothesis driven and we were not looking for phenotypes that would be result of gene perturbation. Hence we there was no need for blinding in my experiments.

Reporting for specific materials, systems and methods

We require information from authors about some types of materials, experimental systems and methods used in many studies. Here, indicate whether each material, system or method listed is relevant to your study. If you are not sure if a list item applies to your research, read the appropriate section before selecting a response.

Materials & experimental systems

n/a	Involved in the study	
☐	☒	Antibodies
☐	☒	Eukaryotic cell lines
☒	☐	Palaeontology
☐	☒	Animals and other organisms
☒	☐	Human research participants
☒	☐	Clinical data

Methods

n/a	Involved in the study	
☒	☐	ChIP-seq
☐	☒	Flow cytometry
☒	☐	MRI-based neuroimaging

Antibodies

Antibodies used	Rabbit Tryptophan synthase B (in house antibody, 1:750 dilution), Rabbit H3K9Ac (Millipore, Cat No-07-352, 1:1000 dilution), Mouse alpha-tubulin (Raised in-house antibody, 1:1000 dilution), Rat HA (Sigma Aldrich; Cat No- 11867423001. Clone 3F10, 1:300 dilution), Rabbit GFP (Abcam Cat. No- ab290, 1:1000 dilution), Rabbit Rad 51 (Millipore Cat No. ABE257, 1:300 dilution), Fluorescein labeled Vicia Villosa Lectin (Vector labs cat no- FL-1231, 1:1000 dilution), Biotinylated Maclura Pomifera Lectin (Vector Labs Cat No- B-1345, 1:1000 dilution), Biotinylated Vicia Villosa Lectin (Vector Labs Cat No. B-1235, 1:1000 dilution), Rabbit Cre (Novus Biologicals Cat No.- NB100-56133SS, 1:300 dilution), CD45.2 eFluor 450 (eBioscience Cat no 48-0454-82. Clone no 104, 1:1000 dilution), Anti-c-Myc Antibody (Cat No-MABE282. clone 9E10, 1:300 dilution) & EpCAM FITC, CD326 (EpCAM)-FITC (Miltenyi Biotec Order no- 130-102-995. Clone- caa7-9G8, 1:1000 dilution)
Validation	Tryptophan synthase B & tubulin antibodies have been validated in house by IFA or WB or both against the parasites or cell lines or both. Staining of these antibodies have been validated by us and demonstrated in the results. For commercial antibodies, please refer to manufacturer statements for corresponding antibodies on their websites.<br><br>1. Rabbit H3K9Ac Millipore, Cat No-07-352- https://www.emdmillipore.com/US/en/product/Anti-acetyl-Histone-H3-Lys9-Antibody,MM_NF-07-352<br>2. Rat HA Sigma Aldrich; Cat No- 11867423001. Clone 3F10- https://www.sigmaaldrich.com/catalog/product/roche/roahaha?lang=en&region=US<br>3. Rabbit GFP Abcam Cat. No- ab290, 1:1000 dilution- https://www.abcam.com/gfp-antibody-chip-grade-ab290.html<br>4. Rabbit Rad 51 Millipore Cat No. ABE257- https://www.emdmillipore.com/US/en/product/Anti-RAD51-Antibody,MM_NF-ABE257

5. Fluorescein labeled Vicia Villosa Lectin Vector labs cat no- FL-1231- https://vectorlabs.com/fluorescein-labeled-vicia-villosa-lectin-vvl-vva.html
6. Biotinylated Maclura Pomifera Lectin Vector Labs Cat No- B-1345- https://vectorlabs.com/biotinylated-maclura-pomifera-lectin-mpl.html
7. Rabbit Cre Novus Biologicals Cat No.- NB100-56133SS- https://www.novusbio.com/products/cre-antibody_nb100-56133
8. CD45.2 eFluor 450 eBioscience Cat no 48-0454-82. Clone no 104- https://www.thermofisher.com/antibody/product/CD45-2-Antibody-clone-104-Monoclonal/48-0454-82
9. Anti-c-Myc Antibody Cat No-MABE282. clone 9E10- https://www.sigmaaldrich.com/catalog/product/mm/mabe282?lang=en&region=US
10. EpCAM FITC, CD326 (EpCAM)-FITC Miltenyi Biotec Order no- 130-102-995- https://www.miltenyibiotec.com/US-en/products/macs-flow-cytometry/antibodies/primary-antibodies/cd326-epcam-antibodies-mouse-caa7-9g8-1-10.html

Eukaryotic cell lines

Policy information about cell lines

Cell line source(s)	HCT-8 colorectacl adenocarcinoma cell line was purchased from American Type Culture Collection.
Authentication	None of the authentication procedures were used. Cell line was directly purchased from ATCC.
Mycoplasma contamination	Cell lines were not tested for Mycoplasma contamination.
Commonly misidentified lines (See ICLAC register)	None according to the ICLAC register. HCT-8 cell line has been found to be the best host cells to infect C. parvum parasites.

Animals and other organisms

Policy information about studies involving animals; ARRIVE guidelines recommended for reporting animal research

Laboratory animals	IFN-gamma KO & Rag1 KO strains of Mus musculus were used. Female mice were used for all the experiments. Both male and femalemice were used to propagate strains. We used mice that are at least 4 weeks old. These mice strains were puchased from The Jackson Laboratory.
Wild animals	No wild animals were used.
Field-collected samples	No field samples were used.
Ethics oversight	Institutional Animal Care & Use Committee Office of Animal Welfare (IACUC), Penn Animal Welfare. University of Pennsylvania.

Note that full information on the approval of the study protocol must also be provided in the manuscript.

Flow Cytometry

Plots

Confirm that:

☒ The axis labels state the marker and fluorochrome used (e.g. CD4-FITC).

☒ The axis scales are clearly visible. Include numbers along axes only for bottom left plot of group (a 'group' is an analysis of identical markers).

☒ All plots are contour plots with outliers or pseudocolor plots.

☐ A numerical value for number of cells or percentage (with statistics) is provided.

Methodology

Sample preparation	For the in vitro sorting, cells were trypsinized, pelleted, passed through BD 40u filter & then resuspended in 400ul of FACS buffer for sorting, For in vivo samples, mice were killed 2 days post infection, the small intestine was resected, cut into small pieces and incubated in RPMI-1640 medium containing 10% FBS, 25mM HEPES, 5mM EDTA, 50µM β-mercaptoethanol and 0.145mg/ml dithiothrietol for 20 mins. The cell suspension was filtered through kitchen mesh, 70µ, and 40µ filters (BD Biosciences, San Jose, CA). Cells were pelleted, resuspended in buffer, stained with anti-CD45.2 antibody and sorted.
Instrument	BD FACSJazz sorter was used.
Software	FlowJo software was used for the data analysis.
Cell population abundance	The positive populations in eno-tdneon & cowp1 tdTomato samples are 60.6% (uninfected control-0.71%) & 18.3% (uninfected control-0.03%), respectively. The frequency of the positive population in cowp1 tdtomato infected mice samples is 0.12% (uninfected control- 0%). The positive populations have been validated by microscopy in preliminary experiments (Extended Data Figure. 5).

Gating strategy

For in vitro sorting, viable cells were selected by FSC v/s SSC sorts. Then positive cells were sorted by SSC v/s tdTomato or mNeon sorts. For in vivo sorts, viable cells were selected by FSC v/s SSC sorts. Population that was negative for both Aqua Live Dead (viability stain) & CD45.2 e450 (lymphocytes) was then selected. Positive population was selected by SSC v/s tdTomato sorts.

☐ Tick this box to confirm that a figure exemplifying the gating strategy is provided in the Supplementary Information.
