# Supplementary material for: Life cycle progression and sexual development of the apicomplexan parasite Cryptosporidium parvum
Source: Nat Microbiol. 2019 Sep 2;4(12):2226–36. doi: 10.1038/s41564-019-0539-x (PMC6877471; doi:10.1038/s41564-019-0539-x)
Supplement: Supplementary file 9 — Supplementary Code. [file 41564_2019_539_MOESM9_ESM.pdf]

Supplemental code file for manuscript titled: Lifecycle Progression and Sexual Development of the Apicomplexan Parasite *Cryptosporidium parvum*

Jayesh Tandel, Elizabeth English, Adam Sateriale, Jodi Gullicksrud, Daniel P. Beiting, Megan Sullivan, Brittain Pinkston, and Boris Striepen

produced on 2019-06-03

Contents

1 Introduction **2**

2 Background **2**

3 Reproducibility and accessibility **2**

4 R packages used for this analysis **3**

5 Processing raw reads **3**
- 5.1 QC of raw reads with fastqc . . . 3
- 5.2 Pseudoalignment of raw reads with Kallisto . . . 3
- 5.3 summarizing QC with multiqc . . . 4

6 Using R/bioconductor to import and analyze RNAseq data **4**
- 6.1 Annotation . . . 4
- 6.2 Sample info . . . 4

7 Identification of a female-specific transcriptional program in *C. parvum.* **5**
- 7.1 filtering and normalization . . . 5
- 7.2 PCA of data after filtering and normalization . . . 7
- 7.3 Volcano plot: FACS sorted females vs asexual stage (in vitro) . . . 8
- 7.4 Volcano plot: females sorted from culture vs mice . . . 10
- 7.5 Heatmap: identification of co-expression modules . . . 11

8 Functional annotation and enrichment analysis **13**
- 8.1 signatures for GSEA analysis . . . 13
- 8.2 GSEA using CAMERA . . . 15
- 8.3 reading in leading edge genes from GSEA analysis . . . 16
- 8.4 Volcano plot: females vs asexual stage from culture - Figure 3E . . . 16
- 8.5 Volcano plot: females sorted from culture versus mouse infection - Figure 3F . . . 18
- 8.6 heatmap: gliding machinery - Figure 3G . . . 19

9 Identification of the sexual differentiation program in *C. parvum.* **20**
- 9.1 filtering and normalization . . . 20
- 9.2 PCA of data after filtering and normalization . . . 22
- 9.3 Volcano plot: sexual development in bulk culture - Supp. Fig 8A . . . 23
- 9.4 Volcano plot: asexual growth . . . 25
- 9.5 Heatmap: identification of co-expression modules - Supp. Fig 8B . . . 26

10 Function grouping of genes **28**
- 10.1 heatmap: meiosis and DNA repair - Figure 4A . . . 28

10.2 heatmaps: oocyst environmental resilience - Figure 4B . . . 29
10.2.1 oocyst wall proteins . . . 29
10.2.2 oxidoreductases . . . 31
10.2.3 proteases . . . 32
10.2.4 glycosylation . . . 34
10.2.5 Polysaccharide pyruvyl transferases heatmap . . . 35
10.2.6 Fatty Acid PKS heatmap . . . 36
10.3 heatmap: energy storage - Figure 4C . . . 37
10.4 heatmap: AP2 and AP2-related genes - Figure 4D . . . 38

11 Global analysis that incorporates all samples across all experiments 40
11.1 PCA showing batch effect . . . 41
11.2 PCA after correcting for batch effect - Figure 3D . . . 42
11.3 Creating DGEList from batch corrected counts . . . 43

12 Session info 44

1 Introduction

This reproducible and dynamic report was created using Rmarkdown and the Knitr package, and summarizes the basic code and outputs (plots, tables, etc) produced during the course. The relative file paths indicated in the code below assume that your project working directly is structured as indicated **here**

---

2 Background

The apicomplexan parasite *Cryptosporidium* is a leading global cause of severe diarrheal disease and an important contributor to early childhood mortality. Currently there are no fully effective treatments or vaccines available. Transmission of the disease occurs through ingestion of oocysts, through direct contact or contaminated water or food. Oocysts are meiotic spores and the product of parasite sex. *Cryptosporidium* has a single host lifecycle where both asexual and sexual processes unfold in the intestine of infected hosts. Here we use the new-found ability to genetically engineer *Cryptosporidium* to make life cycle progression and parasite sex tractable. We derive reporter strains to follow parasite development in culture and infected mice and define the genes that orchestrate sex and oocyst formation through mRNA sequencing of sorted cells. After two days, parasites in cell culture show pronounced sexualization, but productive fertilization does not occur and infection falters. In contrast in infected mice, male gametes successfully fertilize females, leading to meiotic division and sporulation. To rigorously test for fertilization, we devised a two-component genetic crossing assay employing a Cre recombinase activated reporter. Our findings suggest obligate developmental progression towards sex in *Cryptosporidium*, which has important implications for the treatment and prevention of the infection.

The code below shows how raw data was preprocessed, mapped, and analyzed to identify stage-specific gene expression

---

3 Reproducibility and accessibility

In order to reproduce *all* steps listed below, including QC of raw reads and read mapping, raw fastq files will need to be downloaded from the Gene Expression Omnibus, under accession GSE129267. Prealigned data and all code used in this analysis, including the Rmarkdown document used to compile this supplementary code file, are all available on GitHub **here**. Once this GitHub repo has been downloaded, navigate to `/CryptoSex_manuscript/ANALYSIS/code` to find the Rmarkdown document as well as an RProject file. This

should be your working directory for executing code. Downloaded data should be placed in a new directory: `/CryptoSex_manuscript/DATA/raw`

---

4 R packages used for this analysis

A variety of R packages was used for this analysis. All graphics and data wrangling were handled using the tidyverse suite of packages. All packages used are available from the Comprehensive R Archive Network (CRAN), Bioconductor.org, or Github.

```
library(tidyverse)
library(reshape2)
library(tximport)
library(RColorBrewer)
library(genefilter)
library(edgeR)
library(matrixStats)
library(gplots)
library(limma)
library(gt)
library(cowplot)
library(WGCNA)
```

---

5 Processing raw reads

5.1 QC of raw reads with fastqc

Quality control of raw reads was carried out using fastqc.

```
# threads option (-t below) may need to be adjusted for your machine.
fastqc ../../DATA/raw/*.gz -t 24 -o /CryptoSex_manuscript/QA/fastqc
```

5.2 Pseudoalignment of raw reads with Kallisto

Raw reads were mapped to the *Cryptosporidium parvum* reference transcriptome available on Ensembl here using Kallisto, version 0.45. The quality of raw reads, as well as the results of Kallisto mapping are summarized using multiqc. The resulting multiqc report can be found in the github project repo in the `/CryptoSex_manuscript/QA/` directory. *Note*: due to size limitation, neither the reference fastq file nor the indexed fastq could be stored in the GitHub repo.

```
# build index from reference fasta from Ensembl C. parvum Iowa II transcriptome
kallisto index -i CryptoIndex Cryptosporidium_parvum_iowa_ii.ASM16534v1.cdna.all.fa

# use Kallisto to map reads to the indexed reference transcriptome
kallisto quant -i CryptoIndex -o female_invitro1 -t 24 -b 60 --single -l 500 -s 100 Female_sort_invitro[illegible]
kallisto quant -i CryptoIndex -o female_invitro2 -t 24 -b 60 --single -l 500 -s 100 Female_sort_invitro[illegible]
kallisto quant -i CryptoIndex -o female_invitro3 -t 24 -b 60 --single -l 500 -s 100 Female_sort_invitro[illegible]
kallisto quant -i CryptoIndex -o female_invitro4 -t 24 -b 60 --single -l 500 -s 100 Female_sort_invitro[illegible]

kallisto quant -i CryptoIndex -o asexual_invitro1 -t 24 -b 60 --single -l 500 -s 100 Asexual_sort_invit[illegible]
kallisto quant -i CryptoIndex -o asexual_invitro2 -t 24 -b 60 --single -l 500 -s 100 Asexual_sort_invit[illegible]
kallisto quant -i CryptoIndex -o asexual_invitro3 -t 24 -b 60 --single -l 500 -s 100 Asexual_sort_invit[illegible]
```

```
kallisto quant -i CryptoIndex -o asexual_invitro4 -t 24 -b 60 --single -l 500 -s 100 Asexual_sort_invit

kallisto quant -i CryptoIndex -o female_invivo1 -t 24 -b 60 --single -l 500 -s 100 Female_sort_invivo1_
kallisto quant -i CryptoIndex -o female_invivo2 -t 24 -b 60 --single -l 500 -s 100 Female_sort_invivo2_
kallisto quant -i CryptoIndex -o female_invivo3 -t 24 -b 60 --single -l 500 -s 100 Female_sort_invivo3_
kallisto quant -i CryptoIndex -o female_invivo4 -t 24 -b 60 --single -l 500 -s 100 Female_sort_invivo4_

kallisto quant -i CryptoIndex -o crypto_24hr_rep1 -t 24 -b 60 24hr-1-RNeasy_S1_mergedLanes_R1.fastq.gz 
kallisto quant -i CryptoIndex -o crypto_24hr_rep2 -t 24 -b 60 24hr-2-RNeasy_S2_mergedLanes_R1.fastq.gz 
kallisto quant -i CryptoIndex -o crypto_24hr_rep3 -t 24 -b 60 24hr-3-RNeasy_S3_mergedLanes_R1.fastq.gz 

kallisto quant -i CryptoIndex -o crypto_48hr_rep1 -t 24 -b 60 48hr-1-RNeasy_S4_mergedLanes_R1.fastq.gz 
kallisto quant -i CryptoIndex -o crypto_48hr_rep2 -t 24 -b 60 48hr-2-RNeasy_S5_mergedLanes_R1.fastq.gz 
kallisto quant -i CryptoIndex -o crypto_48hr_rep3 -t 24 -b 60 48hr-3-RNeasy_S6_mergedLanes_R1.fastq.gz 

kallisto quant -i CryptoIndex -o crypto_sporo_rep1  -t 24 -b 60 --single -l 250 -s 30 Sporo-mRNA_S4_mer
kallisto quant -i CryptoIndex -o crypto_sporo_rep2  -t 24 -b 60 --single -l 250 -s 30 Sporo-mRNA_S5_mer
kallisto quant -i CryptoIndex -o crypto_sporo_rep3  -t 24 -b 60 --single -l 250 -s 30 Sporo-mRNA_S6_mer
```

5.3 summarizing QC with multiqc

```
#move kallisto log files into same folder with fastqc outputs so all are in the same directory for mult
multiqc -d /CryptoSex_manuscript/QA/fastqc
#move the resulting multiqc report into the parent QA folder in your project directory
```

---

6 Using R/bioconductor to import and analyze RNAseq data

After read mapping with Kallisto, TxImport was used to read kallisto outputs into the R environment. Annotation data from Ensembl was used to 'collapse' data from transcript-level to gene-level.

6.1 Annotation

Annotation data for *Cryptosporidium parvum* Iowa II strain retrieved from ensemble here

```
cTx <-read_tsv("Cryptosporidium_parvum_iowa_ii.ASM16534v1.37.ena.tsv")
cTx <- dplyr::rename(cTx, target_id = transcript_stable_id)
cTx <- dplyr::rename(cTx, gene_name = gene_stable_id)
cTx <- cTx[,c(4,3)]
```

6.2 Sample info

```
# read in study design file
targets <-read_tsv("StudyDesign.txt")
# set file paths to your kallisto output folders that contain quantification data
cfiles <- file.path("../readMapping", targets$sample, "abundance.h5")

# use TxImport package to read Kallisto data into R
Txi_gene <-tximport(cfiles,
                    type = "kallisto",
                    tx2gene = cTx,
                    txOut = FALSE, #false collapses transcripts to genes
```

```
                    countsFromAbundance = "lengthScaledTPM")

# save the resulting R data object for later use
save(Txi_gene, file = "Txi_gene")

# capture essential variables of interest from the study design
sex <- as.factor(targets$sex_stage)
origin <- as.factor(targets$origin)
rep <- as.factor(targets$rep)
group <- as.factor(paste(targets$sex_stage, targets$origin, sep = "_"))
batch <- as.factor(targets$exper)

# capture sample labels for later use
SampleLabels <- targets$sample

# use gt package to produce table of study design
gt(targets)
```

sample	sex_stage	origin	rep	exper	batch	host
female_invitro1	female	invitro	1	2	2	human
female_invitro2	female	invitro	2	2	2	human
female_invitro3	female	invitro	3	2	2	human
female_invitro4	female	invitro	4	2	2	human
female_invivo1	female	invivo	1	3	2	mouse
female_invivo2	female	invivo	2	3	2	mouse
female_invivo3	female	invivo	3	3	2	mouse
female_invivo4	female	invivo	4	3	2	mouse
asexual_invitro1	asexual	invitro	1	2	2	human
asexual_invitro2	asexual	invitro	2	2	2	human
asexual_invitro3	asexual	invitro	3	2	2	human
asexual_invitro4	asexual	invitro	4	2	2	human
crypto_sporo_rep1	sporozoite	invivo	1	1	1	NA
crypto_sporo_rep2	sporozoite	invivo	2	1	1	NA
crypto_sporo_rep3	sporozoite	invivo	3	1	1	NA
crypto_24hr_rep1	asexual	invitro	1	1	1	human
crypto_24hr_rep2	asexual	invitro	2	1	1	human
crypto_24hr_rep3	asexual	invitro	3	1	1	human
crypto_48hr_rep1	sexual	invitro	1	1	1	human
crypto_48hr_rep2	sexual	invitro	2	1	1	human
crypto_48hr_rep3	sexual	invitro	3	1	1	human

---

7 Identification of a female-specific transcriptional program in *C. parvum.*

7.1 filtering and normalization

```
load("Txi_gene")

# taking only the first 12 samples in the dataset,
```

```
# which correspond to batches 2 and 3
Txi_gene <- Txi_gene$counts[,1:12]
# use EdgeR create DGEList object from counts
myDGEList <- DGEList(Txi_gene)
# use the 'cpm' function from EdgeR to get counts per million
log2.cpm <- cpm(myDGEList, log=TRUE)
log2.cpm.df <- as_tibble(log2.cpm)
colnames(log2.cpm.df) <- SampleLabels[1:12]
log2.cpm.df <- melt(log2.cpm.df)
colnames(log2.cpm.df) <- c("sample", "expression")

# plot of signal distribution for raw data
p1 <- ggplot(log2.cpm.df, aes(x=sample, y=expression, fill=sample)) +
  geom_violin(trim = FALSE, show.legend = FALSE) +
  stat_summary(fun.y = "median",
               geom = "point",
               shape = 124, size = 6,
               color = "black",
               show.legend = FALSE) +
  labs(y="log2 expression", x = "sample",
       title="raw data") +
  coord_flip() +
  theme_bw()

# filtering to keep only genes that had > 10 cpm in at least 4 samples
cpm <- cpm(myDGEList)
keepers <- rowSums(cpm>10)>=4
myDGEList.filtered <- myDGEList[keepers,]
# normalize using TMM method from calnormfactors function in EdgeR package
myDGEList.filtered.norm <- calcNormFactors(myDGEList.filtered, method = "TMM")
log2.cpm.filtered.norm <- cpm(myDGEList.filtered.norm, log=TRUE)
log2.cpm.filtered.norm.df <- as_tibble(log2.cpm.filtered.norm)
colnames(log2.cpm.filtered.norm.df) <- SampleLabels[1:12]
log2.cpm.filtered.norm.df <- melt(log2.cpm.filtered.norm.df)
colnames(log2.cpm.filtered.norm.df) <- c("sample", "expression")

normData <- as_tibble(log2.cpm.filtered.norm, rownames = "geneSymbol")
colnames(normData) <- c("geneSymbol", SampleLabels[1:12])
write_tsv(normData, "normData.txt")

# plot of signal distribution again to see effect of filtering an normalization
p2 <- ggplot(log2.cpm.filtered.norm.df, aes(x=sample, y=expression, fill=sample)) +
  geom_violin(trim = FALSE, show.legend = FALSE) +
  stat_summary(fun.y = "median",
               geom = "point",
               shape = 124, size = 6,
               color = "black",
               show.legend = FALSE) +
  labs(y="log2 expression", x = "sample",
       title="filtered, normalized data") +
  coord_flip() +
  theme_bw()
```

```
plot_grid(p1, p2, labels = c("A", "B"))
```

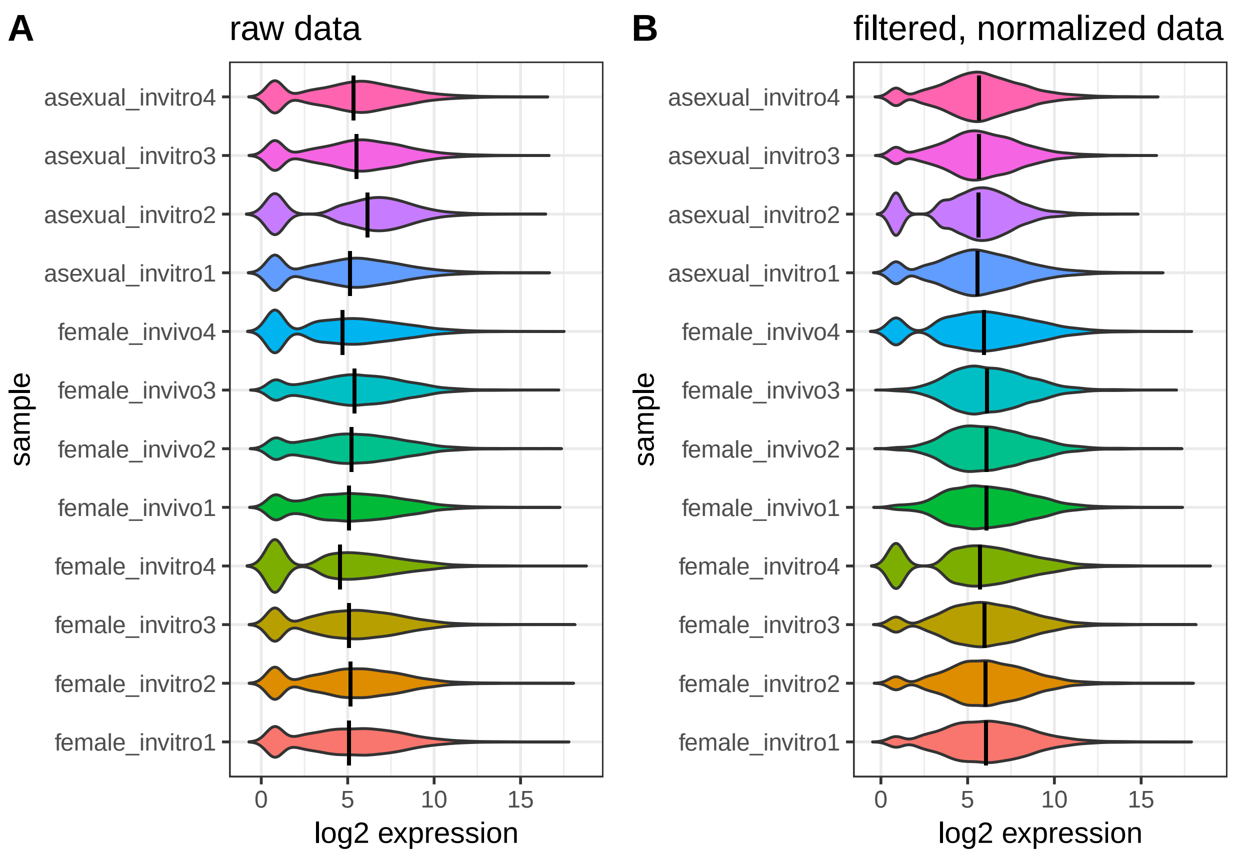

Filtering and normalization were carried out to improve our ability to detect differentially expressed genes. For filtering, only genes with >= 10 counts per million (CPM) in at least 4 or more samples kept. This reduced the number of genes from **3805** to **3099**. In addition, the TMM method was used for between-sample normalization.

7.2 PCA of data after filtering and normalization

Principal Component Analysis (PCA) plots reduce complex datasets to a 2D representation where each axis represents a source of variance (known or unknown) in the dataset. As you can see from the plots below, Principal Component 1 (PC1; X-axis), which accounts for >53% of the variance in the data, is separating the samples based on sex. PC2 (Y-axis) accounts for a much smaller source of variance (~18%) and can be attributed to variation between females recovered from culture versus versus mice.

```
# running PCA
pca.res <- prcomp(t(log2.cpm.filtered.norm), scale.=F, retx=T)
pc.var<-pca.res$sdev^2
pc.per<-round(pc.var/sum(pc.var)*100, 1)

# converting PCA result into a tibble for plotting
pca.res.df <- as_tibble(pca.res$x)
# plotting PCA
ggplot(pca.res.df, aes(x=PC1, y=PC2, color=sex[1:12], shape=origin[1:12])) +
  geom_point(size=4) +
```

```
  theme(legend.position="right") +
  xlab(paste0("PC1 (",pc.per[1],"%",")")) +
  ylab(paste0("PC2 (",pc.per[2],"%",")")) +
  labs(title="PCA of sort-purified female and asexual stage C. parvum",
       subtitle = "Principal component analysis (PCA) showing clear separation \nbetween females and ase
       color = "sex", shape="origin") +
  theme_bw() +
  theme(plot.title = element_text(face="bold"))
```

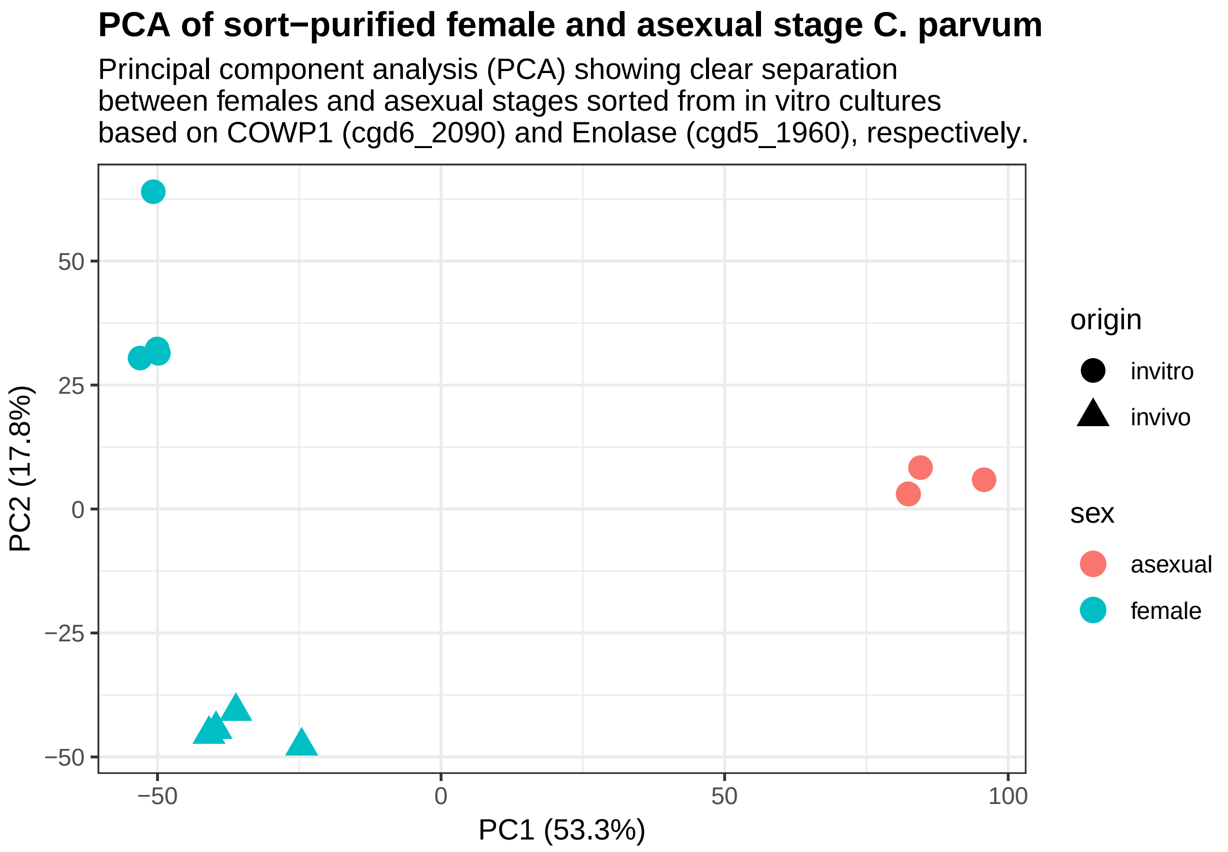

7.3 Volcano plot: FACS sorted females vs asexual stage (in vitro)

Volcano plots are convenient ways to represnt gene expression data because they combine magnitude of change (X-axis) with significance (Y-axis). Since the Y-axis is the inverse log10 of the adjusted Pvalue, higher points are more significant. In the case of this particular plot, there are many genes in the upper right of the plot, which represent genes that are significantly **higher** in females, compared to asexual stages.

```
# setting up model matrix without an intercept
design <- model.matrix(~0 + group[1:12, drop = TRUE])
colnames(design) <- levels(group[1:12, drop = TRUE])

# using VOOM function from Limma package to apply precision weights to each gene
v.myDGEList.filtered.norm <- voom(myDGEList.filtered.norm, design, plot = FALSE)
fit <- lmFit(v.myDGEList.filtered.norm, design)

# setting up contrast matrix for two main pairwise comparisons
```

```
contrast.matrix <- makeContrasts(female.vs.asexual_invitro = female_invitro - asexual_invitro,
                                 female_invivo.vs.invitro = female_invivo - female_invitro,
                                 levels=design)

fits <- contrasts.fit(fit, contrast.matrix)
# extracting stats
ebFit <- eBayes(fits)
# listing stats for all genes in the dataset to be used for making volcano plot
myTopHits1 <- topTable(ebFit, adjust ="BH", coef=1, number=10000, sort.by="logFC")
myTopHits1 <- as_tibble(myTopHits1, rownames = "geneSymbol")
# volcano plot
ggplot(myTopHits1, aes(y=-log10(adj.P.Val), x=logFC, text = paste("Symbol:", geneSymbol))) +
  geom_point(size=2) +
  ylim(-0.5,15) +
  xlim(-15,15) +
  geom_hline(yintercept = -log10(0.01), linetype="longdash", colour="grey", size=1) +
  geom_vline(xintercept = 1, linetype="longdash", colour="#BE684D", size=1) +
  geom_vline(xintercept = -1, linetype="longdash", colour="#2C467A", size=1) +
  labs(title="females vs. asexual stages sorted from culture",
       subtitle = "Volcano plot comparing expression of genes between females and asexual stages \nsorte
  theme_bw() +
  theme(axis.text=element_text(size=16),
        axis.title=element_text(size=18),
        plot.title = element_text(face="bold"),
        panel.border = element_rect(colour = "black", fill=NA, size=1))
```

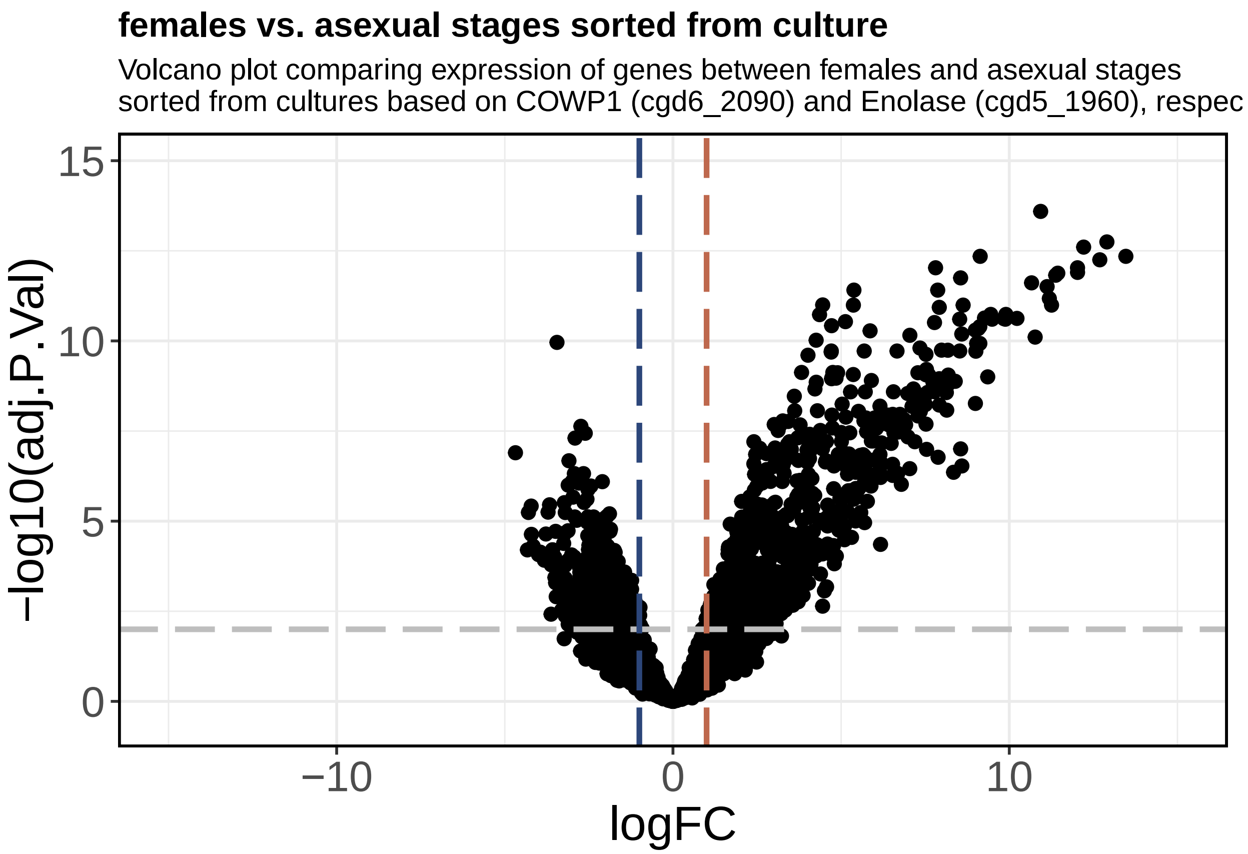

7.4 Volcano plot: females sorted from culture vs mice

Since our PCA above showed a small amount of variance that is accounted for based on whether females were sorted from culture compared to those sorted from infected mice, we now view this comparison using a volcano plot as well.

```
# looking at the second coefficient from our contrast matrix corresponding to females from mice vs cult
myTopHits2 <- topTable(ebFit, adjust ="BH", coef=2, number=10000, sort.by="logFC")
myTopHits2 <- as_tibble(myTopHits2, rownames = "geneSymbol")
ggplot(myTopHits2, aes(y=-log10(adj.P.Val), x=logFC, text = paste("Symbol:", geneSymbol))) +
  geom_point(size=2) +
  ylim(-0.5,15) +
  xlim(-15,15) +
  geom_hline(yintercept = -log10(0.01), linetype="longdash", colour="grey", size=1) +
  geom_vline(xintercept = 1, linetype="longdash", colour="#BE684D", size=1) +
  geom_vline(xintercept = -1, linetype="longdash", colour="#2C467A", size=1) +
  labs(title="Females from infected mice vs culture",
       subtitle = "Volcano plot comparing expression of genes between females \nsorted from in infected
  theme_bw() +
  theme(axis.text=element_text(size=16),
        axis.title=element_text(size=18),
        plot.title = element_text(face="bold"),
        panel.border = element_rect(colour = "black", fill=NA, size=1))
```

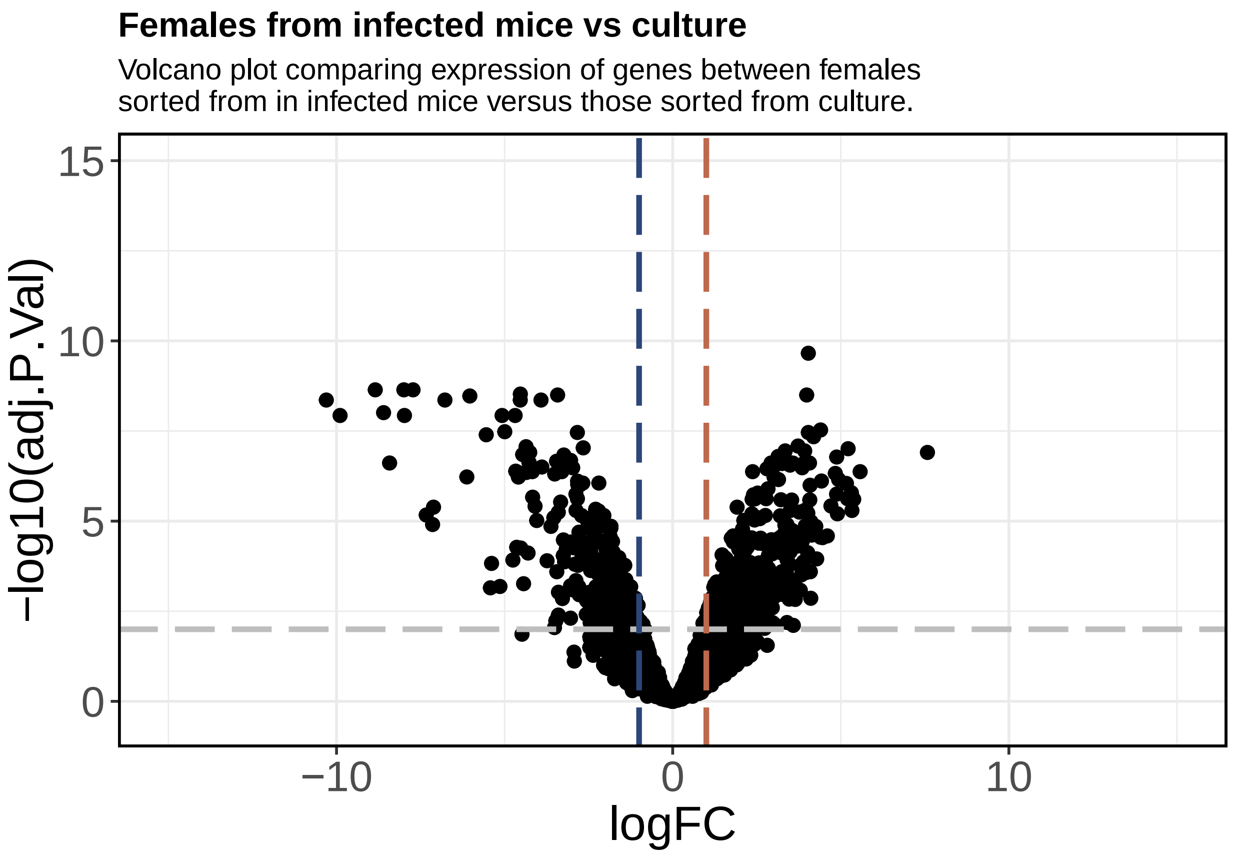

7.5 Heatmap: identification of co-expression modules

```
colnames(v.myDGEList.filtered.norm$E) <- SampleLabels[1:12]
# using decideTests to identify DEGs based on FDR and logFC
results <- decideTests(ebFit, method="global", adjust.method="BH", p.value=0.01, lfc=1)
# pulling these genes out along with their expression data
DiffGenes <- v.myDGEList.filtered.norm$E[results[,1] !=0 | results[,2] !=0,]
# setting color palette for all heatmaps moving forward
myheatcol <-colorRampPalette(colors=c("blue","white", "red"))(100)
clustRows <- hclust(as.dist(1-cor(t(DiffGenes), method="pearson")), method="complete")
clustColumns <- hclust(as.dist(1-cor(DiffGenes, method="spearman")), method="complete")
clust.assign <- cutree(clustRows, k=5)

module.color <- rainbow(length(unique(clust.assign)), start=0.1, end=0.9)
module.color <- module.color[as.vector(clust.assign)]

#construct a table of the DEGs with stats for saving
DiffGenes.table <- as_tibble(DiffGenes, rownames = "geneSymbol")
DiffGenes.table <- DiffGenes.table %>%
  dplyr::left_join(myTopHits1, by="geneSymbol") %>%
  dplyr::select(geneSymbol:logFC, adj.P.Val) %>%
  dplyr::left_join(myTopHits2, by="geneSymbol") %>%
  dplyr::select(geneSymbol:logFC.y, adj.P.Val.y) %>%
  dplyr::rename(female.vs.asexual_logFC = logFC.x,
                female.vs.asexual_FDR = adj.P.Val.x,
                mouse.vs.culture_female_logFC = logFC.y,
                mouse.vs.culture_female_FDR = adj.P.Val.y)

# save the resulting R data object for later use
write_tsv(DiffGenes.table, "DEGs_sheet1.txt")

# plotting heatmap
heatmap.2(DiffGenes,
          Rowv=as.dendrogram(clustRows),
          Colv=NA,
          RowSideColors=module.color,
          col=myheatcol, scale='row',
          labRow=NA, key = 1,
          density.info="none", trace="none",
          margins = c(10,25),
          dendrogram="none",
          cexRow=1, cexCol=0.75)
```

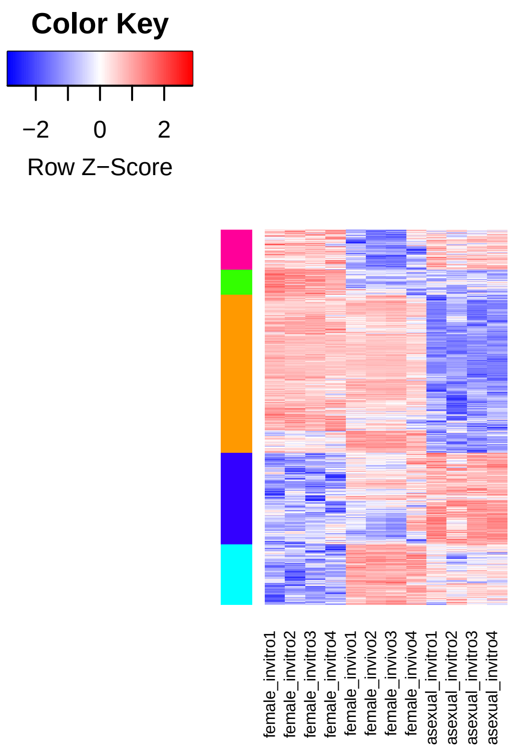

1477 out of a total of **3099** were identified as differentially expressed between females and asexual stages sorted from cultures, and/or between females from culture versus females sorted from mice. The table and venn below show up and down regulated genes for each pairwise comparison.

```
##        female.vs.asexual_invitro female_invivo.vs.invitro
## Down                         451                      317
## NotSig                      1975                     2388
## Up                           673                      394
```

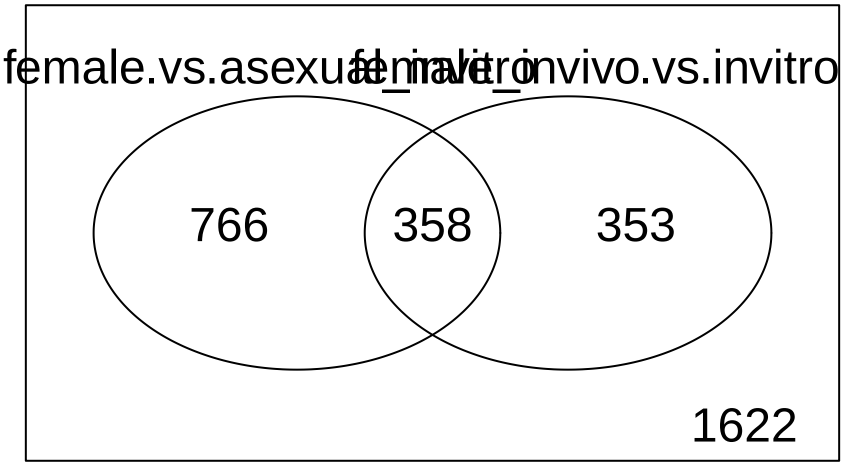

8 Functional annotation and enrichment analysis

Gene Set Enrichment Analysis was carried out outside of R/bioconductor using the Broad Institute's GSEA software. Four custom gene signatures for *C. parvum* were generated using gene ontology or community datasets available on CryptoDB. A 28 gene signature for 'carbohydrate metabolism' was generated using the Gene Ontology term GO:0005975. A 63 gene signature for 'DNA metabolic process' was generated using GO:0006259. A 48 gene signature for 'oxidation-reduction' was generated using GO:0055114. An 85 gene oocyst signature was generated by mining a published oocyst wall proteome dataset from *Truong and Ferrari, 2006* to retrieve only genes that had >= 20 unique peptide sequences per sample. All four signatures were used for GSEA analysis with 1000 permutations of gene sets to generate P values and multiple testing correction was applied to generate FDRs. The resulting enrichment data was used to make plots shown in **Figure 3B and 3C** in the manuscript, using DataGraph. The 'leading edge' genes that comprise the most enriched subset from each of the four signatures was then highlighted with colored points on the volcano plots to produce **Figure 3E and 3F** for the manuscript.

8.1 signatures for GSEA analysis

```
signatures <- read_tsv("../functionalEnrichmentAnalysis/cryptoPathways.gmx")
gt(signatures)
```

oxidation-reduction	DNA_metab	carb_metab	oocyst_proteomics_20
CryptoDB.org; GO:0055114	CryptoDB.org; GO:0006259	CryptoDB.org; GO:0005975	Truong and Ferrari, 2006. Gen
cgd1_3440	Cgd3_2720	cgd1_2040	cgd1_2040
cgd2_210	cgd1_1420	cgd1_3020	cgd1_3020

cgd2_2510	cgd1_310	cgd1_3060	cgd1_3170
cgd2_3570	cgd2_1100	cgd2_210	cgd1_330
cgd2_4320	cgd2_1250	cgd2_2130	cgd1_3710
cgd3_2050	cgd2_1600	cgd2_3200	cgd1_3780
cgd3_2180	cgd2_2060	cgd2_3260	cgd1_3810
cgd3_3120	cgd2_2500	cgd2_3270	cgd1_590
cgd3_3430	cgd2_3180	cgd3_1400	cgd1_640
cgd3_3910	cgd2_40	cgd3_1580	cgd1_750
cgd3_460	cgd2_4070	cgd4_2600	cgd2_20
cgd3_990	cgd2_510	cgd4_3310	cgd2_2700
cgd4_1330	cgd2_700	cgd5_1960	cgd2_3110
cgd4_2700	cgd3_1450	cgd5_2910	cgd2_3200
cgd4_2900	cgd3_3110	cgd5_3140	cgd2_3260
cgd4_4460	cgd3_3170	cgd6_2450	cgd2_4320
cgd4_690	cgd3_3820	cgd6_3280	cgd2_490
cgd4_740	cgd3_390	cgd6_3750	cgd2_790
cgd5_2440	cgd4_1490	cgd6_3790	cgd3_1290
cgd5_2670	cgd4_1930	cgd6_3800	cgd3_1400
cgd5_3230	cgd4_2053	cgd7_4270	cgd3_1770
cgd5_70	cgd4_3920	cgd7_470	cgd3_3370
cgd5_750	cgd4_430	cgd7_480	cgd3_3430
cgd6_1950	cgd4_440	cgd7_910	cgd3_3770
cgd6_20	cgd4_780	cgd8_1420	cgd4_2260
cgd6_2470	cgd4_970	cgd8_1920	cgd4_2300
cgd6_3280	cgd5_1180	cgd8_2160	cgd4_2600
cgd6_3720	cgd5_2560	cgd8_4940	cgd4_3090
cgd6_3750	cgd5_410	NA	cgd4_3160
cgd6_3790	cgd6_1580	NA	cgd4_3270
cgd6_3863	cgd6_1710	NA	cgd4_3530
cgd6_690	cgd6_1940	NA	cgd5_1490
cgd6_700	cgd6_1950	NA	cgd5_1580
cgd7_1000	cgd6_2390	NA	cgd5_1640
cgd7_1900	cgd6_240	NA	cgd5_1960
cgd7_270	cgd6_2610	NA	cgd5_2070
cgd7_470	cgd6_4420	NA	cgd5_3160
cgd7_480	cgd6_4783	NA	cgd5_4400
cgd7_4933	cgd6_5040	NA	cgd5_70
cgd8_1433	cgd7_1690	NA	cgd5_750
cgd8_1700	cgd7_1720	NA	cgd6_120
cgd8_1720	cgd7_2140	NA	cgd6_200
cgd8_2330	cgd7_2390	NA	cgd6_2090
cgd8_2670	cgd7_2920	NA	cgd6_2450
cgd8_3190	cgd7_3110	NA	cgd6_3050
cgd8_380	cgd7_3350	NA	cgd6_3080
cgd8_4230	cgd7_4730	NA	cgd6_3190
cgd8_920	cgd8_1240	NA	cgd6_3790
NA	cgd8_1350	NA	cgd6_3920
NA	cgd8_1410	NA	cgd6_3990
NA	cgd8_1620	NA	cgd6_4460
NA	cgd8_1630	NA	cgd6_4760
NA	cgd8_1940	NA	cgd6_5440
NA	cgd8_2020	NA	cgd6_880
NA	cgd8_2380	NA	cgd7_1270
NA	cgd8_2940	NA	cgd7_1340

NA	cgd8_370	NA	cgd7_1730
NA	cgd8_3950	NA	cgd7_1830
NA	cgd8_4650	NA	cgd7_1890
NA	cgd8_4950	NA	cgd7_1900
NA	cgd8_5410	NA	cgd7_2250
NA	cgd8_610	NA	cgd7_300
NA	cgd8_870	NA	cgd7_3120
NA	NA	NA	cgd7_360
NA	NA	NA	cgd7_3670
NA	NA	NA	cgd7_3790
NA	NA	NA	cgd7_4020
NA	NA	NA	cgd7_4280
NA	NA	NA	cgd7_4450
NA	NA	NA	cgd7_4500
NA	NA	NA	cgd7_4760
NA	NA	NA	cgd7_480
NA	NA	NA	cgd7_4810
NA	NA	NA	cgd7_5000
NA	NA	NA	cgd7_910
NA	NA	NA	cgd8_1270
NA	NA	NA	cgd8_1720
NA	NA	NA	cgd8_2790
NA	NA	NA	cgd8_2930
NA	NA	NA	cgd8_3430
NA	NA	NA	cgd8_350
NA	NA	NA	cgd8_3520
NA	NA	NA	cgd8_3900
NA	NA	NA	cgd8_430
NA	NA	NA	cgd8_440

8.2 GSEA using CAMERA

Functional enrichment analysis for the manuscript was carried out using the Broad Institute's GSEA software. However, we also include an analysis below using CAMERA.

```
#capturing each individual signature from above
redox.sig <- signatures$`oxidation-reduction`[-1]
meiosis.sig <- signatures$DNA_metab[-1]
carb.sig <- signatures$carb_metab[-1]
oocyst.sig <- signatures$oocyst_proteomics_20[-1]
# assembling into a gene set 'collection'
collection <- list(redox = redox.sig,
                   meiosis = meiosis.sig,
                   carb_metab = carb.sig,
                   oocyst_proteome = oocyst.sig)
# now test for enrichment in females vs asexual using CAMERA
GSEAres <- camera(v.myDGEList.filtered.norm$E, collection, design, contrast.matrix[,1])
GSEAres <- as_tibble(GSEAres, rownames = 'gene signature')
gt(GSEAres)
```

gene signature	NGenes	Direction	PValue	FDR
redox	43	Up	1.378358e-05	5.420079e-05

carb_metab	27	Up	2.710040e-05	5.420079e-05
meiosis	56	Up	2.317329e-04	3.089772e-04
oocyst_proteome	85	Up	2.828829e-01	2.828829e-01

8.3 reading in leading edge genes from GSEA analysis

```
# reading in the leading edge for each of the 4 signatures
leadingEdge <- read_tsv("../functionalEnrichmentAnalysis/leadingEdge.txt")
#carbohydrate metabolism
carb_metab <- leadingEdge %>%
  dplyr::rename(geneSymbol = carbohydrate_metabolism) %>%
  dplyr::select(-"oocyst_proteomics", -"DNA_metab", -"oxidation-reduction") %>%
  dplyr::left_join(normData, by="geneSymbol") %>%
  dplyr::filter(!is.na(geneSymbol))

carb_metab <- as.matrix(column_to_rownames(carb_metab, 'geneSymbol'))

#oocyst wall proteome
oocyst_proteomics <- leadingEdge %>%
  dplyr::rename(geneSymbol = oocyst_proteomics) %>%
  dplyr::select(-"carbohydrate_metabolism", -"DNA_metab", -"oxidation-reduction") %>%
  dplyr::left_join(normData, by="geneSymbol") %>%
  dplyr::filter(!is.na(geneSymbol))

oocyst_proteomics <- as.matrix(column_to_rownames(oocyst_proteomics, 'geneSymbol'))

#meiosis and DNA replication/metabolism
DNA_metab <- leadingEdge %>%
  dplyr::rename(geneSymbol = DNA_metab) %>%
  dplyr::select(-"carbohydrate_metabolism", -"oocyst_proteomics", -"oxidation-reduction") %>%
  dplyr::left_join(normData, by="geneSymbol") %>%
  dplyr::filter(!is.na(geneSymbol))

DNA_metab <- as.matrix(column_to_rownames(DNA_metab, 'geneSymbol'))

#oxidation-reduction
oxidoreductase <- leadingEdge %>%
  dplyr::rename(geneSymbol = "oxidation-reduction") %>%
  dplyr::select(-"carbohydrate_metabolism", -"oocyst_proteomics", -"DNA_metab") %>%
  dplyr::left_join(normData, by="geneSymbol") %>%
  dplyr::filter(!is.na(geneSymbol))

oxidoreductase <- as.matrix(column_to_rownames(oxidoreductase, 'geneSymbol'))
```

8.4 Volcano plot: females vs asexual stage from culture - Figure 3E

```
# subsetting volcano plot datq based on leading edge genes
myTopHits1.carb_metab <- subset(myTopHits1, geneSymbol %in% rownames(carb_metab))
myTopHits1.oocyst_proteomics <- subset(myTopHits1, geneSymbol %in% rownames(oocyst_proteomics))
myTopHits1.DNA_metab <- subset(myTopHits1, geneSymbol %in% rownames(DNA_metab))
myTopHits1.oxidoreductase <- subset(myTopHits1, geneSymbol %in% rownames(oxidoreductase))
```

```
# replotting volcano plots with leading edge genes highlighted
ggplot(myTopHits1, aes(y=-log10(adj.P.Val), x=logFC, text = paste("Symbol:", geneSymbol))) +
  geom_point(size=4, alpha = 1/10) +
  coord_fixed() +
  geom_point(mapping =NULL, myTopHits1.carb_metab, size = 4, colour= "#ED3624", inherit.aes = TRUE) +
  geom_point(mapping =NULL, myTopHits1.oocyst_proteomics, size = 4, colour= "#4492C4", inherit.aes = TRU
  geom_point(mapping =NULL, myTopHits1.DNA_metab, size = 4, colour= "#5BB95B", inherit.aes = TRUE) +
  geom_point(mapping =NULL, myTopHits1.oxidoreductase, size = 4, colour= "#AA67B2", inherit.aes = TRUE)
  ylim(-0.5,15) +
  xlim(-15,15) +
  geom_hline(yintercept = -log10(0.01), linetype="longdash", colour="grey", size=2) +
  geom_vline(xintercept = 1, linetype="longdash", colour="#BE684D", size=2) +
  geom_vline(xintercept = -1, linetype="longdash", colour="#2C467A", size=2) +
  #labs(title="females vs. asexual stages sorted from culture",
       #subtitle = "Volcano plot comparing expression of genes between females and asexual stages \nsor
  theme_bw() +
  theme(axis.text=element_text(size=16),
        axis.title=element_text(size=18),
        plot.title = element_text(face="bold"),
        panel.border = element_rect(colour = "black", fill=NA, size=1))
```

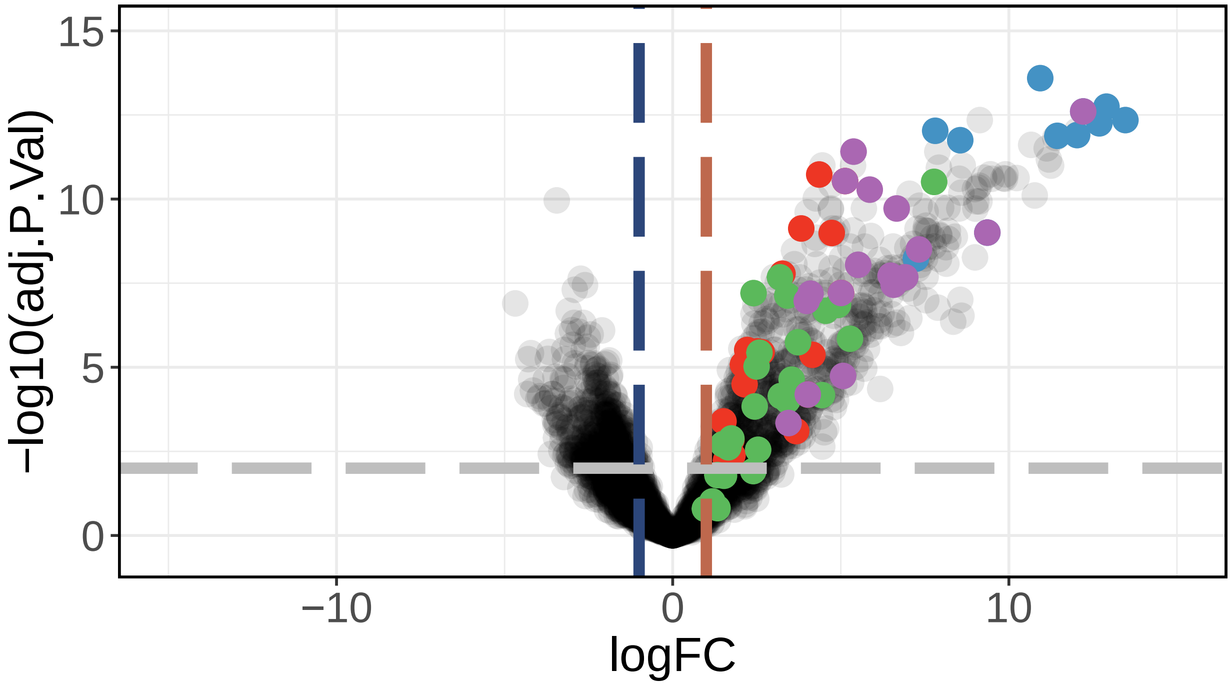

8.5 Volcano plot: females sorted from culture versus mouse infection - Figure 3F

```
# genes known to be part of the gliding machinery for C. parvum
gliding <-c("cgd6_4460", "cgd6_2220", "cgd6_1500", "cgd7_550",
            "cgd6_2210", "cgd2_640", "cgd7_4420", "cgd7_3790")

myTopHits2.carb_metab <- subset(myTopHits2, geneSymbol %in% rownames(carb_metab))
myTopHits2.oocyst_proteomics <- subset(myTopHits2, geneSymbol %in% rownames(oocyst_proteomics))
myTopHits2.DNA_metab <- subset(myTopHits2, geneSymbol %in% rownames(DNA_metab))
myTopHits2.oxidoreductase <- subset(myTopHits2, geneSymbol %in% rownames(oxidoreductase))
myTopHits2.gliding <- subset(myTopHits2, geneSymbol %in% gliding)

ggplot(myTopHits2, aes(y=-log10(adj.P.Val), x=logFC, text = paste("Symbol:", geneSymbol))) +
  geom_point(size=4, alpha = 1/10) +
  coord_fixed() +
  geom_point(mapping =NULL, myTopHits2.carb_metab, size = 4, colour= "#ED3624", inherit.aes = TRUE) +
  geom_point(mapping =NULL, myTopHits2.oocyst_proteomics, size = 4, colour= "#4492C4", inherit.aes = TRU
  geom_point(mapping =NULL, myTopHits2.DNA_metab, size = 4, colour= "#5BB95B", inherit.aes = TRUE) +
  geom_point(mapping =NULL, myTopHits2.oxidoreductase, size = 4, colour= "#AA67B2", inherit.aes = TRUE)
  geom_point(mapping =NULL, myTopHits2.gliding, size = 4, colour= "#F0E342", inherit.aes = TRUE) +
  ylim(-0.5,15) +
  xlim(-15,15) +
  geom_hline(yintercept = -log10(0.01), linetype="longdash", colour="grey", size=2) +
  geom_vline(xintercept = 1, linetype="longdash", colour="#BE684D", size=2) +
  geom_vline(xintercept = -1, linetype="longdash", colour="#2C467A", size=2) +
  #labs(title="Females from infected mice vs culture",
       #subtitle = "Volcano plot comparing expression of genes between females \nsorted from in infecte
  theme_bw() +
  theme(axis.text=element_text(size=16),
        axis.title=element_text(size=18),
        plot.title = element_text(face="bold"),
        panel.border = element_rect(colour = "black", fill=NA, size=1))
```

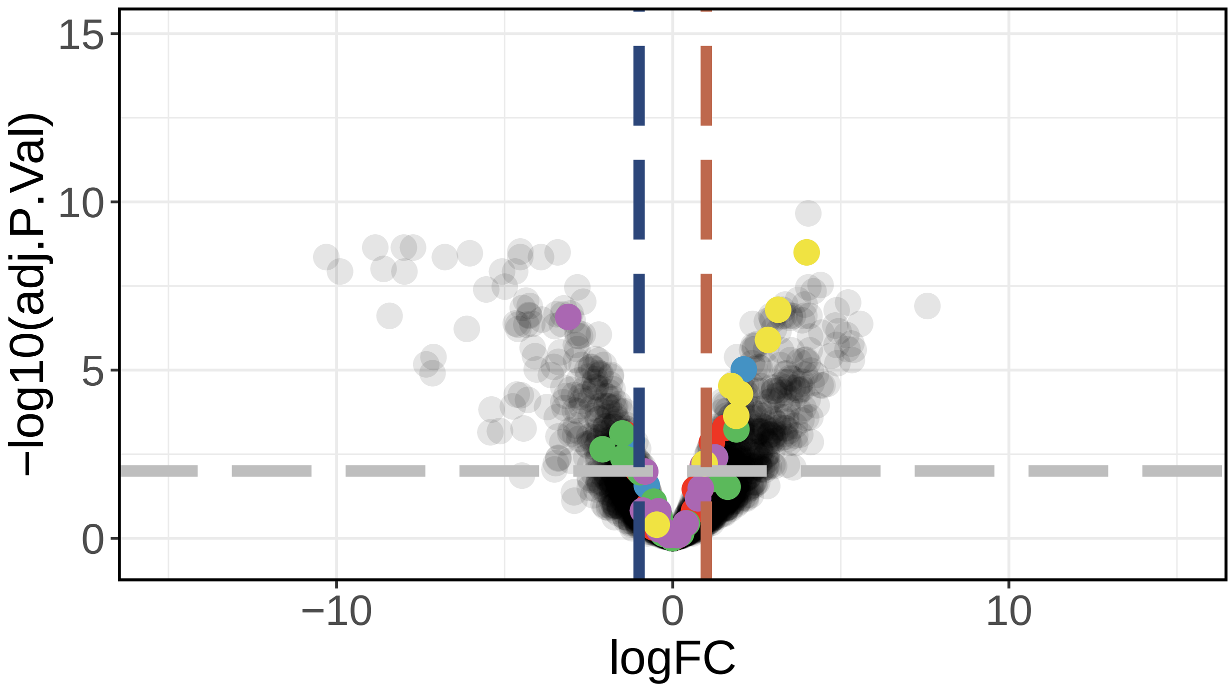

8.6 heatmap: gliding machinery - Figure 3G

```
gliding.data <- as_tibble(v.myDGEList.filtered.norm$E, rownames = "geneSymbol") %>%
  dplyr::filter(geneSymbol=="cgd6_4460" | geneSymbol=="cgd6_2220" |
                geneSymbol=="cgd6_1500" | geneSymbol=="cgd7_550" |
                geneSymbol=="cgd6_2210" | geneSymbol=="cgd2_640" |
                geneSymbol=="cgd7_4420" | geneSymbol=="cgd7_3790") %>%
  dplyr::select(geneSymbol,asexual_invitro1, asexual_invitro2, asexual_invitro3, asexual_invitro4,
                female_invitro1, female_invitro2, female_invitro3, female_invitro4,
                female_invivo1, female_invivo2, female_invivo3, female_invivo4)

gliding.data <-column_to_rownames(gliding.data, var="geneSymbol")
gliding.matrix <- data.matrix(gliding.data)

hrGliding <- hclust(as.dist(1-cor(t(gliding.matrix), method="pearson")), method="complete")

heatmap.2(gliding.matrix, Rowv=as.dendrogram(hrGliding), Colv=NA,
          col=myheatcol, scale="row", density.info="none",
          trace="none",
          cexRow=1, cexCol=0.75, margins=c(10,20),
          dendrogram = "none")
```

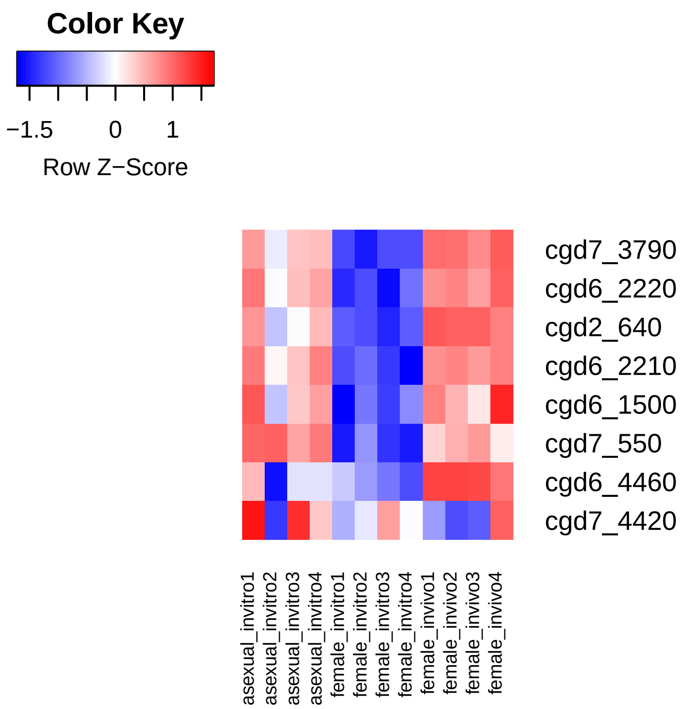

---

9 Identification of the sexual differentiation program in *C. parvum*.

9.1 filtering and normalization

```
load("Txi_gene")

# taking only the last 9 samples in the dataset,
# which correspond to batch 1
Txi_gene2 <- Txi_gene$counts[,13:21]
# use EdgeR create DGEList object from counts
myDGEList2 <- DGEList(Txi_gene2)
# use the 'cpm' function from EdgeR to get counts per million
log2.cpm2 <- cpm(myDGEList2, log=TRUE)
log2.cpm.df2 <- as_tibble(log2.cpm2)
colnames(log2.cpm.df2) <- SampleLabels[13:21]
log2.cpm.df2 <- melt(log2.cpm.df2)
colnames(log2.cpm.df2) <- c("sample", "expression")

# plot of signal distribution for raw data
p1 <- ggplot(log2.cpm.df2, aes(x=sample, y=expression, fill=sample)) +
  geom_violin(trim = FALSE, show.legend = FALSE) +
  stat_summary(fun.y = "median",
```

```
               geom = "point",
               shape = 124, size = 6,
               color = "black",
               show.legend = FALSE) +
  labs(y="log2 expression", x = "sample",
       title = "raw data") +
  coord_flip() +
  theme_bw()

# filtering to keep only genes that had > 10 cpm in at least 3 samples
cpm2 <- cpm(myDGEList2)
keepers2 <- rowSums(cpm2>10)>=3
myDGEList.filtered2 <- myDGEList2[keepers2,]
# normalize using TMM method from calnormfactors function in EdgeR package
myDGEList.filtered.norm2 <- calcNormFactors(myDGEList.filtered2, method = "TMM")
log2.cpm.filtered.norm2 <- cpm(myDGEList.filtered.norm2, log=TRUE)
log2.cpm.filtered.norm.df2 <- as_tibble(log2.cpm.filtered.norm2)
colnames(log2.cpm.filtered.norm.df2) <- SampleLabels[13:21]
log2.cpm.filtered.norm.df2 <- melt(log2.cpm.filtered.norm.df2)
colnames(log2.cpm.filtered.norm.df2) <- c("sample", "expression")

normData2 <- as_tibble(log2.cpm.filtered.norm2, rownames = "geneSymbol")
colnames(normData2) <- c("geneSymbol", SampleLabels[13:21])
write_tsv(normData2, "normData2.txt")

# plot of signal distribution again to see effect of filtering an normalization
p2 <- ggplot(log2.cpm.filtered.norm.df2, aes(x=sample, y=expression, fill=sample)) +
  geom_violin(trim = FALSE, show.legend = FALSE) +
  stat_summary(fun.y = "median",
               geom = "point",
               shape = 124, size = 6,
               color = "black",
               show.legend = FALSE) +
  labs(y="log2 expression", x = "sample",
       title = "filtered, normalized data") +
  coord_flip() +
  theme_bw()

plot_grid(p1, p2, labels = c("A", "B"))
```

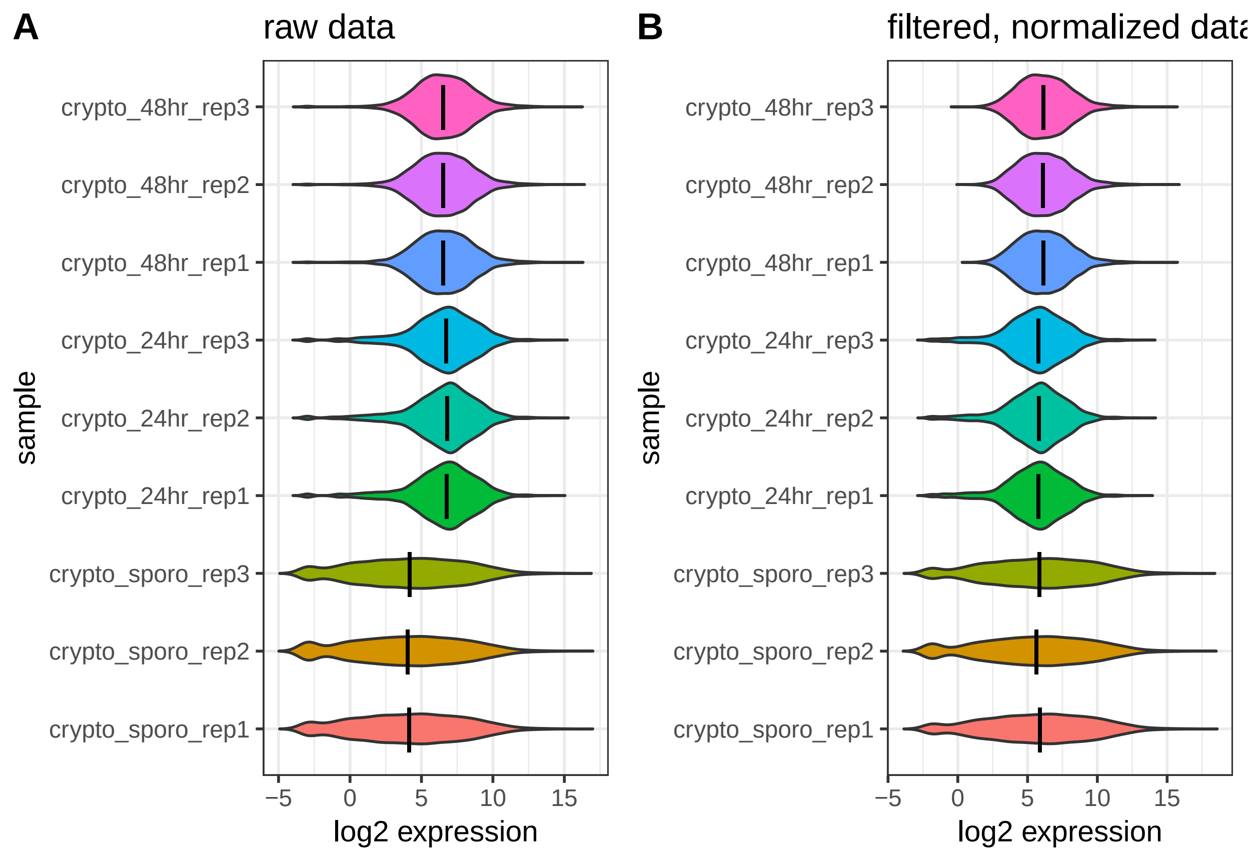

9.2 PCA of data after filtering and normalization

```
# running PCA
pca.res2 <- prcomp(t(log2.cpm.filtered.norm2), scale.=F, retx=T)
pc.var2<-pca.res2$sdev^2
pc.per2<-round(pc.var2/sum(pc.var2)*100, 1)

# converting PCA result into a tibble for plotting
pca.res.df2 <- as_tibble(pca.res2$x)
# plotting PCA
ggplot(pca.res.df2, aes(x=PC1, y=PC2, color=sex[13:21], shape=origin[13:21])) +
  geom_point(size=4) +
  theme(legend.position="right") +
  xlab(paste0("PC1 (",pc.per[1],"%",")")) +
  ylab(paste0("PC2 (",pc.per[2],"%",")")) +
  labs(title="PCA of sporozoites vs in vitro timecourse",
       subtitle = "Principal component analysis (PCA) showing separation \nbetween sporozoites and both
       color = "sex", shape="origin") +
  theme_bw() +
  theme(plot.title = element_text(face="bold"))
```

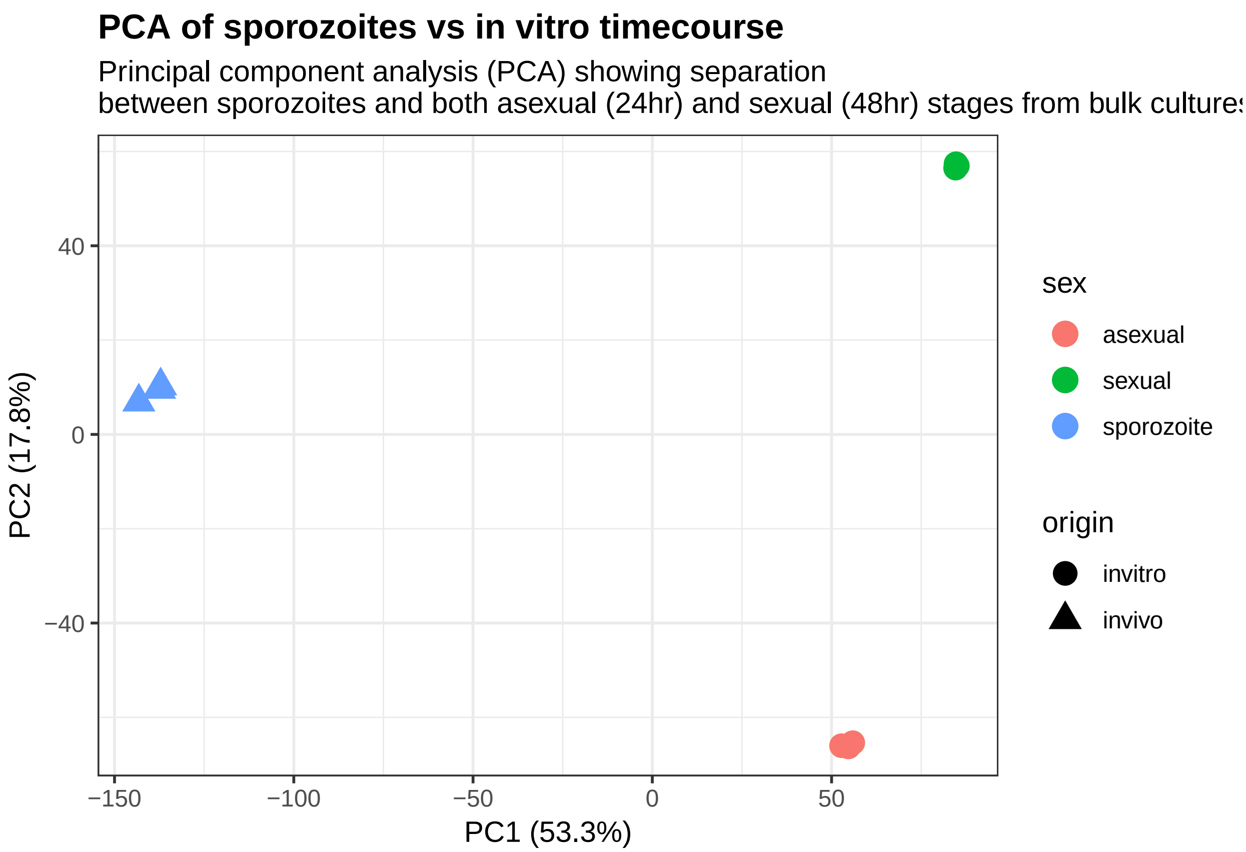

9.3 Volcano plot: sexual development in bulk culture - Supp. Fig 8A

Many genes in the upper right of the plot, which represent genes that are significantly **higher** at 48 hrs in bulk culture, compared to 24 hr. These represent genes involved in sexualization of *C. parvum*.

```
# setting up model matrix without an intercept
design2 <- model.matrix(~0 + group[13:21, drop = TRUE])
colnames(design2) <- levels(group[13:21, drop = TRUE])

# using VOOM function from Limma package to apply precision weights to each gene
v.myDGEList.filtered.norm2 <- voom(myDGEList.filtered.norm2, design2, plot = FALSE)
fit2 <- lmFit(v.myDGEList.filtered.norm2, design2)

# setting up contrast matrix for two main pairwise comparisons
contrast.matrix2 <- makeContrasts(sexualDevelop = sexual_invitro - asexual_invitro,
                                  asexualGrowth = asexual_invitro - sporozoite_invivo,
                                  levels=design2)

fits2 <- contrasts.fit(fit2, contrast.matrix2)
# extracting stats
ebFit2 <- eBayes(fits2)
# listing stats for all genes in the dataset to be used for making volcano plot
myTopHits3 <- topTable(ebFit2, adjust ="BH", coef=1, number=10000, sort.by="logFC")
myTopHits3 <- as_tibble(myTopHits3, rownames = "geneSymbol")
```

```r
# subsetting volcano plot datq based on leading edge genes
myTopHits3.carb_metab <- subset(myTopHits3, geneSymbol %in% rownames(carb_metab))
myTopHits3.oocyst_proteomics <- subset(myTopHits3, geneSymbol %in% rownames(oocyst_proteomics))
myTopHits3.DNA_metab <- subset(myTopHits3, geneSymbol %in% rownames(DNA_metab))
myTopHits3.oxidoreductase <- subset(myTopHits3, geneSymbol %in% rownames(oxidoreductase))

# volcano plot
ggplot(myTopHits3, aes(y=-log10(adj.P.Val), x=logFC, text = paste("Symbol:", geneSymbol))) +
  geom_point(size=4, alpha = 1/10) +
  geom_point(mapping =NULL, myTopHits3.carb_metab, size = 4, colour= "#ED3624", inherit.aes = TRUE) +
  geom_point(mapping =NULL, myTopHits3.oocyst_proteomics, size = 4, colour= "#4492C4", inherit.aes = TRU
  geom_point(mapping =NULL, myTopHits3.DNA_metab, size = 4, colour= "#5BB95B", inherit.aes = TRUE) +
  geom_point(mapping =NULL, myTopHits3.oxidoreductase, size = 4, colour= "#AA67B2", inherit.aes = TRUE)
  ylim(-0.5,15) +
  xlim(-15,15) +
  geom_hline(yintercept = -log10(0.01), linetype="longdash", colour="grey", size=1) +
  geom_vline(xintercept = 1, linetype="longdash", colour="#BE684D", size=1) +
  geom_vline(xintercept = -1, linetype="longdash", colour="#2C467A", size=1) +
  labs(title="sexual vs. asexual stages from bulk culture",
       subtitle = "Volcano plot comparing expression of genes between sexual (24hr) and asexual stages
       \nThis plot corresponds to Supp. Figure 9A in the manuscript.") +
  theme_bw() +
  theme(axis.text=element_text(size=16),
        axis.title=element_text(size=18),
        plot.title = element_text(face="bold"),
        panel.border = element_rect(colour = "black", fill=NA, size=1))
```

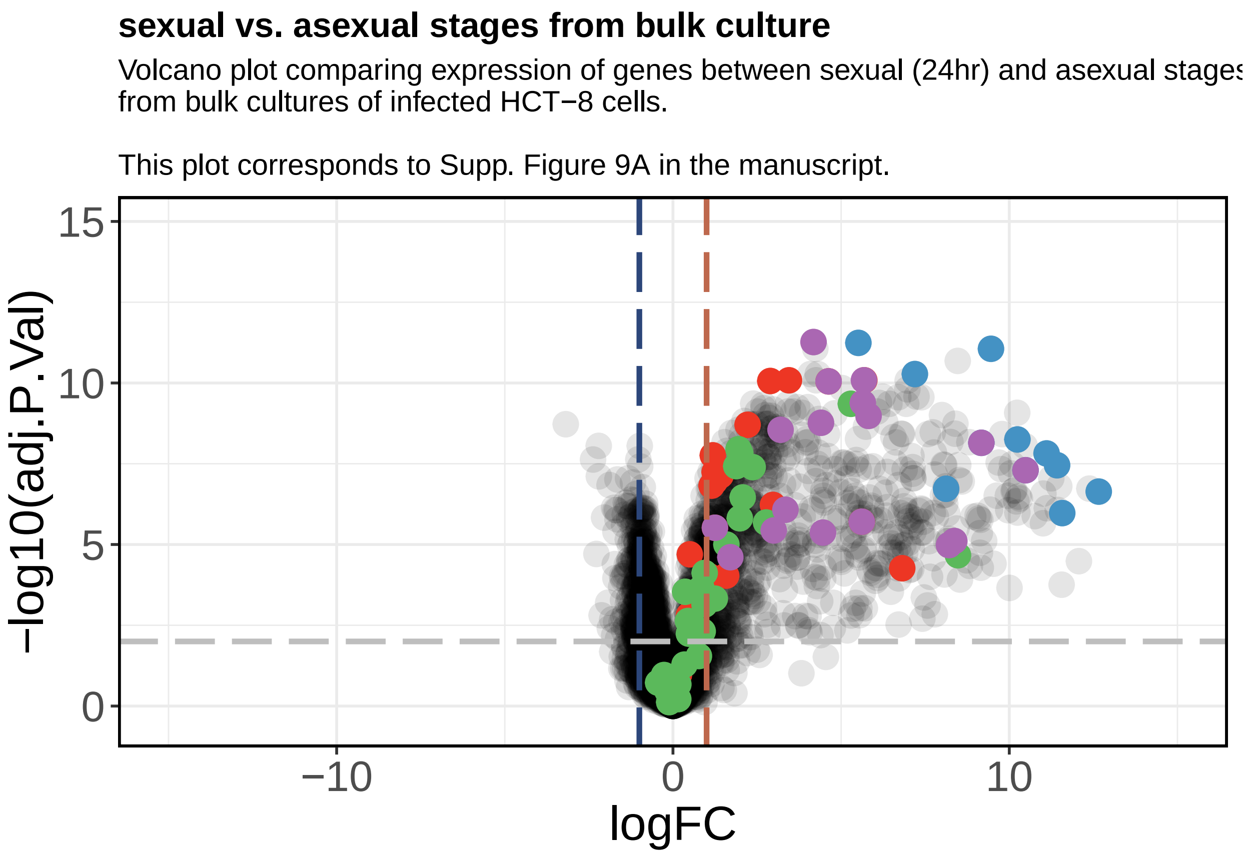

9.4 Volcano plot: asexual growth

```
myTopHits4 <- topTable(ebFit2, adjust ="BH", coef=2, number=10000, sort.by="logFC")
myTopHits4 <- as_tibble(myTopHits4, rownames = "geneSymbol")
# volcano plot
ggplot(myTopHits4, aes(y=-log10(adj.P.Val), x=logFC, text = paste("Symbol:", geneSymbol))) +
  geom_point(size=2) +
  ylim(-0.5,15) +
  xlim(-15,15) +
  geom_hline(yintercept = -log10(0.01), linetype="longdash", colour="grey", size=1) +
  geom_vline(xintercept = 1, linetype="longdash", colour="#BE684D", size=1) +
  geom_vline(xintercept = -1, linetype="longdash", colour="#2C467A", size=1) +
  labs(title="sporozoite vs asexual",
       subtitle = "Volcano plot comparing expression of genes between sporozoites \nand asexual stages f
  theme_bw() +
  theme(axis.text=element_text(size=16),
        axis.title=element_text(size=18),
        plot.title = element_text(face="bold"),
        panel.border = element_rect(colour = "black", fill=NA, size=1))
```

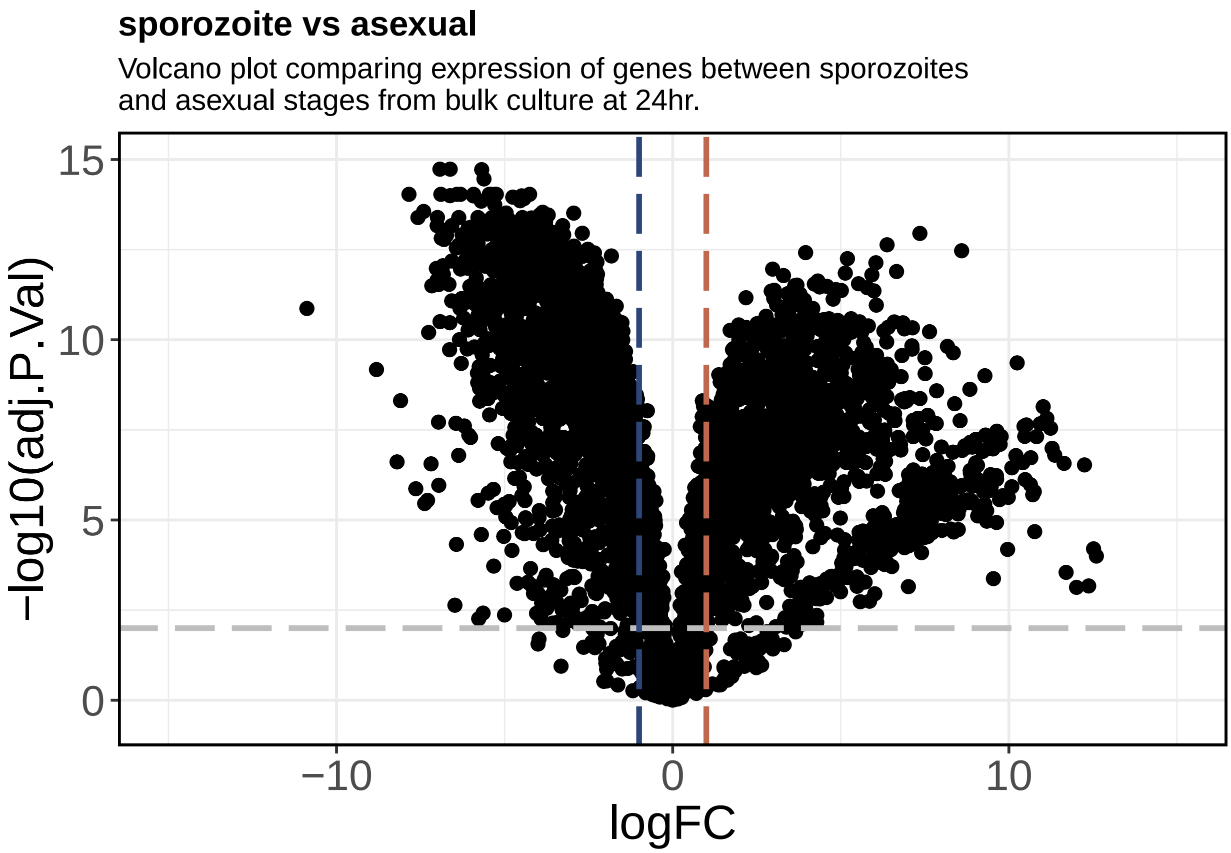

9.5 Heatmap: identification of co-expression modules - Supp. Fig 8B

```r
colnames(v.myDGEList.filtered.norm2$E) <- SampleLabels[13:21]
# using decideTests to identify DEGs based on FDR and logFC
results2 <- decideTests(ebFit2, method="global", adjust.method="BH", p.value=0.01, lfc=1)
# pulling these genes out along with their expression data
DiffGenes2 <- v.myDGEList.filtered.norm2$E[results2[,1] !=0 | results2[,2] !=0,]
clustRows2 <- hclust(as.dist(1-cor(t(DiffGenes2), method="pearson")), method="average")
clustColumns2 <- hclust(as.dist(1-cor(DiffGenes2, method="spearman")), method="complete")
clust.assign2 <- cutree(clustRows2, k=6)

module.color2 <- rainbow(length(unique(clust.assign2)), start=0.1, end=0.9)
module.color2 <- module.color2[as.vector(clust.assign2)]

#construct a table of the DEGs with stats for saving
DiffGenes2.table <- as_tibble(DiffGenes2, rownames = "geneSymbol")
DiffGenes2.table <- DiffGenes2.table %>%
  dplyr::left_join(myTopHits3, by="geneSymbol") %>%
  dplyr::select(geneSymbol:logFC, adj.P.Val) %>%
  dplyr::left_join(myTopHits4, by="geneSymbol") %>%
  dplyr::select(geneSymbol:logFC.y, adj.P.Val.y) %>%
  dplyr::rename(sexual.24hr.vs.asexual.48hr_logFC = logFC.x,
                sexual.24hr.vs.asexual.48hr_FDR = adj.P.Val.x,
                sporozoite.vs.asexual.48hr_logFC = logFC.y,
                sporozoite.vs.asexual.48hr_FDR = adj.P.Val.y)

# save the resulting R data object for later use
write_tsv(DiffGenes2.table, "DEGs_sheet2.txt")

# plotting heatmap
heatmap.2(DiffGenes2,
          Rowv=as.dendrogram(clustRows2),
          Colv=NA,
          RowSideColors=module.color2,
          col=myheatcol, scale='row',
          labRow=NA, key = 1,
          density.info="none", trace="none",
          margins = c(10,25),
          dendrogram="none",
          cexRow=1, cexCol=0.75)
```

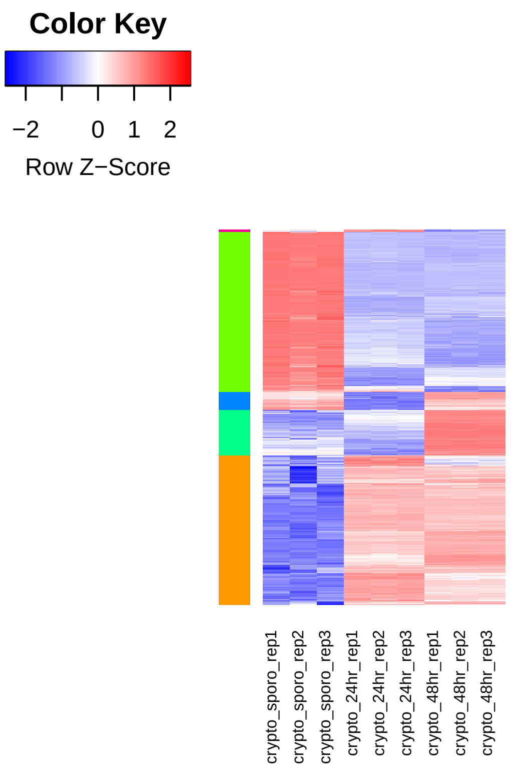

3137 out of a total of **3702** were identified as differentially expressed between sexual (48hr) versus asexual stages (24hr) from bulk cultures of infected HCT-8 cells, and/or between asexual stages from bulk culture (24hr) versus sporozoites. The table and venn below show up and down regulated genes for each pairwise comparison.

```
##        sexualDevelop asexualGrowth
## Down             171          1531
## NotSig          2765           827
## Up               766          1344
```

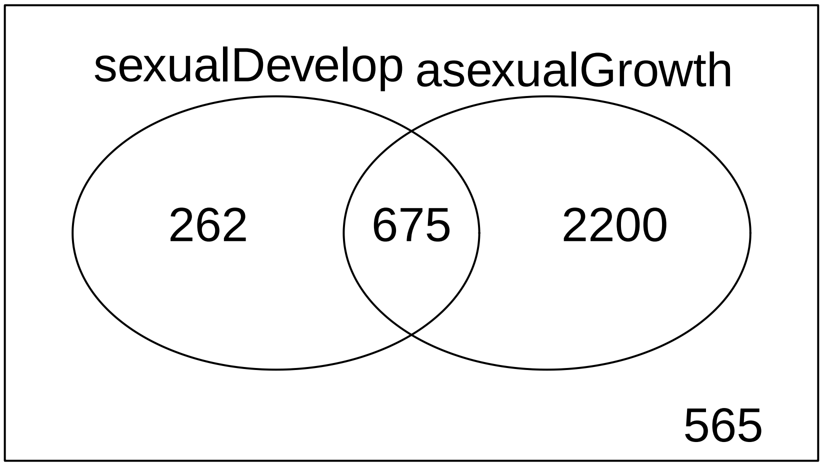

---

10 Function grouping of genes

10.1 heatmap: meiosis and DNA repair - Figure 4A

```
myMeiosis <- as_tibble(v.myDGEList.filtered.norm$E, rownames = "geneSymbol") %>%
  dplyr::filter(geneSymbol=="cgd7_1690"| geneSymbol=="cgd6_4420"|
                geneSymbol=="cgd8_1350"| geneSymbol=="cgd2_510" |
                geneSymbol=="cgd1_1420"| geneSymbol=="cgd1_60" |
                geneSymbol=="cgd3_4050"| geneSymbol=="cgd5_2790"|
                geneSymbol=="cgd4_2860"| geneSymbol=="cgd1_1180"|
                geneSymbol=="cgd8_610"| geneSymbol=="cgd7_2370"|
                geneSymbol=="cgd8_1620"| geneSymbol=="cgd3_3860"|
                geneSymbol=="cgd7_4620"| geneSymbol=="cgd3_4290"|
                geneSymbol=="cgd7_2700"| geneSymbol=="cgd7_2310"|
                geneSymbol=="cgd7_1850"| geneSymbol=="cgd8_490"|
                geneSymbol=="cgd6_4140"| geneSymbol=="cgd1_330"|
                geneSymbol=="cgd1_1330"| geneSymbol=="cgd3_2210"|
                geneSymbol=="cgd8_1410"| geneSymbol=="cgd6_4760"|
                geneSymbol=="cgd1_3670"| geneSymbol=="cgd5_2560"|
                geneSymbol=="cgd2_2750"| geneSymbol=="cgd2_2500"|
                geneSymbol=="cgd3_3820" | geneSymbol=="cgd5_1750"|
                geneSymbol=="cgd8_4950" | geneSymbol=="cgd5_410" |
                geneSymbol=="cgd3_3110" | geneSymbol=="cgd7_3110" |
```

```
                 geneSymbol=="cgd4_440" | geneSymbol=="cgd7_2140" |
                 geneSymbol=="cgd6_6040") %>%
  dplyr::select(geneSymbol,asexual_invitro1, asexual_invitro2, asexual_invitro3, asexual_invitro4,
                 female_invitro1, female_invitro2, female_invitro3, female_invitro4,
                 female_invivo1, female_invivo2, female_invivo3, female_invivo4)

myMeiosis <-column_to_rownames(myMeiosis, var="geneSymbol")
myMeiosis.matrix <- data.matrix(myMeiosis)

hrMei <- hclust(as.dist(1-cor(t(myMeiosis.matrix), method="pearson")), method="complete")

heatmap.2(myMeiosis.matrix, Rowv=as.dendrogram(hrMei), Colv=NA,
          col=myheatcol, scale="row", density.info="none",
          trace="none", key = 1, labRow = NA,
          cexRow=0.5, cexCol=0.75, margins=c(10,20),
          dendrogram = "none")
```

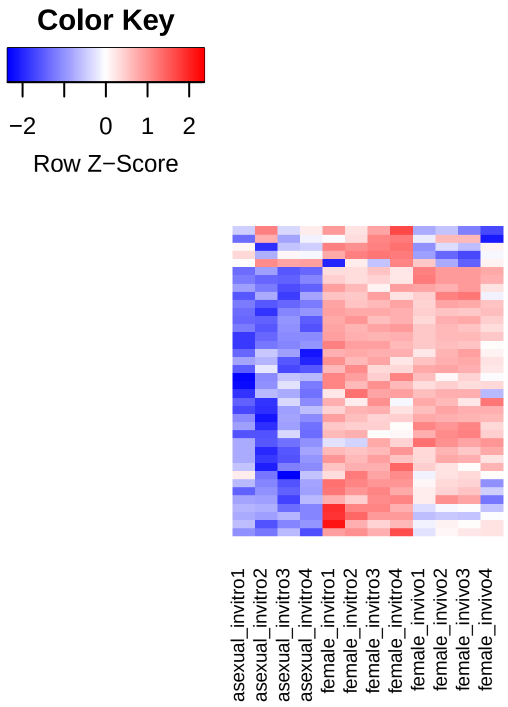

10.2 heatmaps: oocyst environmental resilience - Figure 4B

10.2.1 oocyst wall proteins

```
Owpx <- as_tibble(v.myDGEList.filtered.norm$E, rownames = "geneSymbol") %>%
  dplyr::filter(geneSymbol== "cgd6_2090" |
                  geneSymbol== "cgd6_200" |
```

```
                  geneSymbol== "cgd4_3090" |
                  geneSymbol== "cgd7_5150" |
                  geneSymbol== "cgd4_670" |
                  geneSymbol== "cgd7_1800" |
                  geneSymbol== "cgd8_3350" |
                  geneSymbol== "cgd4_500" |
                  geneSymbol== "cgd6_210" |
                  geneSymbol== "cgd7_4810" |
                  geneSymbol=="cgd7_300"|
                  geneSymbol=="cgd2_790"|
                  geneSymbol=="cgd7_3120"|
                  geneSymbol=="cgd7_1730"|
                  geneSymbol=="cgd2_490") %>%
      dplyr::select(geneSymbol,asexual_invitro1, asexual_invitro2, asexual_invitro3, asexual_invitro4,
                    female_invitro1, female_invitro2, female_invitro3, female_invitro4,
                    female_invivo1, female_invivo2, female_invivo3, female_invivo4)

Owpx <-column_to_rownames(Owpx, var="geneSymbol")
Owpx.matrix <- data.matrix(Owpx)

Owxphr <- hclust(as.dist(1-cor(t(Owpx.matrix), method="pearson")), method="complete")

heatmap.2(Owpx.matrix, Rowv=as.dendrogram(Owxphr),  Colv=NA,
          col=myheatcol, scale="row", density.info="none",
          trace="none", key = 1,
          cexRow=0.5, cexCol=0.75, margins=c(10,20),
          dendrogram = "none")
```

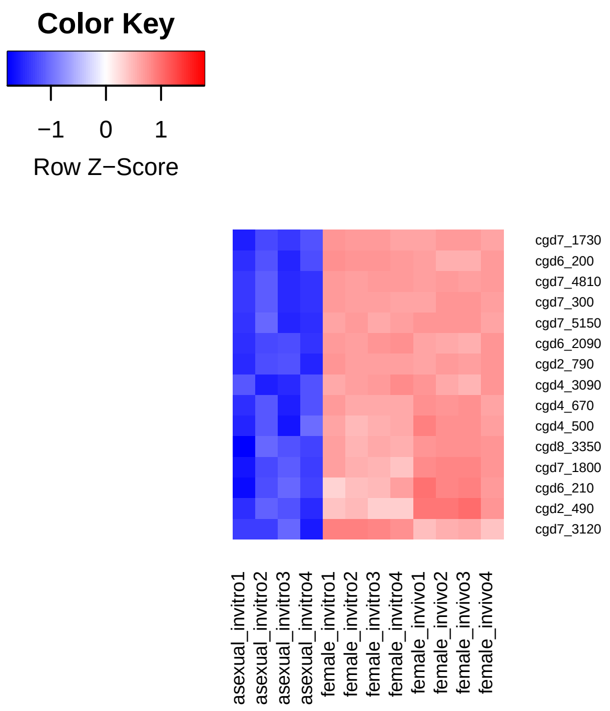

10.2.2 oxidoreductases

```
Oxi <- as_tibble(v.myDGEList.filtered.norm$E, rownames = "geneSymbol") %>%
  dplyr::filter(geneSymbol=="cgd8_1700" |
                geneSymbol=="cgd7_270" |
                geneSymbol=="cgd8_1720" |
                geneSymbol=="cgd4_4460" |
                geneSymbol=="cgd7_1000" |
                geneSymbol=="cgd8_4230" |
                geneSymbol=="cgd3_460" |
                geneSymbol=="cgd2_210" |
                geneSymbol=="cgd5_3230" |
                geneSymbol=="cgd6_3720" |
                geneSymbol=="cgd4_690" |
                geneSymbol=="cgd6_1950" |
                geneSymbol=="cgd1_280" |
                geneSymbol=="cgd6_2470" |
                geneSymbol=="cgd3_3430" |
                geneSymbol=="cgd2_2510"|
                geneSymbol=="cgd8_2670" |
                geneSymbol=="cgd8_380") %>%
  dplyr::select(geneSymbol,asexual_invitro1, asexual_invitro2, asexual_invitro3, asexual_invitro4,
               female_invitro1, female_invitro2, female_invitro3, female_invitro4,
               female_invivo1, female_invivo2, female_invivo3, female_invivo4)
```

```
Oxi <-column_to_rownames(Oxi, var="geneSymbol")
Oxi.matrix <- data.matrix(Oxi)

hrOxi <- hclust(as.dist(1-cor(t(Oxi.matrix), method="pearson")), method="complete")

heatmap.2(Oxi.matrix, Rowv=as.dendrogram(hrOxi), Colv=NA,
          col=myheatcol, scale="row", density.info="none",
          trace="none", key = 1,
          cexRow=0.5, cexCol=0.75, margins=c(10,20),
          dendrogram = "none")
```

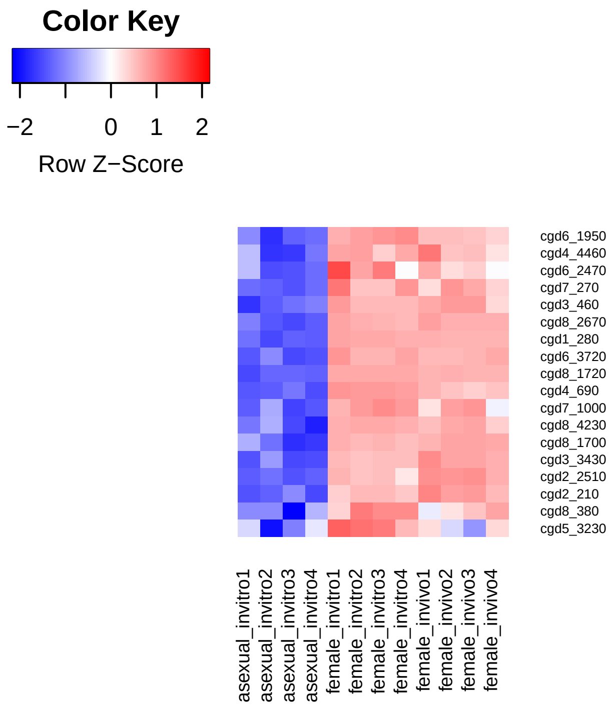

10.2.3 proteases

```
#Protease heatmap ----
Protease <- as_tibble(v.myDGEList.filtered.norm$E, rownames = "geneSymbol") %>%
  dplyr::filter(geneSymbol=="cgd5_2600" |
                geneSymbol=="cgd3_680" |
                geneSymbol=="cgd3_3610" |
                geneSymbol=="cgd1_3550" |
                geneSymbol=="cgd2_1590" |
                geneSymbol=="cgd6_3820" |
                geneSymbol=="cgd5_3940" |
                geneSymbol=="cgd1_370" |
```

```
                  geneSymbol=="cgd6_4880" |
                  geneSymbol=="cgd1_1680" |
                  geneSymbol=="cgd2_2760" |
                  geneSymbol=="cgd3_520" |
                  geneSymbol=="cgd2_3320" |
                  geneSymbol=="cgd4_2110" |
                  geneSymbol=="cgd5_2500" |
                  geneSymbol=="cgd6_900" |
                  geneSymbol=="cgd5_2660" |
                  geneSymbol=="cgd2_920" |
                  geneSymbol=="cgd5_4370" |
                  geneSymbol=="cgd3_4200" |
                  geneSymbol=="cgd6_4080" |
                  geneSymbol=="cgd6_3730" |
                  geneSymbol=="cgd1_740" |
                  geneSymbol=="cgd7_4730" |
                  geneSymbol=="cgd1_1100" |
                  geneSymbol=="cgd6_4840" |
                  geneSymbol=="cgd2_3660" |
                  geneSymbol=="cgd4_2190"  ) %>%
  dplyr::select(geneSymbol,asexual_invitro1, asexual_invitro2, asexual_invitro3, asexual_invitro4,
                female_invitro1, female_invitro2, female_invitro3, female_invitro4,
                female_invivo1, female_invivo2, female_invivo3, female_invivo4)

Protease <-column_to_rownames(Protease, var="geneSymbol")
Protease.matrix <- data.matrix(Protease)

hrPro <- hclust(as.dist(1-cor(t(Protease.matrix), method="pearson")), method="complete")

heatmap.2(Protease.matrix, Rowv=as.dendrogram(hrPro), Colv=NA,
          col=myheatcol, scale="row", density.info="none",
          trace="none", key = 1, labRow = NA,
          cexRow=0.5, cexCol=0.75, margins=c(10,20),
          dendrogram = "none")
```

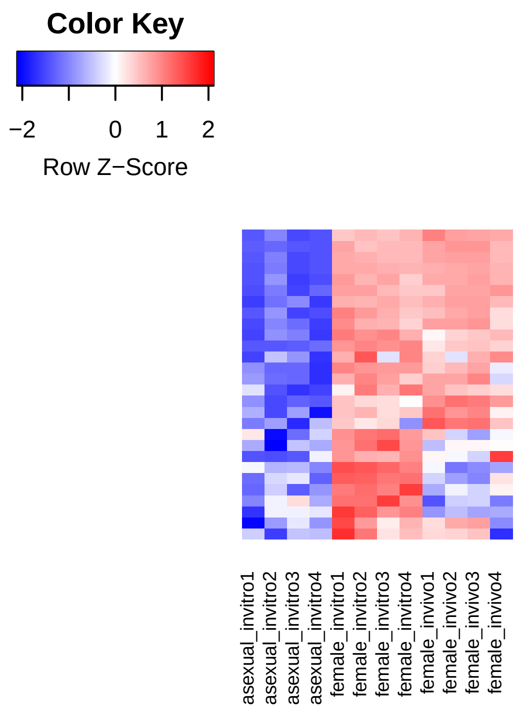

10.2.4 glycosylation

```
Glycos <- as_tibble(v.myDGEList.filtered.norm$E, rownames = "geneSymbol") %>%
  dplyr::filter(geneSymbol=="cgd5_690" |
                geneSymbol=="cgd5_3140" |
                geneSymbol=="cgd5_2590" |
                geneSymbol=="cgd5_790" |
                geneSymbol=="cgd1_3720" |
                geneSymbol=="cgd1_3730" |
                geneSymbol=="cgd3_3590" |
                geneSymbol=="cgd8_920" ) %>%
  dplyr::select(geneSymbol,asexual_invitro1, asexual_invitro2, asexual_invitro3, asexual_invitro4,
                female_invitro1, female_invitro2, female_invitro3, female_invitro4,
                female_invivo1, female_invivo2, female_invivo3, female_invivo4)

Glycos <-column_to_rownames(Glycos, var="geneSymbol")
Glycos.matrix <- data.matrix(Glycos)

hrGly <- hclust(as.dist(1-cor(t(Glycos.matrix), method="pearson")), method="complete")

heatmap.2(Glycos.matrix, Rowv=as.dendrogram(hrGly), Colv=NA,
          col=myheatcol, scale="row", density.info="none",
          trace="none", key = 1,
          cexRow=1, cexCol=0.75, margins=c(10,20),
```

```
          dendrogram = "none")
```

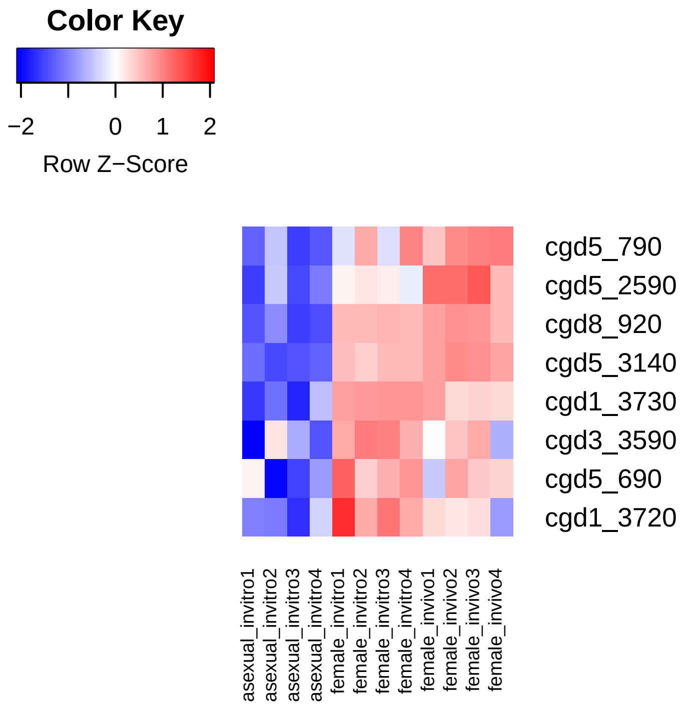

10.2.5 Polysaccharide pyruvyl transferases heatmap

```
pyruv <- as_tibble(v.myDGEList.filtered.norm$E, rownames = "geneSymbol") %>%
  dplyr::filter(geneSymbol=="cgd7_2580" |
                geneSymbol=="cgd6_1450" ) %>%
  dplyr::select(geneSymbol,asexual_invitro1, asexual_invitro2, asexual_invitro3, asexual_invitro4,
                female_invitro1, female_invitro2, female_invitro3, female_invitro4,
                female_invivo1, female_invivo2, female_invivo3, female_invivo4)

pyruv <-column_to_rownames(pyruv, var="geneSymbol")
pyruv.matrix <- data.matrix(pyruv)


hrPyr <- hclust(as.dist(1-cor(t(pyruv.matrix), method="pearson")), method="complete")

heatmap.2(pyruv.matrix, Rowv=NA, Colv=NA,
          col=myheatcol, scale="row", density.info="none",
          trace="none", key = 1,
          cexRow=1, cexCol=0.75, margins=c(10,20),
          dendrogram = "none")
```

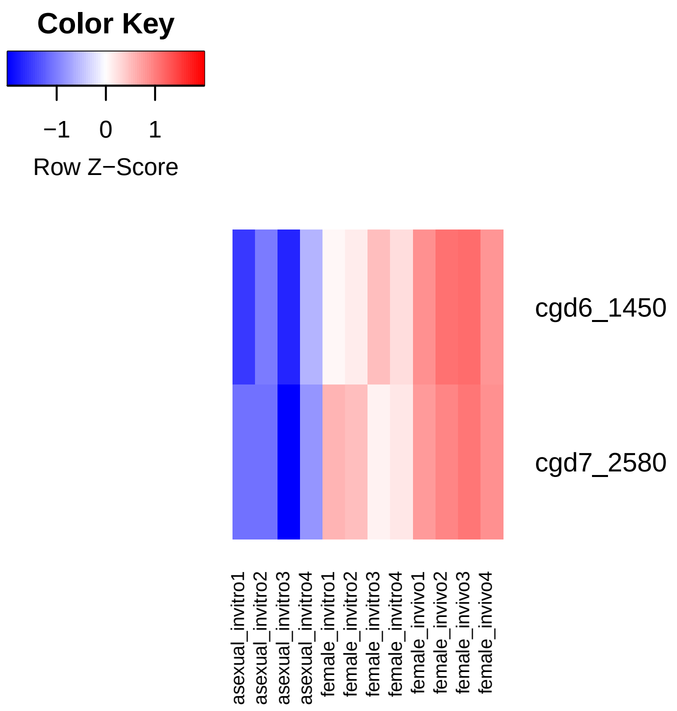

10.2.6 Fatty Acid PKS heatmap

```
PKS <- as_tibble(v.myDGEList.filtered.norm$E, rownames = "geneSymbol") %>%
  dplyr::filter(geneSymbol=="cgd3_2870" |
                geneSymbol=="cgd8_3680" |
                geneSymbol=="cgd1_3710" |
                geneSymbol=="cgd3_2180" |
                geneSymbol=="cgd8_2390" |
                geneSymbol=="cgd4_4340" |
                geneSymbol=="cgd8_1400" |
                geneSymbol=="cgd1_1110" |
                geneSymbol=="cgd4_2900" ) %>%
  dplyr::select(geneSymbol,asexual_invitro1, asexual_invitro2, asexual_invitro3, asexual_invitro4,
                female_invitro1, female_invitro2, female_invitro3, female_invitro4,
                female_invivo1, female_invivo2, female_invivo3, female_invivo4)

PKS <-column_to_rownames(PKS, var="geneSymbol")
PKS.matrix <- data.matrix(PKS)

PKShr <- hclust(as.dist(1-cor(t(PKS.matrix), method="pearson")), method="complete")

heatmap.2(PKS.matrix, Rowv=as.dendrogram(PKShr), Colv=NA,
          col=myheatcol, scale="row", density.info="none",
          trace="none", key = 1,
```

```
        cexRow=1, cexCol=0.75, margins=c(10,20),
        dendrogram = "none")
```

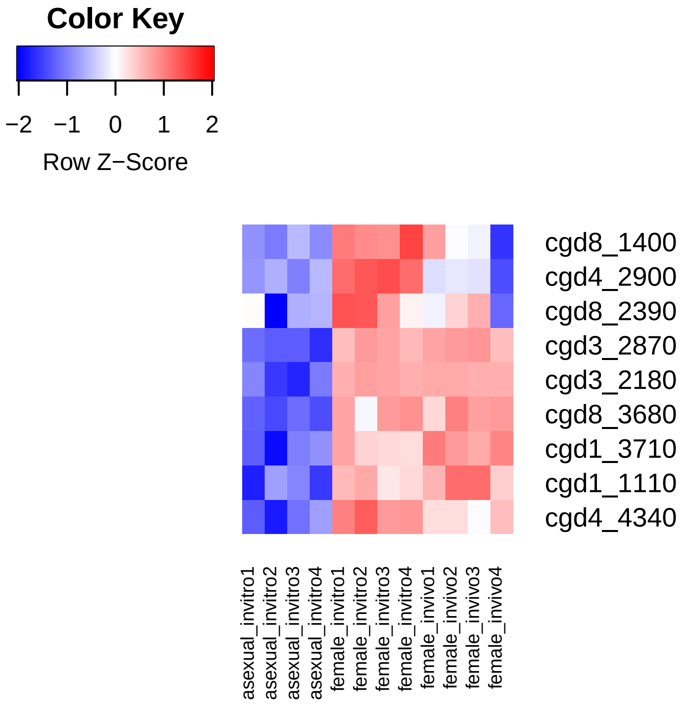

10.3 heatmap: energy storage - Figure 4C

```
myAMY <- as_tibble(v.myDGEList.filtered.norm$E, rownames = "geneSymbol") %>%
  dplyr::filter(geneSymbol=="cgd6_3750" |
                geneSymbol=="cgd5_2910" |
                geneSymbol=="cgd3_1580" |
                geneSymbol=="cgd2_3270" |
                geneSymbol=="cgd2_3260" |
                geneSymbol=="cgd2_2340" |
                geneSymbol=="cgd4_2600" |
                geneSymbol=="cgd6_2450" |
                geneSymbol=="cgd6_3280" |
                geneSymbol=="cgd8_4940" |
                geneSymbol=="cgd6_880" |
                geneSymbol=="cgd7_1830" |
                geneSymbol=="cgd7_910" |
                geneSymbol=="cgd1_3020" |
                geneSymbol=="cgd6_3790" |
                geneSymbol=="cgd6_3800" |
                geneSymbol=="cgd1_2040" |
                geneSymbol=="cgd1_3040" |
```

```
                  geneSymbol=="cgd3_1400" |
                  geneSymbol=="cgd1_3060" |
                  geneSymbol=="cgd7_4270") %>%
  dplyr::select(geneSymbol,asexual_invitro1, asexual_invitro2, asexual_invitro3, asexual_invitro4,
                female_invitro1, female_invitro2, female_invitro3, female_invitro4,
                female_invivo1, female_invivo2, female_invivo3, female_invivo4)

myAMY <-column_to_rownames(myAMY, var="geneSymbol")
myAMY.matrix <- data.matrix(myAMY)

hrAMY <- hclust(as.dist(1-cor(t(myAMY.matrix), method="pearson")), method="complete")

heatmap.2(myAMY.matrix, Rowv=as.dendrogram(hrAMY), Colv=NA,
          col=myheatcol, scale="row", density.info="none",
          trace="none", key = 1,
          cexRow=0.5, cexCol=0.75, margins=c(10,20),
          dendrogram = "none")
```

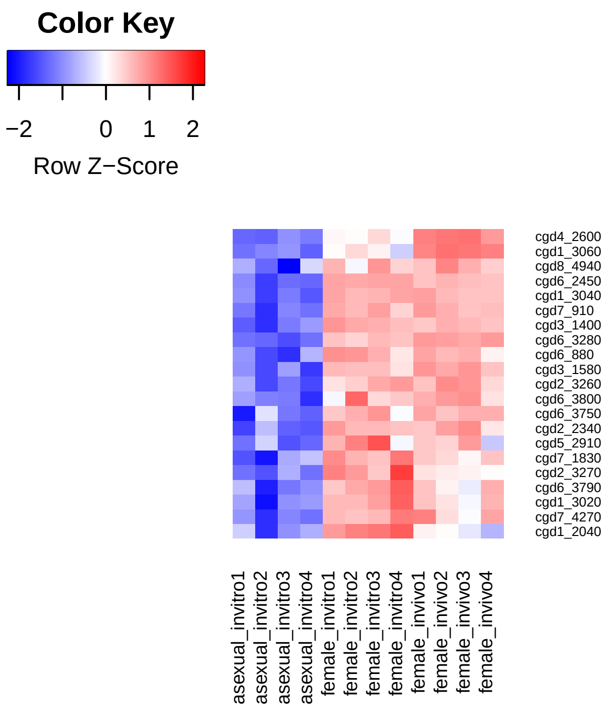

10.4 heatmap: AP2 and AP2-related genes - Figure 4D

```
myAP2 <- as_tibble(v.myDGEList.filtered.norm$E, rownames = "geneSymbol") %>%
  dplyr::filter(geneSymbol=="cgd4_1110" | geneSymbol=="cgd8_3130" |
                geneSymbol=="cgd8_3230" |geneSymbol=="cgd1_3520" |
                  geneSymbol=="cgd2_3490" | geneSymbol=="cgd4_3820" |
```

```
                    geneSymbol=="cgd4_600" | geneSymbol=="cgd5_2570" | geneSymbol=="cgd5_4250" |
                    geneSymbol=="cgd8_810" | geneSymbol=="cgd4_2950" |
                    geneSymbol=="cgd6_2600" | geneSymbol=="cgd6_2670" | geneSymbol=="cgd3_1980" |
                    geneSymbol=="cgd3_2970" | geneSymbol=="cgd6_1140" | geneSymbol=="cgd6_5320") %>%
  dplyr::select(geneSymbol,asexual_invitro1, asexual_invitro2, asexual_invitro3, asexual_invitro4,
                female_invitro1, female_invitro2, female_invitro3, female_invitro4,
                female_invivo1, female_invivo2, female_invivo3, female_invivo4)

myAP2 <-column_to_rownames(myAP2, var="geneSymbol")
myAP2.matrix <- data.matrix(myAP2)

hrAP2 <- hclust(as.dist(1-cor(t(myAP2.matrix), method="pearson")), method="complete")

heatmap.2(myAP2.matrix, Rowv=as.dendrogram(hrAP2), Colv=NA,
          col=myheatcol, scale="row", density.info="none",
          trace="none", key = 1,
          cexRow=0.5, cexCol=0.75, margins=c(10,20),
          dendrogram = "none")
```

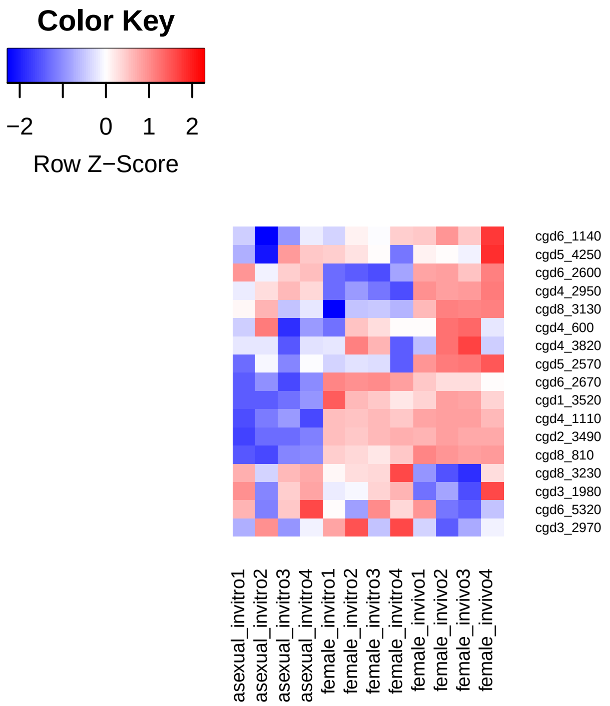

11 Global analysis that incorporates all samples across all experiments

```
load("Txi_gene")
myDGEList <- DGEList(Txi_gene$counts)
# use the 'cpm' function from EdgeR to get counts per million
log2.cpm <- cpm(myDGEList, log=TRUE)
log2.cpm.df <- as_tibble(log2.cpm)
colnames(log2.cpm.df) <- SampleLabels
log2.cpm.df <- melt(log2.cpm.df)
colnames(log2.cpm.df) <- c("sample", "expression")

p1 <- ggplot(log2.cpm.df, aes(x=sample, y=expression, fill=sample)) +
  geom_violin(trim = FALSE, show.legend = FALSE) +
  stat_summary(fun.y = "median", geom = "point", shape = 124, size = 6, color = "black", show.legend = F
  labs(y="log2 expression", x = "sample",
       title = "raw data") +
  coord_flip() +
  theme_bw()

cpm <- cpm(myDGEList)
#keeping only genes with > 10 cpm in at least 3 samples
keepers <- rowSums(cpm>10)>=3
myDGEList.filtered <- myDGEList[keepers,]
myDGEList.filtered.norm <- calcNormFactors(myDGEList.filtered, method = "TMM")
log2.cpm.filtered.norm <- cpm(myDGEList.filtered.norm, log=TRUE)
log2.cpm.filtered.norm.df <- as_tibble(log2.cpm.filtered.norm)
colnames(log2.cpm.filtered.norm.df) <- SampleLabels
log2.cpm.filtered.norm.df <- melt(log2.cpm.filtered.norm.df)
colnames(log2.cpm.filtered.norm.df) <- c("sample", "expression")

normData <- as_tibble(log2.cpm.filtered.norm, rownames = "geneSymbol")
colnames(normData) <- c("geneSymbol", SampleLabels)
write_tsv(normData, "normData_combo.txt")

p2 <- ggplot(log2.cpm.filtered.norm.df, aes(x=sample, y=expression, fill=sample)) +
  geom_violin(trim = FALSE, show.legend = FALSE) +
  stat_summary(fun.y = "median", geom = "point", shape = 124, size = 6,
               color = "black", show.legend = FALSE) +
  labs(y="log2 expression", x = "sample",
       title = "filtered, normalized data") +
  coord_flip() +
  theme_bw()

plot_grid(p1, p2, labels = c("A", "B"))
```

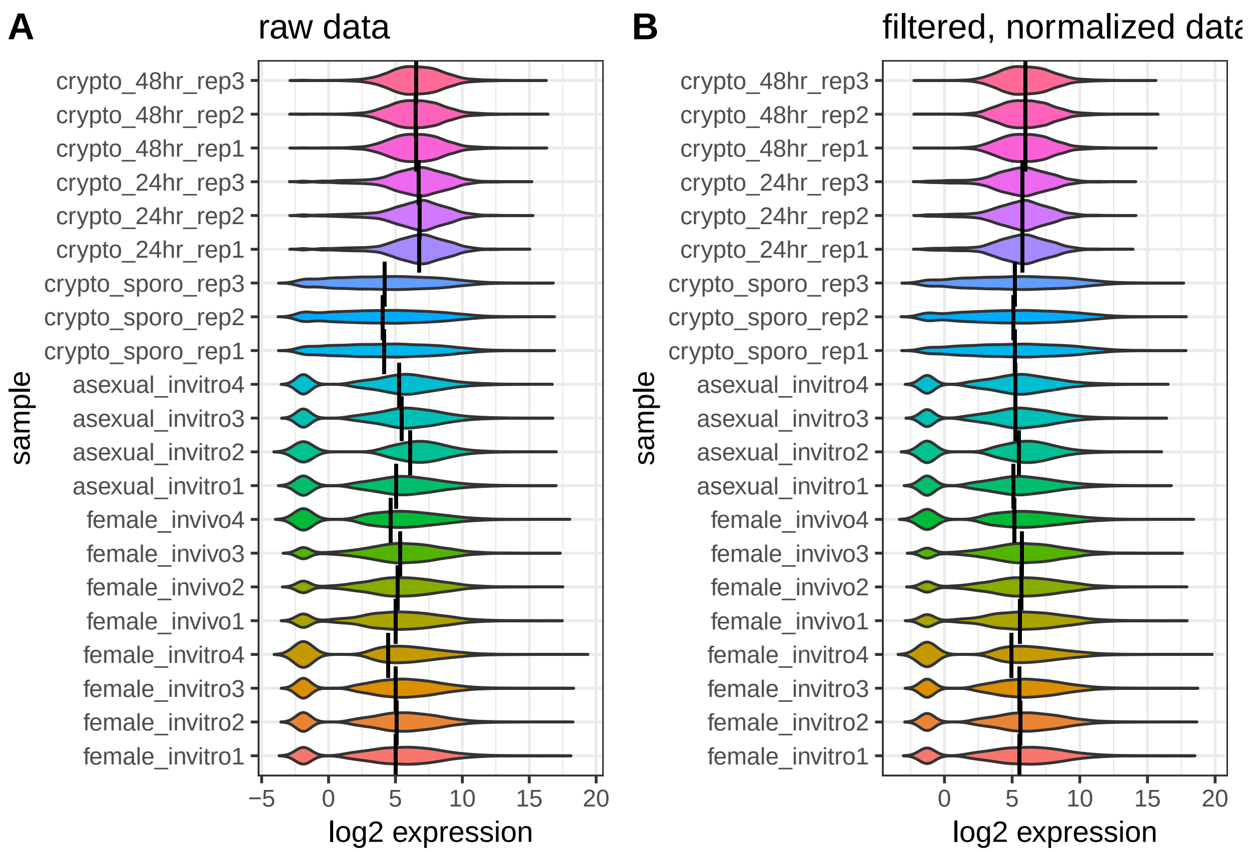

11.1 PCA showing batch effect

```
pca.res <- prcomp(t(log2.cpm.filtered.norm), scale.=F, retx=T)
pc.var<-pca.res$sdev^2
pc.per<-round(pc.var/sum(pc.var)*100, 1)

pca.res.df <- as_tibble(pca.res$x)

p1 <- ggplot(pca.res.df, aes(x=PC1, y=PC2, color=sex, shape=origin)) +
  geom_point(size=3) +
  theme(legend.position="right") +
  xlab(paste0("PC1 (",pc.per[1],"%",")")) +
  ylab(paste0("PC2 (",pc.per[2],"%",")")) +
  theme_bw() +
  coord_fixed()

p2 <- ggplot(pca.res.df, aes(x=PC1, y=PC2, color=batch)) +
  geom_point(size=3) +
  theme(legend.position="right") +
  xlab(paste0("PC1 (",pc.per[1],"%",")")) +
  ylab(paste0("PC2 (",pc.per[2],"%",")")) +
  theme_bw() +
  coord_fixed()
```

```
plot_grid(p1, p2, labels = c("A", "B"))
```

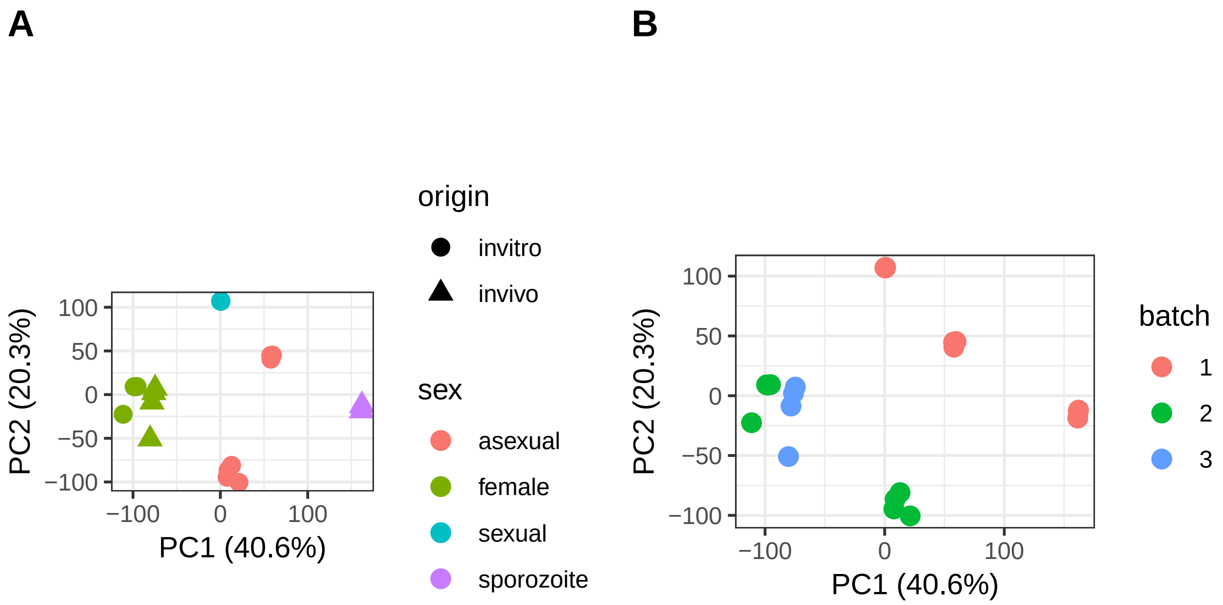

11.2 PCA after correcting for batch effect - Figure 3D

```
log2.cpm.filtered.norm <- t(cpm(myDGEList.filtered.norm, log=TRUE))
log2.cpm.filtered.norm.batchCorrected <- empiricalBayesLM(
  log2.cpm.filtered.norm,
  removedCovariates = targets$batch,
  fitToSamples = targets$sex_stage=="asexual")$adjustedData;

pca.res <- prcomp(log2.cpm.filtered.norm.batchCorrected, scale.=F, retx=T)
pc.var<-pca.res$sdev^2
pc.per<-round(pc.var/sum(pc.var)*100, 1)
pca.res.df <- as_tibble(pca.res$x)

ggplot(pca.res.df, aes(x=PC1, y=PC2, color=sex, shape=origin)) +
  geom_point(size=6) +
  theme(legend.position="right") +
  xlab(paste0("PC1 (",pc.per[1],"%",")")) +
  ylab(paste0("PC2 (",pc.per[2],"%",")")) +
  labs(title="PCA showing all datasets combined",
       subtitle = "Principal component analysis (PCA) \nshowing all samples after batch correction. \nCl
       \nThis plot corresponds to Figure 3D in the manuscript.") +
  theme_bw() +
```

```
  theme(axis.text=element_text(size=16),
        axis.title=element_text(size=18),
        legend.text=element_text(size=14),
        legend.title=element_text(size=16, face="bold"),
        plot.title = element_text(face="bold"))
```

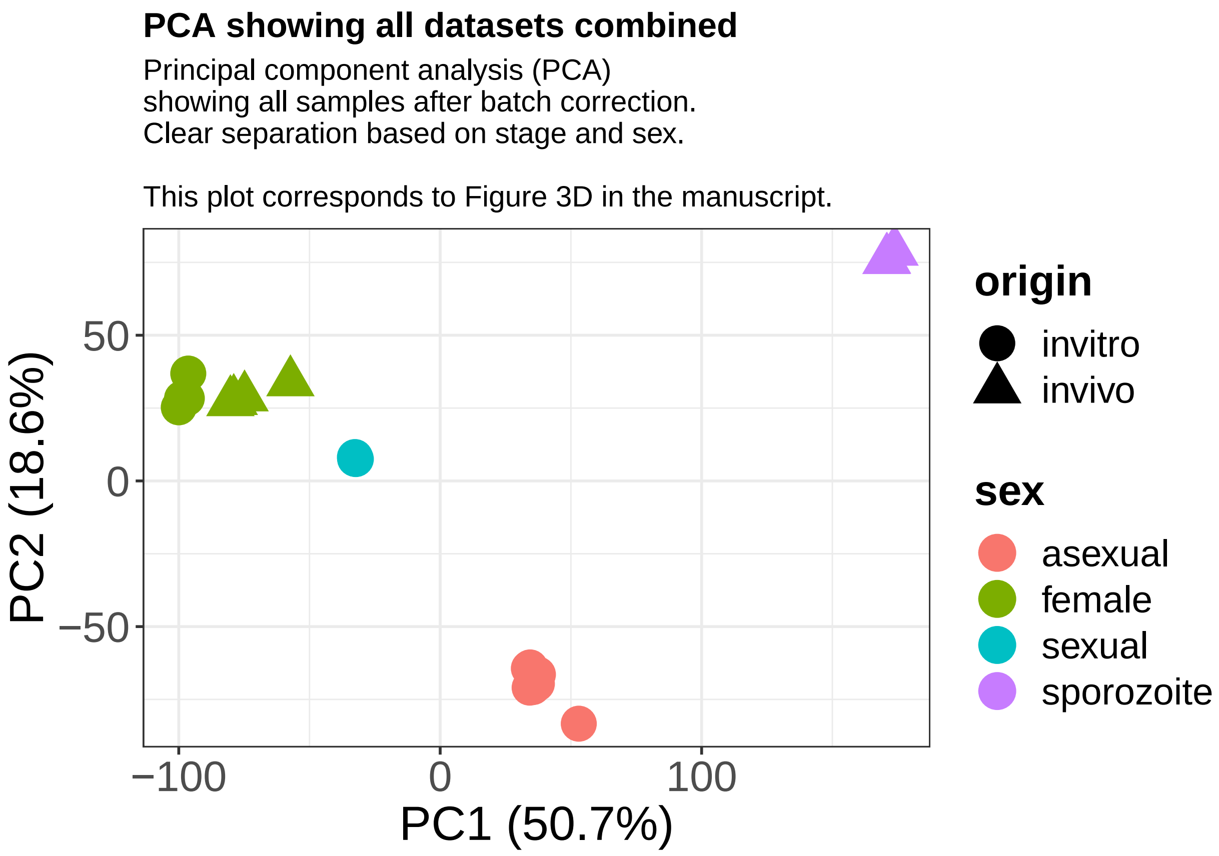

11.3 Creating DGEList from batch corrected counts

```
design <- model.matrix(~0 + group)
colnames(design) <- levels(group)

#need to convert batch corrected Log2 CPM back into counts to create a DGEList for differential testing
log2.cpm.filtered.norm.batchCorrected <- t(log2.cpm.filtered.norm.batchCorrected)
cpm.filtered.norm.batchCorrected <- 2^(log2.cpm.filtered.norm.batchCorrected)
sample1 <- (as.matrix(cpm.filtered.norm.batchCorrected[,1]*colSums(myDGEList.filtered.norm$counts)[1]))
sample2 <- (as.matrix(cpm.filtered.norm.batchCorrected[,2]*colSums(myDGEList.filtered.norm$counts)[2]))
sample3 <- (as.matrix(cpm.filtered.norm.batchCorrected[,3]*colSums(myDGEList.filtered.norm$counts)[3]))
sample4 <- (as.matrix(cpm.filtered.norm.batchCorrected[,4]*colSums(myDGEList.filtered.norm$counts)[4]))
sample5 <- (as.matrix(cpm.filtered.norm.batchCorrected[,5]*colSums(myDGEList.filtered.norm$counts)[5]))
sample6 <- (as.matrix(cpm.filtered.norm.batchCorrected[,6]*colSums(myDGEList.filtered.norm$counts)[6]))
sample7 <- (as.matrix(cpm.filtered.norm.batchCorrected[,7]*colSums(myDGEList.filtered.norm$counts)[7]))
sample8 <- (as.matrix(cpm.filtered.norm.batchCorrected[,8]*colSums(myDGEList.filtered.norm$counts)[8]))
sample9 <- (as.matrix(cpm.filtered.norm.batchCorrected[,9]*colSums(myDGEList.filtered.norm$counts)[9]))
sample10 <- (as.matrix(cpm.filtered.norm.batchCorrected[,10]*colSums(myDGEList.filtered.norm$counts)[10
```

```
sample11 <- (as.matrix(cpm.filtered.norm.batchCorrected[,11]*colSums(myDGEList.filtered.norm$counts)[11]
sample12 <- (as.matrix(cpm.filtered.norm.batchCorrected[,12]*colSums(myDGEList.filtered.norm$counts)[12]
sample13 <- (as.matrix(cpm.filtered.norm.batchCorrected[,13]*colSums(myDGEList.filtered.norm$counts)[13]
sample14 <- (as.matrix(cpm.filtered.norm.batchCorrected[,14]*colSums(myDGEList.filtered.norm$counts)[14]
sample15 <- (as.matrix(cpm.filtered.norm.batchCorrected[,15]*colSums(myDGEList.filtered.norm$counts)[15]
sample16 <- (as.matrix(cpm.filtered.norm.batchCorrected[,16]*colSums(myDGEList.filtered.norm$counts)[16]
sample17 <- (as.matrix(cpm.filtered.norm.batchCorrected[,17]*colSums(myDGEList.filtered.norm$counts)[17]
sample18 <- (as.matrix(cpm.filtered.norm.batchCorrected[,18]*colSums(myDGEList.filtered.norm$counts)[18]
sample19 <- (as.matrix(cpm.filtered.norm.batchCorrected[,19]*colSums(myDGEList.filtered.norm$counts)[19]
sample20 <- (as.matrix(cpm.filtered.norm.batchCorrected[,20]*colSums(myDGEList.filtered.norm$counts)[20]
sample21 <- (as.matrix(cpm.filtered.norm.batchCorrected[,21]*colSums(myDGEList.filtered.norm$counts)[21]

counts.batchCorrected <- cbind(sample1, sample2, sample3,
                               sample4, sample5, sample6,
                               sample7, sample8, sample9,
                               sample10, sample11, sample12,
                               sample13, sample14, sample15,
                               sample16, sample17, sample18,
                               sample19, sample20, sample21)

myDGEList.batchCorrected <- DGEList(counts.batchCorrected)

v.DEGList.batchCorrected <- voom(myDGEList.batchCorrected, design, plot = FALSE)
fit <- lmFit(v.DEGList.batchCorrected, design)
#setting up contrast matrix for three pairwise comparisons
contrast.matrix <- makeContrasts(sporozoite.vs.female.invitro = sporozoite_invivo - female_invivo,
                                 sporozoite.vs.asexual = sporozoite_invivo - asexual_invitro,
                                 sporozoite.vs.sexual = sporozoite_invivo - sexual_invitro,
                                 levels=design)

fits <- contrasts.fit(fit, contrast.matrix)
ebFit <- eBayes(fits)
```

---

12 Session info

The output from running 'sessionInfo' is shown below and details all packages and versions used in this script.

```
sessionInfo()
```

```
## R version 3.6.0 (2019-04-26)
## Platform: x86_64-apple-darwin15.6.0 (64-bit)
## Running under: macOS Mojave 10.14.5
##
## Matrix products: default
## BLAS:   /Library/Frameworks/R.framework/Versions/3.6/Resources/lib/libRblas.0.dylib
## LAPACK: /Library/Frameworks/R.framework/Versions/3.6/Resources/lib/libRlapack.dylib
##
## locale:
## [1] en_US.UTF-8/en_US.UTF-8/en_US.UTF-8/C/en_US.UTF-8/en_US.UTF-8
##
## attached base packages:
## [1] stats     graphics  grDevices utils     datasets  methods   base
```

```
##
## other attached packages:
##  [1] WGCNA_1.68           fastcluster_1.1.25    dynamicTreeCut_1.63-1
##  [4] cowplot_0.9.4        gt_0.1.0              gplots_3.0.1.1
##  [7] matrixStats_0.54.0   edgeR_3.26.0          limma_3.40.0
## [10] genefilter_1.66.0    RColorBrewer_1.1-2    tximport_1.12.0
## [13] reshape2_1.4.3       forcats_0.4.0         stringr_1.4.0
## [16] dplyr_0.8.1          purrr_0.3.2           readr_1.3.1
## [19] tidyr_0.8.3          tibble_2.1.2          ggplot2_3.1.1
## [22] tidyverse_1.2.1      knitr_1.23            rmarkdown_1.13
##
## loaded via a namespace (and not attached):
##   [1] colorspace_1.4-1      htmlTable_1.13.1      base64enc_0.1-3
##   [4] rstudioapi_0.10       bit64_0.9-7           mvtnorm_1.0-10
##   [7] AnnotationDbi_1.46.0  lubridate_1.7.4       xml2_1.2.0
##  [10] codetools_0.2-16      splines_3.6.0         doParallel_1.0.14
##  [13] impute_1.58.0         robustbase_0.93-5     Formula_1.2-3
##  [16] jsonlite_1.6          broom_0.5.2           annotate_1.62.0
##  [19] cluster_2.0.9         GO.db_3.8.2           rrcov_1.4-7
##  [22] compiler_3.6.0        httr_1.4.0            backports_1.1.4
##  [25] assertthat_0.2.1      Matrix_1.2-17         lazyeval_0.2.2
##  [28] cli_1.1.0             acepack_1.4.1         htmltools_0.3.6
##  [31] tools_3.6.0           gtable_0.3.0          glue_1.3.1
##  [34] Rcpp_1.0.1            Biobase_2.44.0        cellranger_1.1.0
##  [37] gdata_2.18.0          preprocessCore_1.46.0 nlme_3.1-140
##  [40] iterators_1.0.10      xfun_0.7              rvest_0.3.4
##  [43] gtools_3.8.1          XML_3.99-0            DEoptimR_1.0-8
##  [46] MASS_7.3-51.4         scales_1.0.0          hms_0.4.2
##  [49] parallel_3.6.0        rhdf5_2.28.0          yaml_2.2.0
##  [52] memoise_1.1.0         gridExtra_2.3         sass_0.1.0.9000
##  [55] rpart_4.1-15          latticeExtra_0.6-28   stringi_1.4.3
##  [58] RSQLite_2.1.1         S4Vectors_0.22.0      pcaPP_1.9-73
##  [61] foreach_1.4.4         checkmate_1.9.3       caTools_1.17.1.2
##  [64] BiocGenerics_0.30.0   rlang_0.3.4           pkgconfig_2.0.2
##  [67] commonmark_1.7        bitops_1.0-6          evaluate_0.14
##  [70] lattice_0.20-38       Rhdf5lib_1.6.0        labeling_0.3
##  [73] htmlwidgets_1.3       bit_1.1-14            tidyselect_0.2.5
##  [76] robust_0.4-18         plyr_1.8.4            magrittr_1.5
##  [79] R6_2.4.0              fit.models_0.5-14     IRanges_2.18.0
##  [82] generics_0.0.2        Hmisc_4.2-0           DBI_1.0.0
##  [85] pillar_1.4.1          haven_2.1.0           foreign_0.8-71
##  [88] withr_2.1.2           survival_2.44-1.1     RCurl_1.95-4.12
##  [91] nnet_7.3-12           modelr_0.1.4          crayon_1.3.4
##  [94] KernSmooth_2.23-15    locfit_1.5-9.1        grid_3.6.0
##  [97] readxl_1.3.1          data.table_1.12.2     blob_1.1.1
## [100] digest_0.6.19         xtable_1.8-4          stats4_3.6.0
## [103] munsell_0.5.0
```
